# Arene binding by silazido heavier alkaline earth cations

**DOI:** 10.1039/d6dt02345b

**Published:** 2026-10-29

**Authors:** Oliver Jarvis, Jacob T. Guntley, Michael S. Hill, Mary F. Mahon, Kyle G. Pearce, Claire L. McMullin

**Affiliations:** a Department of Chemistry, University of Bath Claverton Down Bath BA2 7AY UK msh27@bath.ac.uk cm2025@bath.ac.uk

## Abstract

Reactions of [Ph_3_C]^+^[Al{OC(CF_3_)_3_}_4_]^−^ with [Ae{N(SiMe_3_)_2_}_2_]_2_ (Ae = Ca, Sr) or [Ba{N(SiMe_3_)_2_}_2_(THF)]_2_ have been studied in benzene, mesitylene and chlorobenzene. Irrespective of Ae identity, interrogation of the bulk constitutions of the resultant systems was complicated by liquid clathrate formation. X-ray diffraction analysis of single crystals, however, identified the formation of bimetallic dications, [(η^6^-C_6_H_6_)Ae-μ-N(SiMe_3_)_2_]_2_^2+^, and monometallic monocations, [(η^6^-1,3,5-Me_3_C_6_H_3_)_2_Ae-κ^1^-N(SiMe_3_)_2_]^+^ from benzene and mesitylene, respectively. In contrast, only the strontium system provided any indication of similar binding of chlorobenzene. Although these observations infer a greater binding affinity for the more electron rich arene derivative, in no case could any significant perturbation of the arene structure be identified. DFT calculations on the isolated cationic structures are consistent with this viewpoint, confirming the energetic similarity of both structural types and describing a decreasing affinity for arene binding in the order Ca > Sr > Ba. These calculations also invoke a notable level of *n*d-orbital participation in arene binding by [(Me_3_Si)_2_NAe(η^6^-arene)]^+^, which is all but negated by the adoption of an alternative silazide-bridged structure.

## Abstract 2

Reactions of [Ph_3_C]^+^[Al{OC(CF_3_)_3_}_4_]^−^ with heavier alkaline earth silazides provide bimetallic dications, [(η^6^-C_6_H_6_)Ae-μ-N(SiMe_3_)_2_]_2_^2+^ and monometallic monocations, [(η^6^-1,3,5-Me_3_C_6_H_3_)_2_Ae-κ^1^-N(SiMe_3_)_2_]^+^ from benzene and mesitylene, respectively.

## Graphical abstract

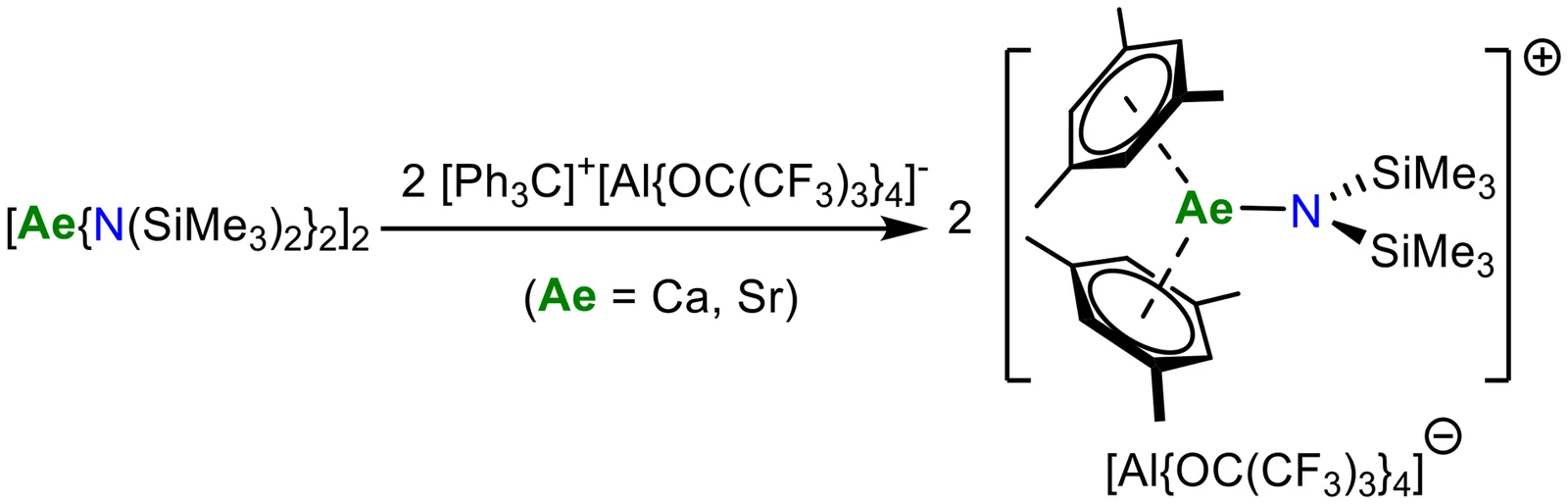

## Introduction

After a century in the shadow of organomagnesium reagents,^1^ interest in alkyl and aryl derivatives of the heavier alkaline earth (Ae) elements (Ae = Ca, Sr or Ba) has flourished during the past two decades.^2–6^ Although the resultant chemistry has been applicable to a wide variety of stoichiometric and catalytic contexts,^7,8^ a subsection of notably striking observations arise from reactivity toward arene-based substrates.^9–13^ Relevant to several biological systems,^14,15^ theoretical investigations have demonstrated that the gas phase η^6^-binding of benzene and toluene by Ca^2+^ cations (*e.g.*I, Fig. 1) can approach 70 kcal mol^−1^.^16–25^ With respect to solution-phase reactivity, similar, but transient, non-covalent interactions have been implicated in the nucleophilic substitution of benzene by hydrido- and organoderivatives of calcium and strontium.^9,11,13^ These observations imply the Ae^2+^ cations exert a dual influence, as both the agent of delivery for the hydride or organic anion and by activating the electron-rich π system toward otherwise energetically inaccessible nucleophilic attack. Although the structural characterisation of intramolecular Ae⋯arene interactions to an aryl or aryl-appended anion is a commonly characterised phenomenon in molecular Ae compounds,^5,26–33^ the coordination of a non-functionalised arene to charge neutral molecular Ae species remains a relative rarity, especially for the less polarisable cations of calcium and strontium.^34–37^

**Fig. 1:**
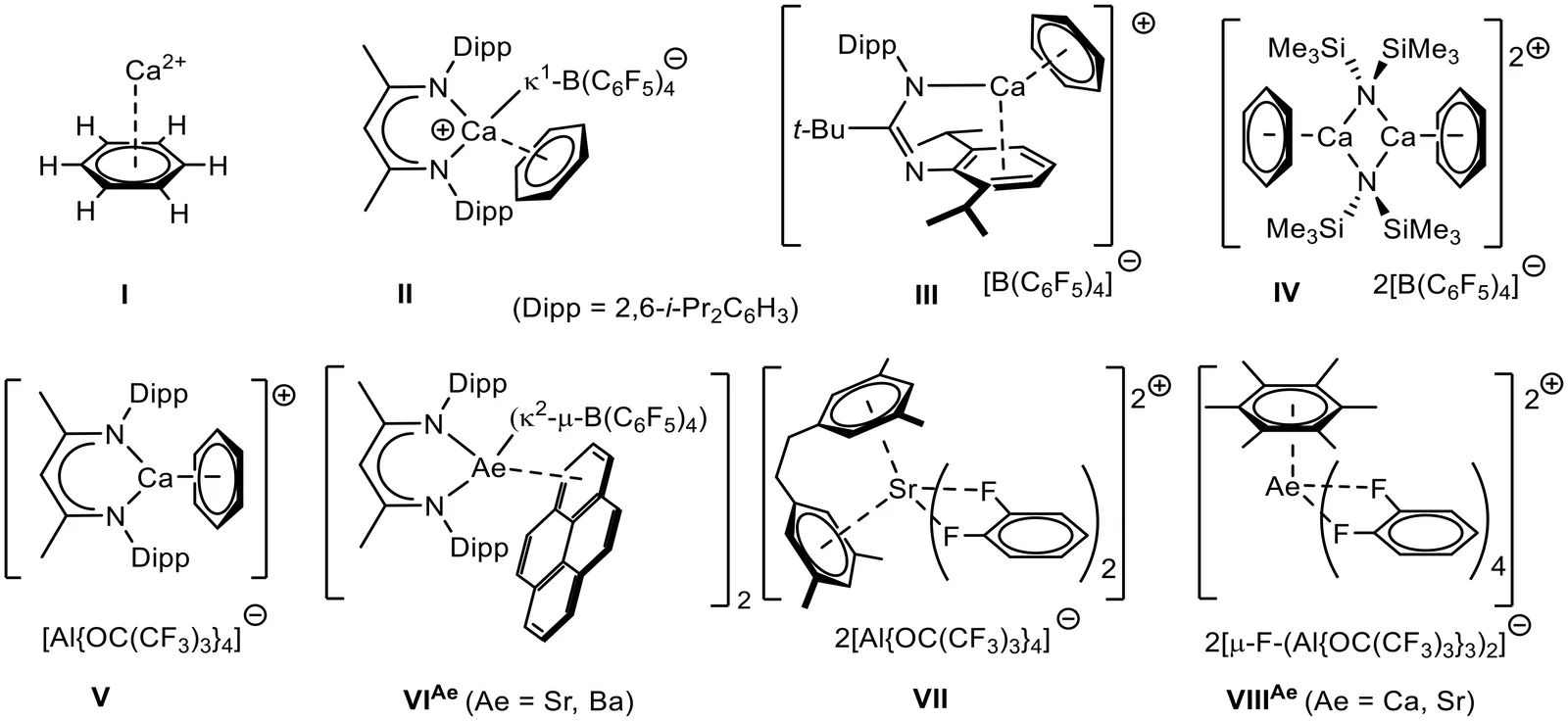
Cationic alkaline earth complexes.

Group 2-arene coordination by design, therefore, has generally required the elaboration of condensed-phase analogues of I, *viz*., the synthesis of mono- and dicationic Ae species in conjunction with a weakly coordinating anion to ‘tame’ its innate electrophilicity.^38^ To this end, Harder and co-workers have employed [B(C_6_F_5_)_4_]^−^ in the isolation of species such as II and III (Fig. 1),^39,40^ wherein the bulky supporting β-diketiminate (BDI) and amidinate ligands maintain the unimolecular constitution of the calcium cation, but which retains sufficient coordinative unsaturation to sequester a molecule of benzene. Of direct relevance to the current contribution, Hinz has very recently reported that the silazide-bridged and η^6^-benzene coordinated dicalcium dication (IV) may be isolated from the reaction of a carbazolyl derivative with [(Et_3_Si)_2_H]^+^[B(C_6_F_5_)_4_]^−^, albeit the fate of the N-heterocyclic ligand could not be determined.^41^ Our own work has utilised the same principles for the synthesis of calcium species, including V, in which charge balance is maintained by Krossing's aluminate anion, [Al{OC(CF_3_)_3_}_4_]^−^.^42,43^

With regard to the heaviest Ae elements, attempts by the Harder group to access strontium and barium analogues of II and III were thwarted by liquid clathrate formation.^44^ Addition of the tetracyclic aromatic hydrocarbon, pyrene, however, enabled the isolation of [(BDI)Ae-η^3^-pyrene]_2_^+^[B(C_6_F_5_)_4_]_2_^−^ (VI^Ae^, Ae = Sr, Ba), both of which aggregate as dications with the group 2 centres linked by Ae⋯F–C interactions provided by two bridging [B(C_6_F_5_)_4_]^−^ anions_._ The very weakly coordinating nature of the [Al{OC(CF_3_)_3_}_4_]^−^ anion was emphasised by Krossing and co-workers’ successful characterisation of the dicationic strontium complex (VII), which was isolated through the introduction of a neutral chelating *ansa*-bis-arene ligand.^45^ Although the same group's attempts to access hexamethylbenzene-coordinated Ca, Sr and Ba cations by oxidation of the metals with the radical cation salt, [C_6_Me_6_]˙^+^[Al{OC(CF_3_)_3_}_4_]^−^, resulted in anion degradation and the isolation of species presenting monodentate Ae–μ-F–Al coordination of [F-Al{OC(CF_3_)_3_}_3_]^−^, recourse to [C_6_Me_6_]˙^+^[μ-F-(Al{OC(CF_3_)_3_}_3_)_2_]^−^ provided the Ca and Sr cations, VIII^Ae^ (Fig. 1, Ae = Ca, Sr), in which the coordination spheres of the group 2 centres are completed by four Ae⋯*o*-(F–C)_2_ bidentate molecules of *ortho*-difluorobenzene.^46^

In the absence of any potential for metal-to-ligand π back-donation, such interactions are unlikely to ever convey the consequential stereoelectronic impacts on structure, bonding and reactivity exerted by transition metal-to-arene binding.^47–49^ Indeed, quantum theory of atoms in molecules (QTAIM) analyses have indicated that the arene binding of the species depicted in Fig. 1 is largely electrostatic in origin.^44,46^ Reminiscent of earlier discussion of the origins of hypervalency in heavier p-block compounds,^50–54^ however, the potential for d-orbital participation in the chemistry of atomic Ae(0) species has emerged as a topic for debate.^55–57^ In common with other recent theoretical considerations of Ae species,^58–65^ the computed molecular orbitals of VII and VIII^Ae^ have also implicated a significant degree of ligand-to-metal π-donation from the arene HOMO to the relevant Ae (*n* − 1)d-orbitals.^45,46^ Similarly, computational analysis indicated that 3d π-engagement to the degenerate arene HOMO orbitals of benzene amounts to *ca*. 8.5% of the total arene binding to calcium in V.^42^*In silico* extension to β-diketiminato Sr and Ba analogues of V supported the persistence of this bonding feature, albeit the calculations presented an incremental decrease in relevance with increasing Ae^2+^ atomic weight (Sr 5.3%; Ba 2.9%) and synthetic attempts to realise such species were frustrated by the increasingly labile nature of the heavier Ae dications.^66^

Although a tentative consensus has, therefore, begun to emerge with regard to the exemplary cationic species summarised in Fig. 1, more coherent analysis is hindered by the disparate constitutions of the supporting organic ligands and variations in the influence of the weakly coordinating anion. In this contribution, therefore, we address this deficiency by dispensing with the BDI supporting ligand to achieve the synthesis of self-consistent series of silazido-based Ca, Sr and Ba cations, which, in conjunction with the [Al{OC(CF_3_)_3_}_4_]^−^ anion, adopt mono- or dinuclear structures dependent upon the identity of the coordinated arene.

## Results and discussion

### Benzene coordination

The synthesis of compound V was performed in benzene through the reaction of [(BDI)CaN(SiMe_3_)_2_] and [Ph_3_C]^+^[Al{OC(CF_3_)_3_}_4_]^−^, which took place alongside addition of the bis(trimethylsilyl)amide anion to a *para*-phenyl carbon centre of the trityl cation. We, thus, speculated that elimination of the same molecular by-product would result from reactions between the trityl aluminate and the base-free homoleptic derivatives, [Ae{N(SiMe_3_)_2_}_2_]_2_ (IX^Ae^; Scheme 1; Ae = Ca, Sr).^67^ Although the analogous amidobarium species (IX^Ba^) has been characterised,^68^ in our hands the metathetical route applied to the synthesis of IX^Ca^ and IX^Sr^ invariably results in contamination by variable proportions of potassium bariate species. For this reason, the current study utilised the barium THF adduct, [Ba{N(SiMe_3_)_2_}_2_(THF)]_2_ (X), which may be crystallised more reliably as the monometallic starting material.^68^

**Scheme 1:**
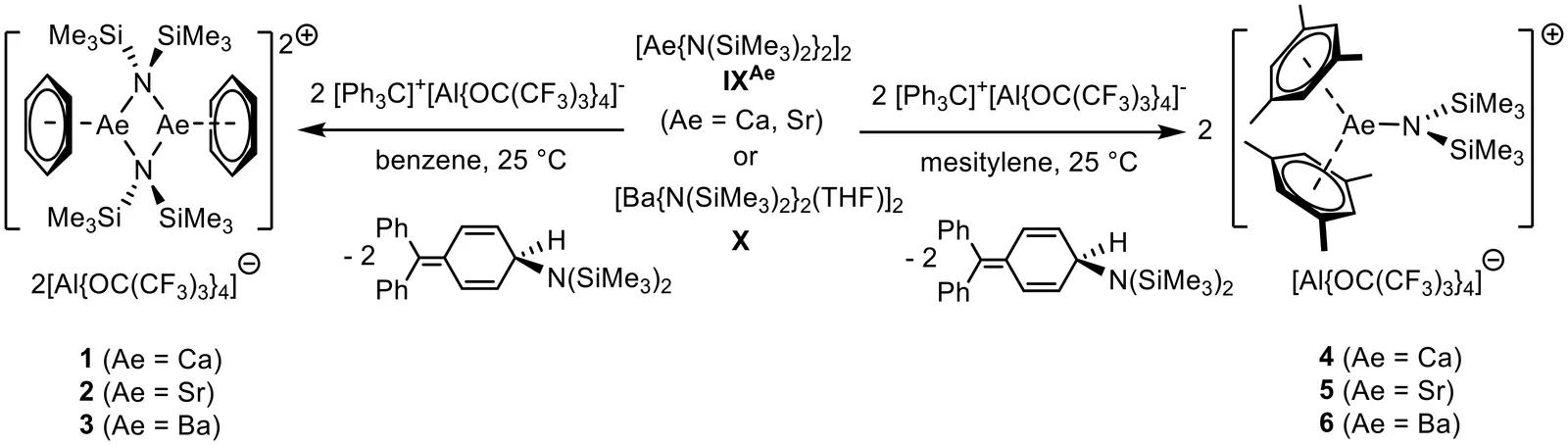
Synthesis of compounds 1–6.

Irrespective of the constitution of the alkaline earth starting material, however, an initial series of reactions performed in benzene between IX^Ae^ or X and [Ph_3_C]^+^[Al{OC(CF_3_)_3_}_4_]^−^ in a 1 : 2 ratio proceeded with the similar formation of a pale red solution atop an intensely red and more viscous layer characteristic of liquid clathrate formation.^69,70^ The semi-heterogeneous and biphasic nature of these systems meant that the acquisition of meaningful NMR spectra of pure crystalline species (*vide infra*) was invariably problematic. Bulk samples isolated by mechanical separation either showed a tendency to redissolve as a two-phase clathrate or proved inseparable from traces of the more viscous clathrate phase and, thus, of consistently variable constitution. In spite of these difficulties, ^1^H NMR spectra in either *d*_6_-benzene (1 and 2) or *d*_8_-toluene (3) allowed the identification of predominant newly formed bis(trimethylsilyl)amide environments (1, Ae = Ca, *δ*_H_ 0.10: 2, Sr, 0.10; 3, Ba, 0.09 ppm), albeit the data for 3 more likely represent the toluene-coordinated rather than the benzene-coordinated structure identified by the X-ray analysis (*vide infra*). While these data were somewhat diagnostic of the formation of the respective ionic species, 1–3, meaningful ^13^C NMR spectra could not be recorded. This deduction, however, was supported by the corresponding ^19^F NMR data, all of which presented a single resonance at an effectively identical frequency to that previously identified for the aluminate component of V (*δ*_F_ = −74.80; 1, −74.81; 2, −74.85; 3, −74.86 ppm). The solid-state constitutions of 1–3 were confirmed by analysis of red single crystals grown by undisturbed storage of the viscous components of the reaction solutions. Although sufficient material could not be isolated for either microanalysis or a meaningful estimate of the isolated yields of each compound, the crystalline structures were confirmed by mechanical separation of individual single crystals suitable for X-ray diffraction analysis.

The results of these experiments are presented in Fig. 2 with selected metric data summarised in Table 1. The analyses revealed that compounds 1–3, like that of IV, each crystallise as dimeric dications in which two alkaline earth centres are connected by twofold Ae–μ-N–Ae bridging interactions provided by the nitrogen centres of the remaining silazido anions. Compound 3 is notable, therefore, in excluding any of the coordinated THF that was introduced as a necessary component of the amidobarium starting material (X). The resultant Ae–N bond distances present some minor variations across the measurements for each individual compound (Table 1) but, reflective of the formal +1 charge borne by the group 2 centres, are contracted by *ca.* 0.1–0.15 Å in comparison to the equivalent Ae–μ-N–Ae bond lengths reported for the three [Ae{N(SiMe_3_)_2_}_2_]_2_ derivatives (IX^Ae^, Ae–μ-N: Ae = Ca,^71^ 2.482(6), 2.520(6); Ae = Sr,^72^ 2.65(1), 2.67(1); Ae = Ba,^68^ 2.798(3), 2.846(4) Å). Charge balance is, in each case, maintained by two non-coordinated [Al{OC(CF_3_)_3_}_4_]^−^ anions and, as in the structures of compounds II–V (Fig. 1), the resultant coordinative unsaturation of each alkaline earth centre is satisfied through the η^6^-sequestration of a single molecule of benzene solvent. Although there is some internal variation across the various Ae1- and Ae2-to-benzene centroid distances within the individual structures, the alkaline earth-to-carbon distances across the three compounds describe an increasing trend of *ca.* 0.1 Å that is generally reflective of the relevant Ae^2+^ ionic radii (Table 1).^73^ Potentially a consequence of its dimeric and dicationic constitution, the individual Ca–C interactions in 1 [range, 2.950(10) to 3.024(10) Å] are somewhat elongated relative to the comparable distances displayed by both compound V (avg. *ca*. 2.93 Å) and those of a further monocationic but heterobimetallic (Mg_2_Ca) species (avg. *ca.* 2.89 Å), both of which are similarly coordinated by benzene and comprise the same aluminate anion.^42,43^ The Ca–benzene interactions are evidently quite elastic as both Ca–centroid distances in 1 [2.657(4), 2.639(4) Å] are significantly elongated in comparison to the analogous derivative, IV (2.573 Å), which contains the [B(C_6_F_5_)_4_]^−^ anion.

**Fig. 2:**
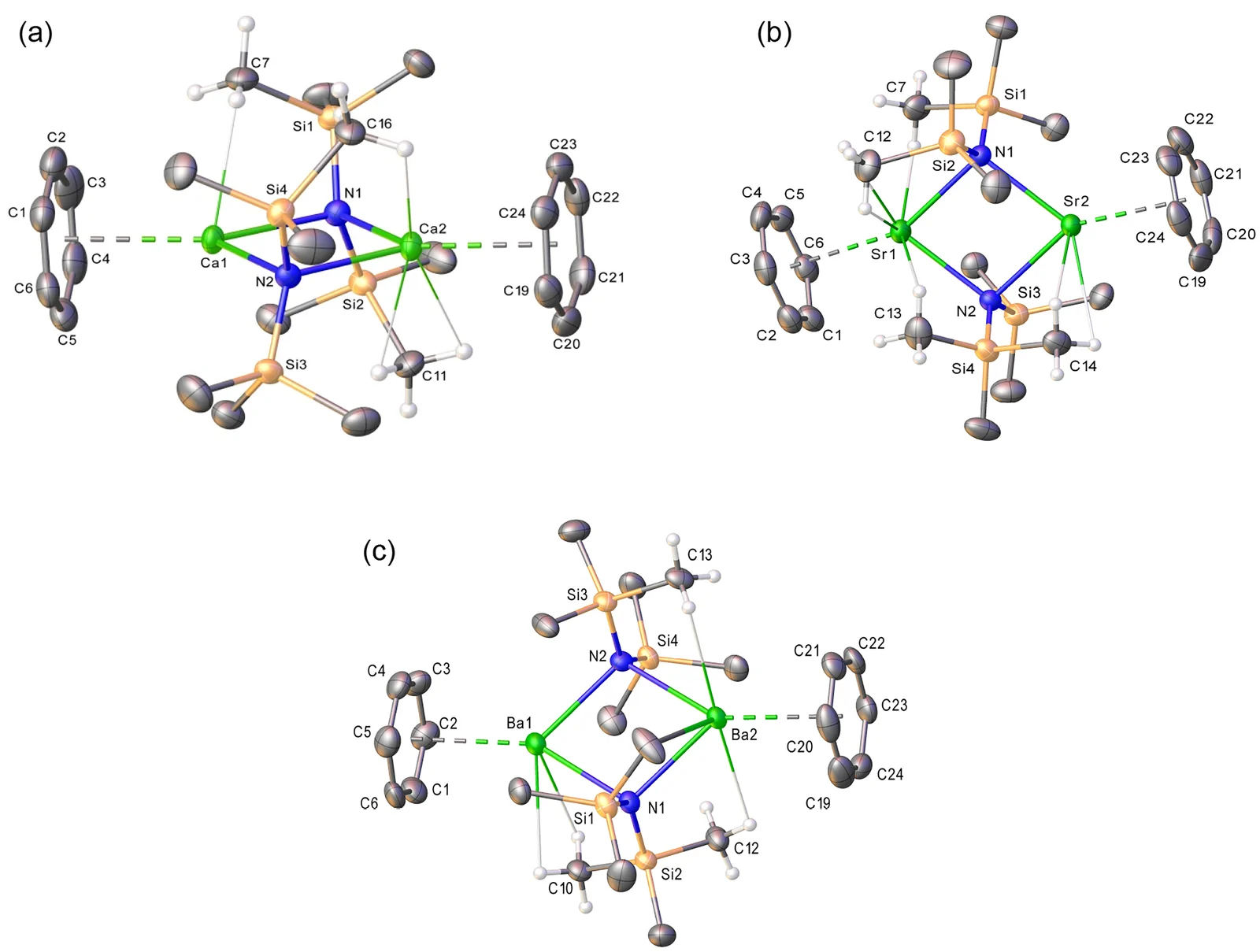
Molecular structures of the dicationic components of (a) compound 1, (b) compound 2 and (c) compound 3 with displacement ellipsoids at 30%. For clarity, all aluminate anions, hydrogen atoms except those attached to selected silylmethyl carbons, and occluded and disordered molecules of benzene solvent (2) are omitted.

**Table 1:** Selected bond lengths (Å) and angles (°) for the dicationic components of compounds 1–3

	1 a	2 b	3 c
Ae1–N1	2.374(7)	2.601(4)	2.704(8)
Ae1–N2	2.386(7)	2.491(3)	2.624(8)
Ae2–N1	2.361(7)	2.520(3)	2.657(8)
Ae2–N2	2.368(7)	2.596(3)	2.755(8)
Ae1–Cent_(C1–C6)_	2.657(4)	2.759(2)	2.880(6)
Ae1–C1	3.024(10)	3.107(5)	3.189(13)
Ae1–C2	3.020(11)	3.116(5)	3.199(14)
Ae1–C3	3.004(10)	3.074(5)	3.206(14)
Ae1–C4	2.954(9)	3.057(5)	3.190(12)
Ae1–C5	2.950(10)	3.086(5)	3.187(14)
Ae1–C6	2.971(9)	3.107(5)	3.212(11)
Ae2–Cent_(C19–C24)_	2.639(4)	2.744(2)	2.949(6)
Ae2–C19	2.966(9)	3.075(5)	3.208(11)
Ae2–C20	2.978(10)	3.076(5)	3.226(11)
Ae2–C21	3.007(10)	3.056(5)	3.256(11)
Ae2–C22	2.973(9)	3.073(5)	3.295(13)
Ae2–C23	2.978(10)	3.059(5)	3.287(15)
Ae2–C24	2.977(9)	3.060(5)	3.246(12)
N1–Ae1–N2	85.4(2)	84.83(11)	83.9(2)
N1–Ae2–N2	86.1(2)	84.36(11)	82.3(2)
Ae1–N1–Ae2	94.5(2)	94.97(11)	97.1(3)
Ae1–N2–Ae2	94.0(2)	95.83(11)	96.6(3)

a Ae = Ca.

b Ae = Sr.

c Ae = Ba.

While no direct benzene-coordinated strontium or barium comparators exist for either compound 2 and 3, the range of Sr–C distances in 2 [2.950(10) to 3.024(1) Å] bear close comparison to the variously substituted monocyclic arenes in the strontium adducts, VII [2.945(3) to 3.117(3) Å]^45^ and VIII^Sr^ [2.997(3) to 3.025(3) Å],^46^ despite the dicationic and monometallic Sr constitutions of these previously reported species. Similarly, the range of Ba–C distances displayed by 3 [3.187(14) to 3.295(13) Å] are broadly commensurate with those of a further hexamethylbenzene adduct of barium isolated as a Al–μ-F–Ba contact ion pair with the [F-Al{OC(CF_3_)_3_}_3_]^−^ anion [3.134(7) to 3.193(7) Å].^46^ Despite these variations across the benzene-to-Ae engagements in 1–3, the resultant benzene C–C bonds within all three compounds are identical within experimental error and display no perceptible elongation in comparison to the crystallographically-determined C–C bond length of unperturbed C_6_H_6_ itself (1.398 Å).^74^

### Mesitylene coordination

Although its *D*_3h_ symmetry ensures the maintenance of the twofold degeneracy of its ground state, the substitution of 1,3,5-trimethylbenzene (mesitylene) results in a raising of the HOMO energy by >0.5 eV in comparison to that of benzene.^48,75,76^ In order to assess the impact of the greater potential π-donor capacity implied by mesitylene's consequent greater susceptibility to electrophilic aromatic substitution, a further series of reactions were performed under identical conditions as employed in the synthesis of 1–3 but with mesitylene replacing the benzene solvent. These reactions again provided biphasic systems redolent of liquid clathrate formation, albeit now comprising a purple solution and a more viscous deep red layer. Despite the intense colour of the reaction solutions, colourless crystals of compounds 4–6, which could again be isolated by mechanical separation, were observed to form when the viscous layer was allowed to stand undisturbed. Attempted characterisation by ^1^H and ^19^F NMR spectroscopy by redissolution of the crystals in *d*_8_-toluene was again largely uninformative and hampered by similar issues of clathrate re-formation and likely solution exchange with the arene solvent. X-ray diffraction analysis of mechanically separated single crystals, however, revealed the formation of a common structural type for each of the three group 2 cations that clearly contrasted with those deduced for compounds 1–3. The single bis(trimethylsilyl)amide ligand retained by each alkali metal is terminally bound such that the group 2 centres now exist as isolated monocations (Fig. 3a–c and Table 2). The otherwise naked [(Me_3_Si)_2_NAe]^+^ cations are each further η^6^-coordinated by two molecules of mesitylene, which again ensure that any direct interaction to the [Al{OC(CF_3_)_3_}_4_]^−^ anions is prevented. In a similar manner to the synthesis of compound 3, the molecule of THF introduced as a component of the barium amide starting material, X, is completely excluded from the resultant cation of 6. As expected, the Ae–N bond lengths in 4–6 [4 2.213(5); 5 2.365(7); 6 2.493(13) Å] are notably foreshortened, not only in comparison to those presented by the bridging interactions of 1–3 (Table 1), but also typical Ae–N distances displayed by similar terminal coordination of hexamethyldisilazide in charge neutral Ae metal complexes (Ae = Ca ≈ 2.30; Sr ≈ 2.45; Ba ≈ 2.6 Å).^77,78^ Consistent with the anticipated enhanced π-donor capacity of the arene rings, the Ca-to-mesitylene-centroid distances of 4 [avg. 2.578 Å] represent a *ca.* 2.6% shortening in comparison to the similarly polyhapto calcium-to-benzene-centroid interactions of 1. In contrast, the comparable metrics across both the cationic strontium cations, 2 and 5, and those of the barium species, 3 and 6, are effectively identical within experimental error, albeit with greater variance across the individual Ae–C interactions (Tables 1 and 2). Although we ascribe no specific relevance to the variations across the six compounds, and have not further investigated their origin, it is plausible that the most compact bis-mesitylene-coordinated species, 4, benefits from further intramolecular C–H dispersive interactions when arrayed around the smallest calcium centre included in this study.^79^

**Fig. 3:**
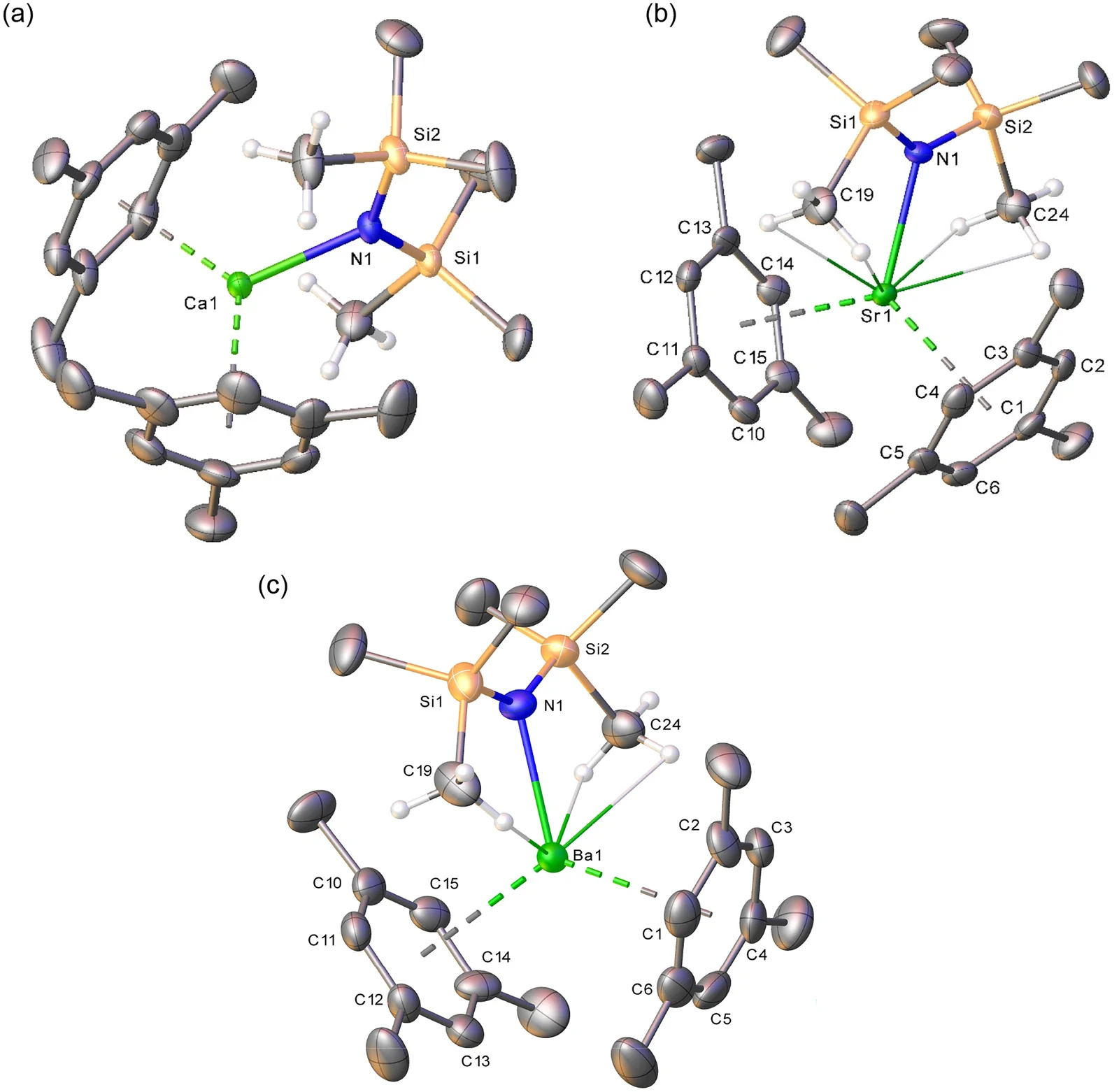
Molecular structures of (a) the Ca1-containing cation of compound 4, (b) the cation of compound 5 and (c) the cation of compound 6 with displacement ellipsoids at 30%. For clarity, all aluminate anions and hydrogen atoms, except those attached to selected silylmethyl carbons, are omitted.

**Table 2:** Selected bond lengths (Å) and angles (°) for the cationic components of compounds 4–6

	4 a^,^^b^	5 c	6 d
Ae1–N1	2.213(5)	2.365(7)	2.493(13)
Ae1–Cent_(C1–C6)_	2.570(10)	2.753(3)	2.919(9)
Ae1–C1	2.956(8)	3.082(6)	3.236(12)
Ae1–C2	2.895(8)	3.044(6)	3.216(12)
Ae1–C3	2.883(7)	3.046(6)	3.213(11)
Ae1–C4	2.893(8)	3.085(6)	3.230(11)
Ae1–C5	2.954(8)	3.123(6)	3.249(12)
Ae1–C6	2.969(8)	3.121(6)	3.252(12)
Ae1–Cent_(C10–C15)_	2.585(7)	2.737(5)	2.901(13)
Ae1–C10	2.894(6)	3.075(9)	3.195(11)
Ae1–C11	2.942(6)	3.099(9)	3.236(11)
Ae1–C12	2.982(9)	3.067(9)	3.26(2)
Ae1–C13	2.976(6)	3.076(8)	3.237(12)
Ae1–C14	2.928(6)	3.051(9)	3.196(12)
Ae1–C15	2.887(6)	3.070(9)	3.175(11)
N1–Ae1–Cent_(C1–C6)_	34.5(3)	32.31(12)	32.8(3)
N1–Ae1–Cent_(C10–C15)_	34.2(3)	35.47(15)	35.8(4)
Cent_(C1–C6)_–Ae1–Cent_(C10–C15)_	126.74(15)	125.19(11)	125.5(3)

a Ae = Ca.

b Ca1-containing moiety only.

c Ae = Sr.

d Ae = Ba.

### Chlorobenzene coordination

The greater π-donor strength implied by the isolation of the bis-arene ligated structures of compounds 4–6 led us to consider the consequences of further modulation of the π-basic nature of the arene donor. Analogous reactions between the trityl aluminate and each of the group 2 bis(trimethylsilylmide) starting materials (IX^Ae^ and X) were, thus, also performed in chlorobenzene, selected as an exemplary haloarene that is less reactive to electrophiles than either of its wholly hydrocarbon-based analogues. Although all three reactions provided similar visual observations and the familiar formation of liquid clathrate systems, no crystalline material could be isolated from the calcium-based reaction of IX^Ca^. In contrast, colourless crystals of compounds 7 and 8 could be mechanically separated from the purple and orange viscous solutions yielded by the respective strontium and barium reagents, IX^Sr^ and X.

While attempted analysis by NMR spectroscopy of the isolated crystals in *d*_8_-toluene was again undiagnostic and most likely resulted in chlorobenzene displacement, the connectivity of the strontium-containing moiety provided by X-ray diffraction analysis of 7 confirmed its formulation as the contact ion pair structure presented in Fig. 4a. Attempts to model disorder of a further charge separated aluminate anion present within the asymmetric unit, however, necessitated the application of ADP and distance restraints resulting in high *R*_1_, w*R*_2_, *R*_int_ and GooF values, such that the bond metrics were deemed unreliable. Although this obviates detailed structural discussion, the asymmetric unit comprises a single non-coordinated aluminate along with a cationic component in which, like compound 2, two strontium centres are connected by a pair of μ-N-bridging bis(trimethyl)silylamide anions. Further reminiscent of 2, Sr1 engages *via* its η^6^-interaction with a single molecule of the arene solvent. The implied less coordinating nature of chlorobenzene to strontium is apparent from the coordination sphere of Sr2, which is not completed by a further molecule of the arene solvent. Rather, its coordinative unsaturation is satisfied by threefold Sr⋯F–C close contacts with a perfluorinated alkoxide substituent of the Al1-containing aluminate. This mode of engagement of strontium is, thus, strongly reminiscent of one of the aluminate anions previously observed by Krossing and co-workers in Sr[Al{OC(CF_3_)_3_}_4_]_2_.^45^

**Fig. 4:**
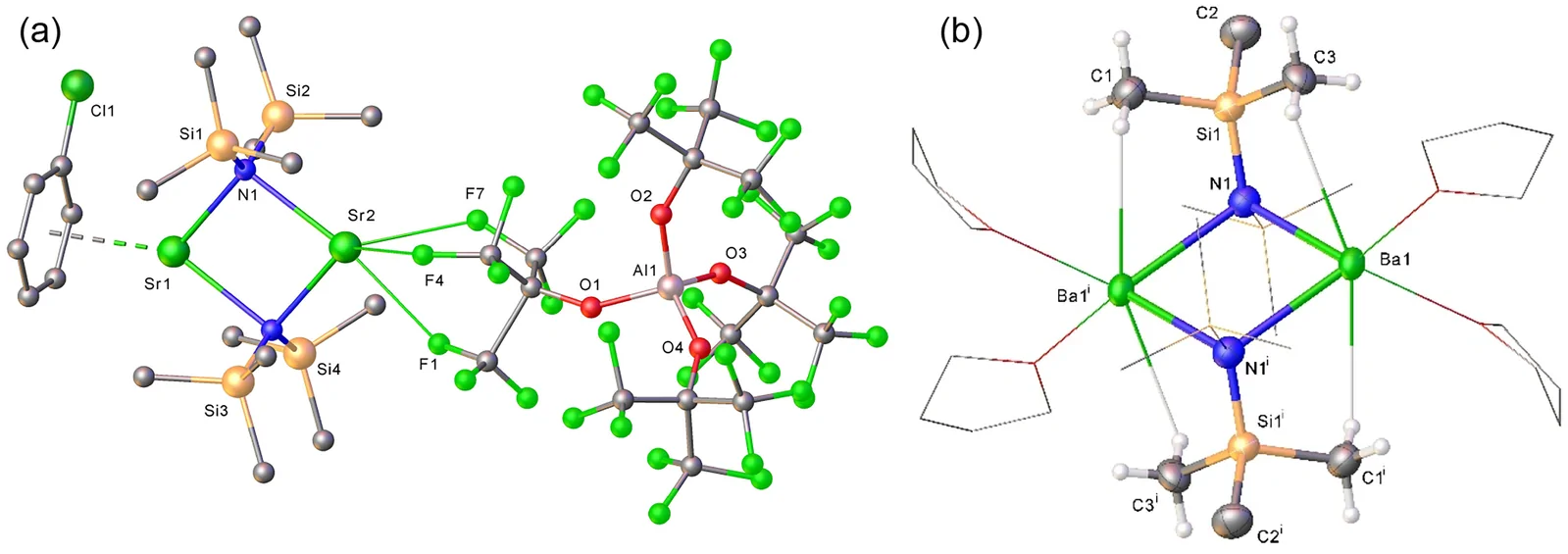
(a) Ball and stick depiction of the molecular connectivity of the cationic component of 7. Hydrogen atoms and the non-coordination aluminate anion have been removed for clarity. (b) The dicationic component of compound 8 with displacement ellipsoids at 30%. For clarity, all aluminate anions, hydrogen atoms except those attached to selected silylmethyl carbons are omitted and THF molecules and the Si2-containing trimethylsilyl substituents are drawn as wireframe. Selected bond lengths (Å) and angles (°) (8): Ba1–N1 2.737(6), Ba1–N1^1^ 2.766(6), Ba1–O1 2.695(6), Ba1–O2 2.700(5), N1–Ba1–N1^1^ 84.03(19), O1–Ba1–O2 90.83(19), O1–Ba1–N1 128.25(19), O1–Ba1–N1^1^, 117.76(19), O2–Ba1–N1^1^ 123.76(18), O2–Ba1–N1 116.30(18). Symmetry operations to generate equivalent atoms; ^1^1 − *x*, 2 − *y*, 1 − *z*; ^2^2 − *x*, −*y*, 1 − *z*.

The lower Ae^2+^ binding affinity of C_6_H_5_Cl was underscored by the X-ray diffraction analysis of compound 8 (Fig. 4b). Although a further dimeric bis-μ-N-bridged dication, the symmetry-related barium centres of compound 8 are devoid of any arene interactions and their coordination spheres are completed by two molecules of THF. Consideration of the 1 : 1 THF to barium ratio provided by the amide starting material (X), indicates that the stoichiometry of 8 cannot be representative of the constitution of the orange liquid clathrate layer, which, in all likelihood, comprises Ba^2+^ cations encapsulated by a variable number of chlorobenzene molecules. Although no further species could be isolated from the viscous orange phase formed in the reaction, this observation serves to emphasise that a modicum of caution should always be applied if interpreting the bulk formulation of such systems based on single crystal X-ray data.

### Quantum chemical calculations

The contrasting formation of the cations from benzene and mesitylene was investigated computationally. This was achieved through interrogation of the electronic structures of not only the cationic components of the dinuclear benzene (denoted as 1^Ben^–3^Ben^) and monometallic bis-mesitylene (4^Mes^–6^Mes^) solvates but also the corresponding di- and mononuclear cations in which benzene was, respectively, replaced by mesitylene (1^Mes^–3^Mes^) and mesitylene by benzene (4^Ben^–6^Ben^, Fig. 5). Density Functional Theory calculations (DFT, see SI for data, methodology = BP86-D3^BJ^(CPCM)/ZORA-def2-TZVPP/SARC//BP86/def2-SVP (B)) showed that in each case the formation of the alternate structures is marginally endergonic, with relative free energies ranging from +1.3 to +5.0 kcal mol^−1^. While these data do not account for the consequences of crystal packing in the ionic structures of 1–6, this energetic similarity indicates that, in all likelihood, the gelatinous liquid clathrate layers observed prior to crystallisation comprise an equilibrating mixture of cationic forms, which, furthermore, present variable and dynamic secondary interactions to the aromatic host solvent.^80^ As expected, the separation between the arene and metal cation centre increases down group 2 (2.68–3.13 Å), albeit these calculated distances were consistently shorter than those observed experimentally in 1–6. The natural charges associated with the Ae atoms were also calculated to display an incremental increase with atomic mass (*q*_Ae_ = +1.73–1.83), and the charges borne by the individual group 2 ions were in every case slightly higher for the respective mesitylene structures (∼0.01) in comparison to their benzene-coordinated analogues.

**Fig. 5:**
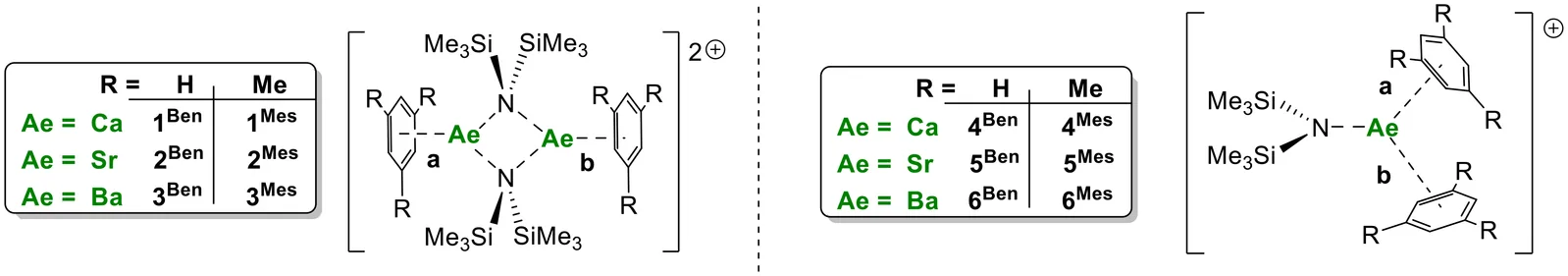
Benzene- (1^Ben^–6^Ben^) and mesitylene (1^Mes^–6^Mes^) complexes included in the computational study.

Irrespective of arene identity, the HOMOs of all the dicationic structures (1^Ben^–3^Ben^ and 1^Mes^–3^Mes^) are concentrated on the nitrogen atoms of the bis(trimethylsilyl)amide ligands, whilst the LUMOs are concentrated on the π-systems of the arene groups (see Fig. S19–S24). NBO analysis demonstrates a significant reduction in s-orbital contribution to the lone vacancy NBO for the alkali metal for each of these dicationic complexes in comparison to each of their monometallic comparators (4^Ben^–6^Ben^ and 4^Mes^–6^Mes^) (Table S11). In the bimetallic dications, both 1^Ben^–3^Ben^ and 1^Mes^–3^Mes^, the relevant valence s-orbital contributes almost exclusively (96.0–97.2%). However, in the monometallic structures, 4^Ben^–6^Ben^ and 4^Mes^–6^Mes^, the s-orbital contribution ranges from 52.9–80.1%, with the theoretical benzene variants (4^Ben^–6^Ben^) exhibiting the smaller s orbital contribution by *ca*. 15, 10 and 9% when descending group 2. The remaining AO contributions to the Ae lone vacancy NBO are predominately from the d-orbitals, specifically the valence d_*x*^2^−*y*^2^_ and d_*z*^2^_ orbitals, with the d_*x*^2^−*y*^2^_ contribution roughly four times larger than the d_*z*^2^_ component (see Table S11). No notable donor–acceptor interactions (Δ*E*^(2)^) were observed between the arene rings and the lone vacancy NBO of the Ae cation above 1 kcal mol^−1^.

Energy decomposition analysis (EDA) of the Ae-cation-to-benzene interaction in 1^Ben^–3^Ben^, showed that the total interaction decreases down group 2, (Δ*E*_int_; 1^Ben^ = −34.8, 2^Ben^ = −31.5 and 3^Ben^ = −27.8 kcal mol^−1^). When replacing those benzene rings with mesitylene, the total interaction increases for each alkali metal by ∼10 kcal mol^−1^ (1^Mes^ = −44.2, 2^Mes^ = −41.7, 3^Mes^ = −36.9 kcal mol^−1^). The largest contribution to the total interaction comes from the Δ*E*_orb_ parameter. An almost identical trend persists for the monometallic mesitylene geometries 4^Mes^–6^Mes^; 4^Mes^ = −38.7, 5^Mes^ = −35.3, 6^Mes^ = −30.9 kcal mol^−1^, whilst the benzene variants saw total interactions of 4^Ben^ = −29.3, 5^Ben^ = −26.3, 6^Ben^ = −23.4 kcal mol^−1^, an overall reduction of 9 kcal mol^−1^. Although Δ*E*_orb_ is still the larger component, it is almost equal in size now to Δ*E*_Elstat_. This follows the established Ae interaction trend of Ca > Sr > Ba and indicates that mesitylene-to-Ae cation interactions are stronger than for benzene.

## Conclusions

This study further confirms the significant affinity of heavier alkaline earth cations for the polyhapto sequestration of arene solvents when crystallised in combination with the weakly coordinating aluminate anion, [Al{OC(CF_3_)_3_}_4_]^−^. Although the identification of bimetallic, mono-coordinated structures from benzene and bis-ligated monocations from mesitylene infers a greater binding affinity for the more electron rich π system, in no case could any significant perturbation of the arene structure be identified. DFT calculations on the isolated cationic structures are consistent with this viewpoint, confirming the energetic similarity of both structural types and describing a decreasing affinity for arene binding in the order Ca > Sr > Ba. Although we do not ascribe any specific chemical consequence to the observation, our calculations also invoke a notable level of *n*d-orbital participation in arene binding by [(Me_3_Si)_2_NAe(η^6^-arene)]^+^, but which is all but negated by silazide bridging.

## Conflicts of interest

There are no conflicts to declare.

## Supplementary Material

DT-OLF-D6DT02345B-s001

## Data Availability

Supplementary information (SI): experimental details, NMR spectra, X-ray crystallography, computational details, and atomic coordinates for the optimized geometries of the compounds. See DOI: https://doi.org/10.1039/d6dt02345b. CCDC 2591358–2591364 for 1–6 and 8 contain the supplementary crystallographic data for this paper.^81*a*–*g*^
