# Supplementary material for: Arene binding by silazido heavier alkaline earth cations
Source: Dalton Trans. 2026 Oct 29. Online ahead of print. doi: 10.1039/d6dt02345b (PMC13728943; doi:10.1039/d6dt02345b)
Supplement: Supplementary file 1 [file DT-OLF-D6DT02345B-s001.pdf]

Supplementary Information (SI) for Dalton Transactions.
This journal is © The Royal Society of Chemistry

Supplementary Information for

# Arene Binding by Silazido Heavier Alkaline Earth Cations

Oliver Jarvis, Jacob T. Guntley, Michael S. Hill,* Mary F. Mahon, Kyle G. Pearce and Claire L. McMullin*

*Department of Chemistry, University of Bath, Claverton Down, Bath, BA2 7AY, UK*

**Synthetic Details**

**General Considerations** S2

**Synthesis of [{Ca(HMDS)($C_6D_6$)}$_2$][Al(OC($CF_3$)$_3$)$_4$]$_2$ (1)** S2

**Synthesis of [{Sr(HMDS)($C_6D_6$)}$_2$][Al(OC($CF_3$)$_3$)$_4$]$_2$ (2)** S2

**Synthesis of [{Ba(HMDS)(C6H6)}$_2$][Al(OC(CF3)$_3$)$_4$]2 (3)** S2

**Synthesis of [Ca(HMDS)($C_6H_3${$CH_3$}$_3$)$_2$][Al(OC($CF_3$)$_3$)$_4$] (4)** S2

**Synthesis of [Sr(HMDS)($C_6H_3${$CH_3$}$_3$)$_2$][Al(OC($CF_3$)$_3$)$_4$] (5)** S2

**Synthesis of [Ba(HMDS)($C_6H_3${$CH_3$}$_3$)$_2$][Al(OC($CF_3$)$_3$)$_4$] (6)** S3

**Synthesis of [PhClSr(HMDS)$_2$Sr…Al(OC($CF_3$)$_3$)$_4$][Al(OC($CF_3$)$_3$)$_4$]$_2$ (7)** S3

**Synthesis of [{Ba(HMDS)(THF)$_2$}$_2$][Al(OC($CF_3$)$_3$)$_4$]$_2$ (8)** S3

**NMR Spectra**

**Figure S1-S2:** NMR Spectra for **[{Ca(HMDS)($C_6D_6$)}$_2$][Al(OC($CF_3$)$_3$)$_4$]$_2$ (1)** S4

**Figure S3-S4:** NMR Spectra for **[{Sr(HMDS)($C_6D_6$)}$_2$][Al(OC($CF_3$)$_3$)$_4$]$_2$ (2)** S5

**Figure S5-S6:** NMR Spectra for **[{Ba(HMDS)($C_6D_6$)}$_2$][Al(OC($CF_3$)$_3$)$_4$]$_2$ (3)** S6

**Figure S7-S8:** NMR Spectra for **[Ca(HMDS)($C_6H_2${$CH_3$}$_3$)$_2$][Al(OC($CF_3$)$_3$)$_4$] (4)** S7

**Figure S9-S10:** NMR Spectra for **[Sr(HMDS)($C_6H_2${$CH_3$}$_3$)$_2$][Al(OC($CF_3$)$_3$)$_4$] (5)** S8

**Figure S11-S12:** NMR Spectra for **[Ba(HMDS)($C_6H_2${$CH_3$}$_3$)$_2$][Al(OC($CF_3$)$_3$)$_4$] (6)** S9

**Figure S13-S14:** NMR Spectra for **[PhClSr(HMDS)$_2$SrAl(OC($CF_3$)$_3$)$_4$][Al(OC($CF_3$)$_3$)$_4$]$_2$ (7)** S10

**Figure S15-S16:** NMR Spectrum for **[{Ba(HMDS)(THF)$_2$}$_2$][Al(OC($CF_3$)$_3$)$_4$]$_2$ (8)** S11

**Crystallographic Details** S12

**Computational Details** S17

**References** S36

## Synthetic Details

### General Considerations

All manipulations were carried out using standard Schlenk line and glovebox techniques under an inert atmosphere of argon. NMR experiments were conducted in J-Young's NMR tubes and prepared in a glovebox. NMR spectra were recorded on a Bruker BioSpin GmbH spectrometer operating at 400.13 MHz ($^1$H) and 376.48 MHz ($^{19}$F). Elemental analyses were performed at Elemental Microanalysis Ltd., Okehampton, Devon, UK or by the Elemental Analysis Services Team at London Metropolitan University. Solvents were dried by passage through a commercially available solvent purification system and stored under argon in ampoules over 4 Å molecular sieves. $C_6D_6$ and $C_7D_8$ were purchased from Merck, dried over potassium, distilled and stored over molecular sieves. $Ae(N\{SiMe_3\}_2)_2$ (Ae = Ca, Sr), [1,2] $[Ba\{N(SiMe_3)_2\}_2](THF)]_2$[3] and $[Ph_3C][Al\{OC(CF_3)_3\}_4]$ were prepared according to literature procedures.[4]

### Synthesis of $[\{Ca(HMDS)(C_6D_6)\}_2][Al(OC(CF_3)_3)_4]_2$ (1)

$[Ca\{N(SiMe_3)_2\}_2]$ (15 mg, 0.04 mmol) and $[Ph_3C][Al(OC(CF_3)_3)_4]$ (50.3 mg, 0.04 mmol) were added to a J. Young's NMR tube and dissolved in $d_6$-benzene forming two immiscible layers. The solution turned red and red crystals were immediately formed. The crystals were decanted away with the solution from the gooey bottom layer and was left to evaporate. $^1$H NMR ($C_6D_6$): δ = 0.10 (s, NSi<u>Me</u>$_3$). $^{19}$F NMR ($C_6D_6$) δ = –74.81.

### Synthesis of $[\{Sr(HMDS)(C_6D_6)\}_2][Al(OC(CF_3)_3)_4]_2$ (2)

$[Sr\{N(SiMe_3)_2\}_2]$ (9.8 mg, 0.02 mmol) and $[Ph_3C][Al(OC(CF_3)_3)_4]$ (29 mg, 0.02 mmol) were introduced into a J. Young's NMR tube and dissolved in $d_6$-benzene forming dark red crystals under a red solution. $^1$H NMR ($C_6D_6$): δ =0.10 (s, NSi<u>Me</u>$_3$). $^{19}$F NMR ($C_6D_6$) δ = –74.85.

### Synthesis of $[\{Ba(HMDS)(C_6H_6)\}_2][Al(OC(CF_3)_3)_4]_2$ (3)

$[Ba\{N(SiMe_3)_2\}_2](THF)]_2$ (11 mg, 0.02 mmol) and $[Ph_3C][Al(OC(CF_3)_3)_4]$ (29 mg, 0.02 mmol) were introduced into a J. Young's NMR tube and dissolved in $d_8$-toluene forming dark red crystals under a pink solution. $^1$H NMR ($C_7D_8$): δ = 0.09 (s, NSi<u>Me</u>$_3$). $^{19}$F NMR ($C_7D_8$) δ = –74.86.

### Synthesis of $[Ca(HMDS)(C_6H_3\{CH_3\}_3)_2][Al(OC(CF_3)_3)_4]$ (4)

$[Ca\{N(SiMe_3)_2\}_2]$ (15 mg, 0.04 mmol) and $[Ph_3C][Al(OC(CF_3)_3)_4]$ (50.3 mg, 0.02 mmol) were introduced into a J. Young's NMR tube and dissolved in mesitylene forming a dark purple solution with a gloopy red layer immediately formed alongside colourless crystals. Some crystals were removed from the NMR tube, dried under vacuum and dissolved in toluene for spectroscopic analysis. The crystals were analysed by X-ray diffraction. $^1$H NMR ($C_7D_8$): δ = 0.11 (s, NSi<u>Me</u>$_3$). $^{19}$F NMR ($C_7D_8$) δ = –75.48.

**Synthesis of $[Sr(HMDS)(C_6H_3\{CH_3\}_3)_2][Al(OC(CF_3)_3)_4]$ (5)**

$[Sr\{N(SiMe_3)_2\}_2]$ (9.8 mg, 0.02 mmol) and $[Ph_3C][Al(OC(CF_3)_3)_4]$ (29 mg, 0.02 mmol) were introduced into a J. Young's NMR tube and dissolved in mesitylene forming a dark purple solution with a gloopy red layer immediately formed alongside colourless crystals. The crystals were dissolved in toluene and the crystals were analysed by X-ray diffraction. $^1H$ NMR ($C_7D_8$): δ = 0.09 (s, NSi$\underline{Me}_3$). $^{19}F$ NMR ($C_7D_8$) δ = –75.2.

**Synthesis of $[Ba(HMDS)(C_6H_3\{CH_3\}_3)_2][Al(OC(CF_3)_3)_4]$ (6)**

$[Ba\{N(SiMe_3)_2\}_2](THF)$ (11 mg, 0.02 mmol) and $[Ph_3C][Al(OC(CF_3)_3)_4]$ (29 mg, 0.02 mmol) were introduced into a J. Young's NMR tube and dissolved in mesitylene forming a dark purple solution with a gloopy red layer immediately formed alongside colourless crystals. The crystals were removed and left to slowly evaporate any excess mesitylene. They were then dissolved in toluene for spectroscopic analysis and the crystals were also analysed by X-ray diffraction. $^1H$ NMR ($C_7D_8$): δ = 0.11 (s, NSi$\underline{Me}_3$). $^{19}F$ NMR ($C_7D_8$) δ = –74.85.

**Synthesis of $[PhClSr(HMDS)_2Sr\ldots Al(OC(CF_3)_3)_4][Al(OC(CF_3)_3)_4]_2$ (7)**

$[Sr\{N(SiMe_3)_2\}_2]$ (144.6 mg, 0.35 mmol) and $[Ph_3C][Al(OC(CF_3)_3)_4]$ (428.5 mg, 0.35 mmol) were introduced into a vial and chlorobenzene was added. An immediate purple colouration was observed and colourless crystals were formed. The solution and gooey-bottom layer was decanted away from the crystals and they were analysed by X-ray diffraction. $^1H$ NMR ($C_7D_8$): δ = 0.08 (NSi$\underline{Me}_3$). $^{19}F$ NMR ($C_7D_8$) δ = –74.82.

**Synthesis of $[\{Ba(HMDS)(THF)_2\}_2][Al(OC(CF_3)_3)_4]_2$ (8)**

$[Ba\{N(SiMe_3)_2\}_2](THF)$ (44 mg, 0.08 mmol) and $[Ph_3C][Al(OC(CF_3)_3)_4]$ (100.6 mg, 0.08 mmol) were introduced into a vial and chlorobenzene was added. An immediate orange colouration was observed and colourless crystals were formed. The solution and gooey-bottom layer was decanted away from the crystals and they were analysed by X-ray diffraction. $^1H$ NMR ($C_7D_8$): δ = 0.10 (NSi$\underline{Me}_3$). $^{19}F$ NMR ($C_7D_8$) δ = –74.83.

## NMR Spectra

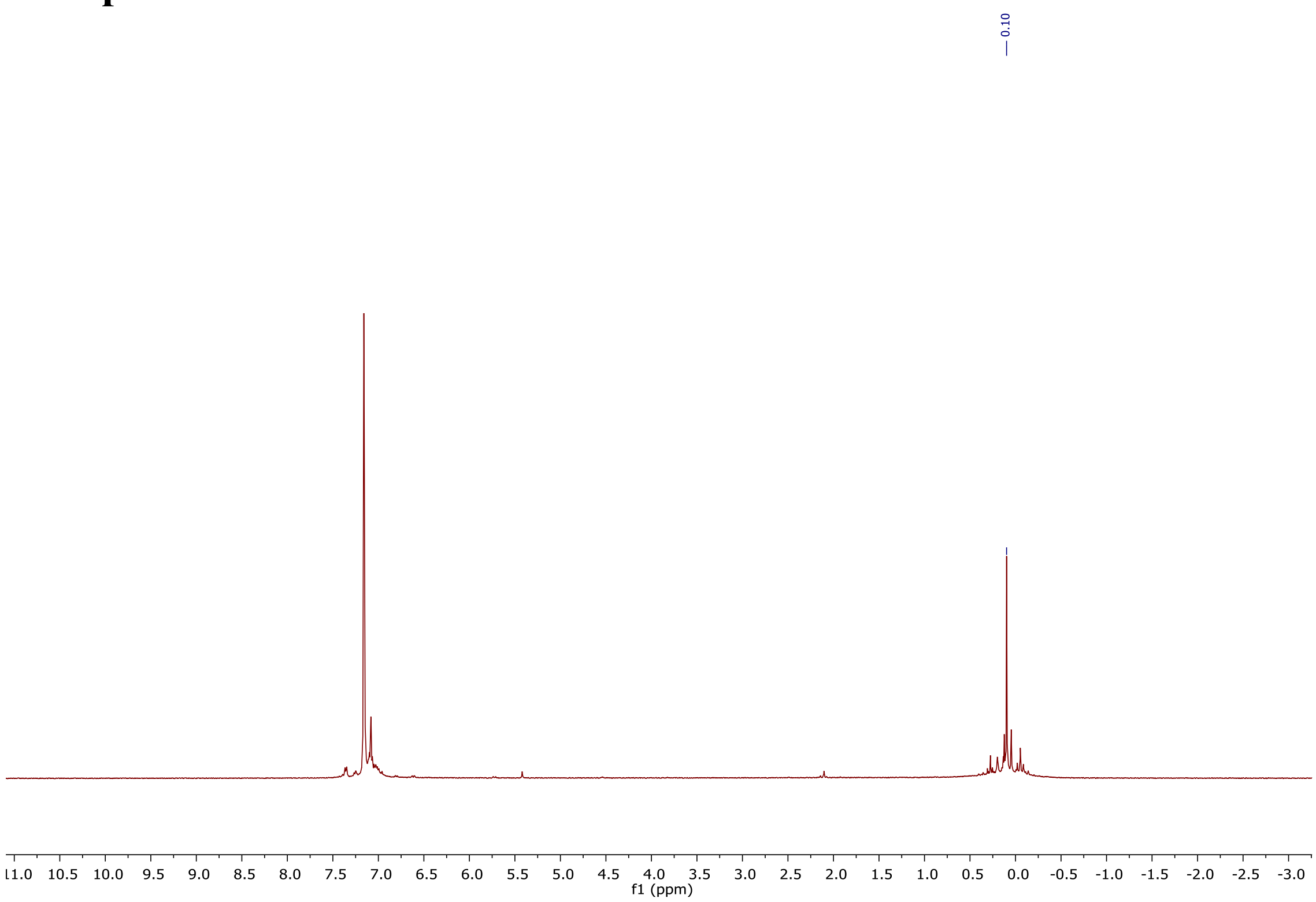


***Figure S1.*** *$^{1}$H NMR Spectrum ($C_6D_6$, 298 K, 400.13 MHz) for* ***[{Ca(HMDS)($C_6D_6$)}$_2$][Al(OC($CF_3$)$_3$)$_4$]$_2$ (1).***

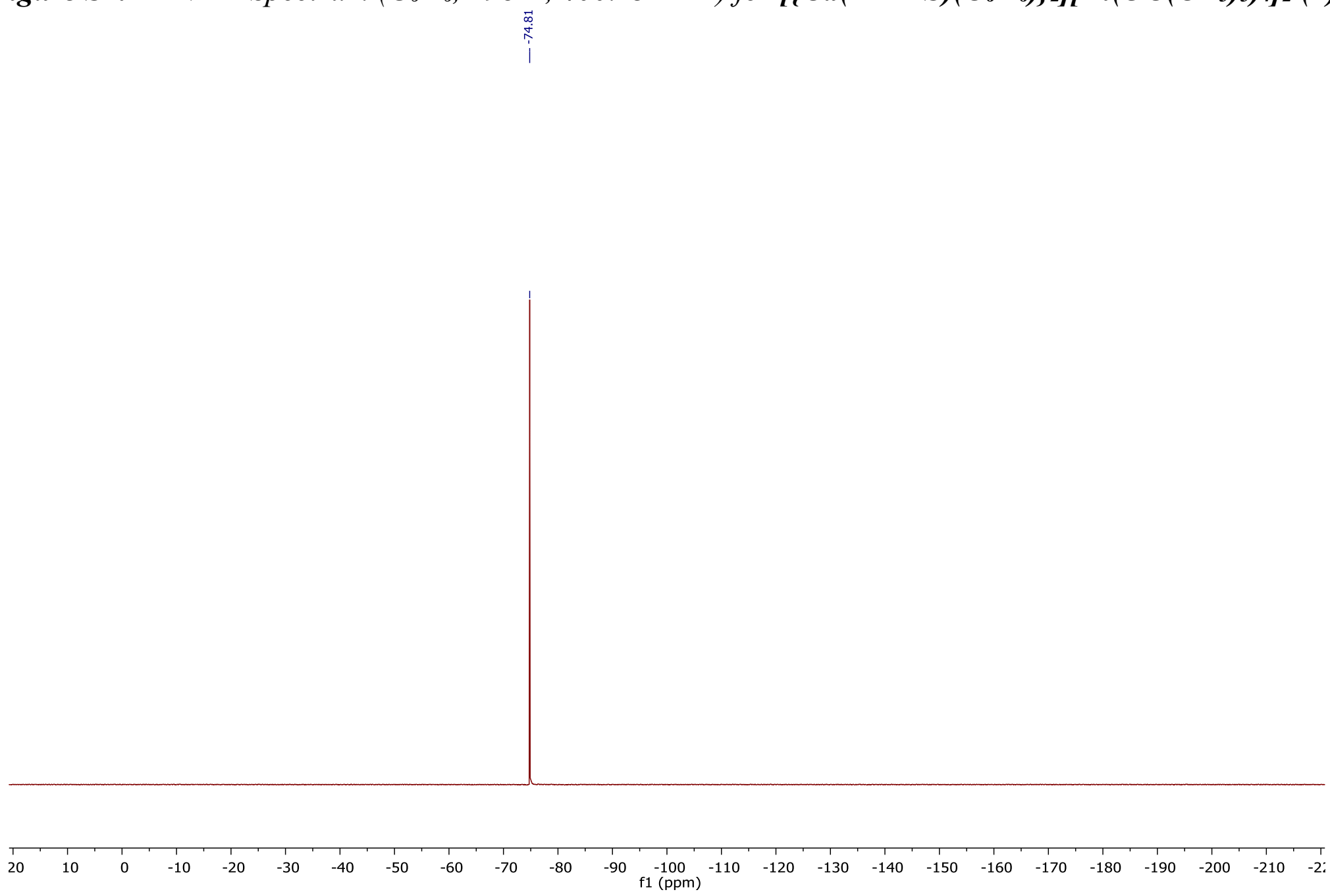


***Figure S2.*** *$^{19}$F NMR Spectrum ($C_6D_6$, 298 K, 376.48 MHz) for* ***[{Ca(HMDS)($C_6D_6$)}$_2$][Al(OC($CF_3$)$_3$)$_4$]$_2$ (1).***

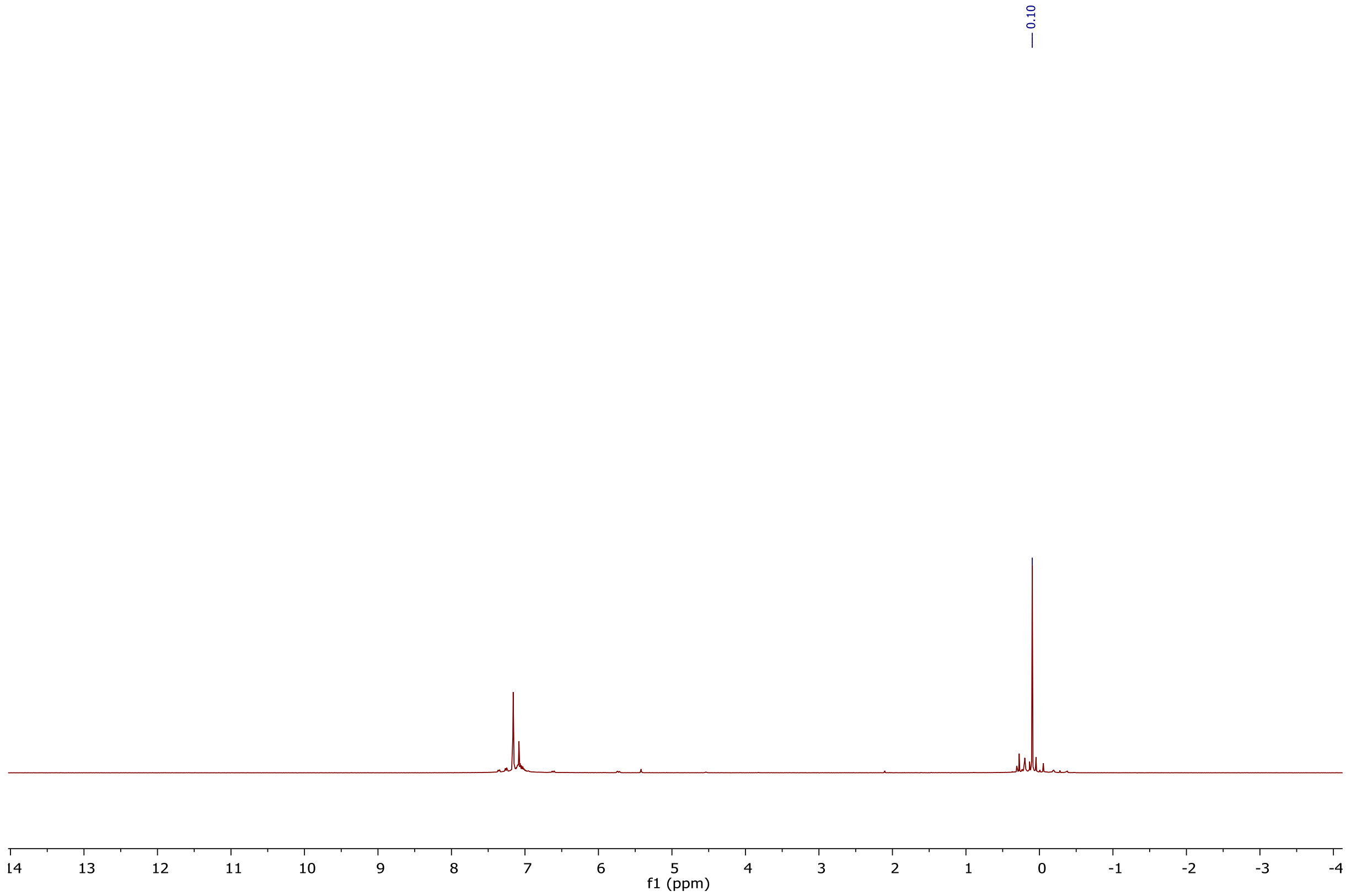


***Figure S3.*** *$^{1}$H NMR Spectrum ($C_6D_6$, 298 K, 400.13 MHz) for* ***$[\{Sr(HMDS)(C_6D_6)\}_2][Al(OC(CF_3)_3)_4]_2$ (2).***

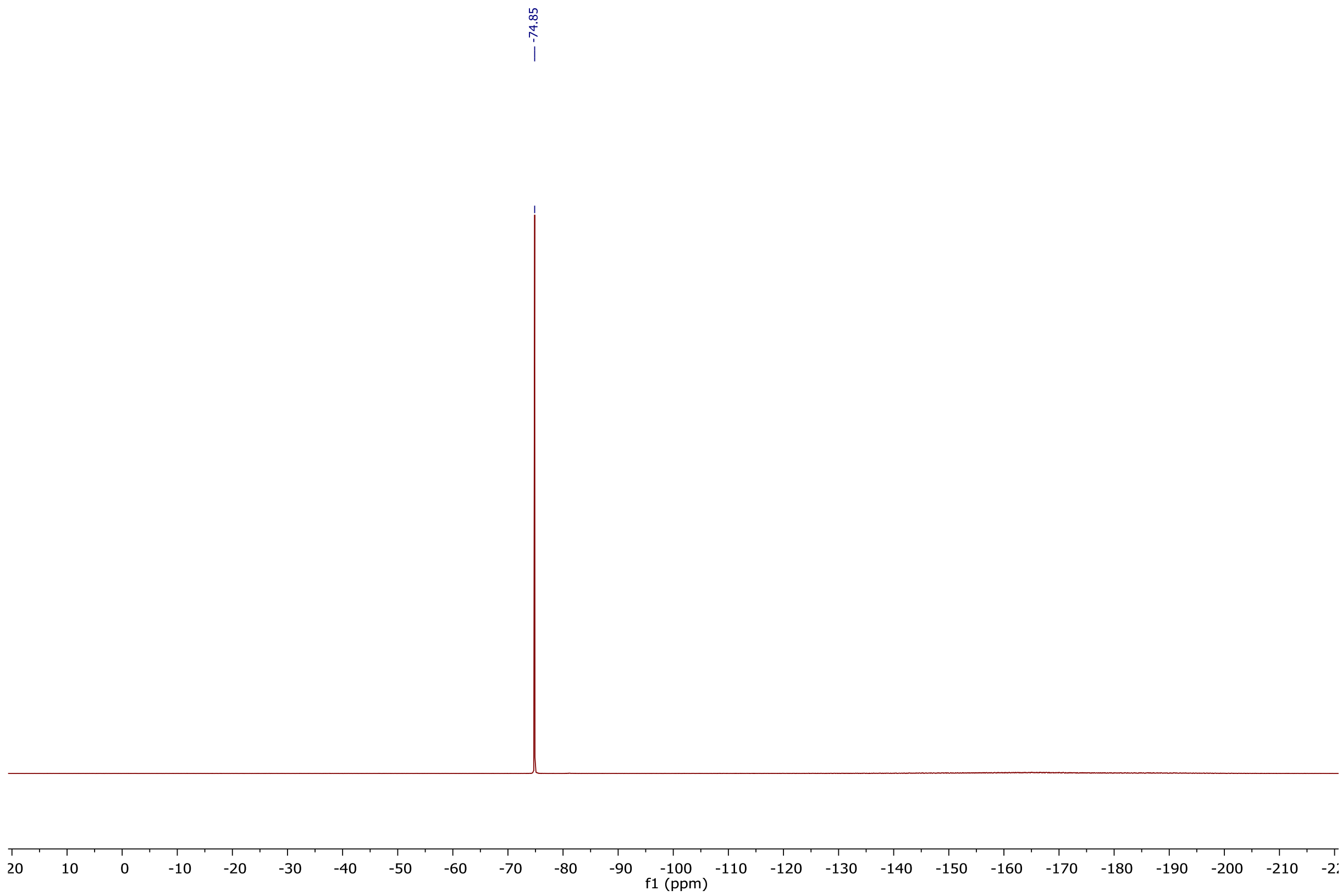


***Figure S4.*** *$^{19}$F NMR Spectrum ($C_6D_6$, 298 K, 376.48 MHz) for* ***$[\{Sr(HMDS)(C_6D_6)\}_2][Al(OC(CF_3)_3)_4]_2$ (2).***

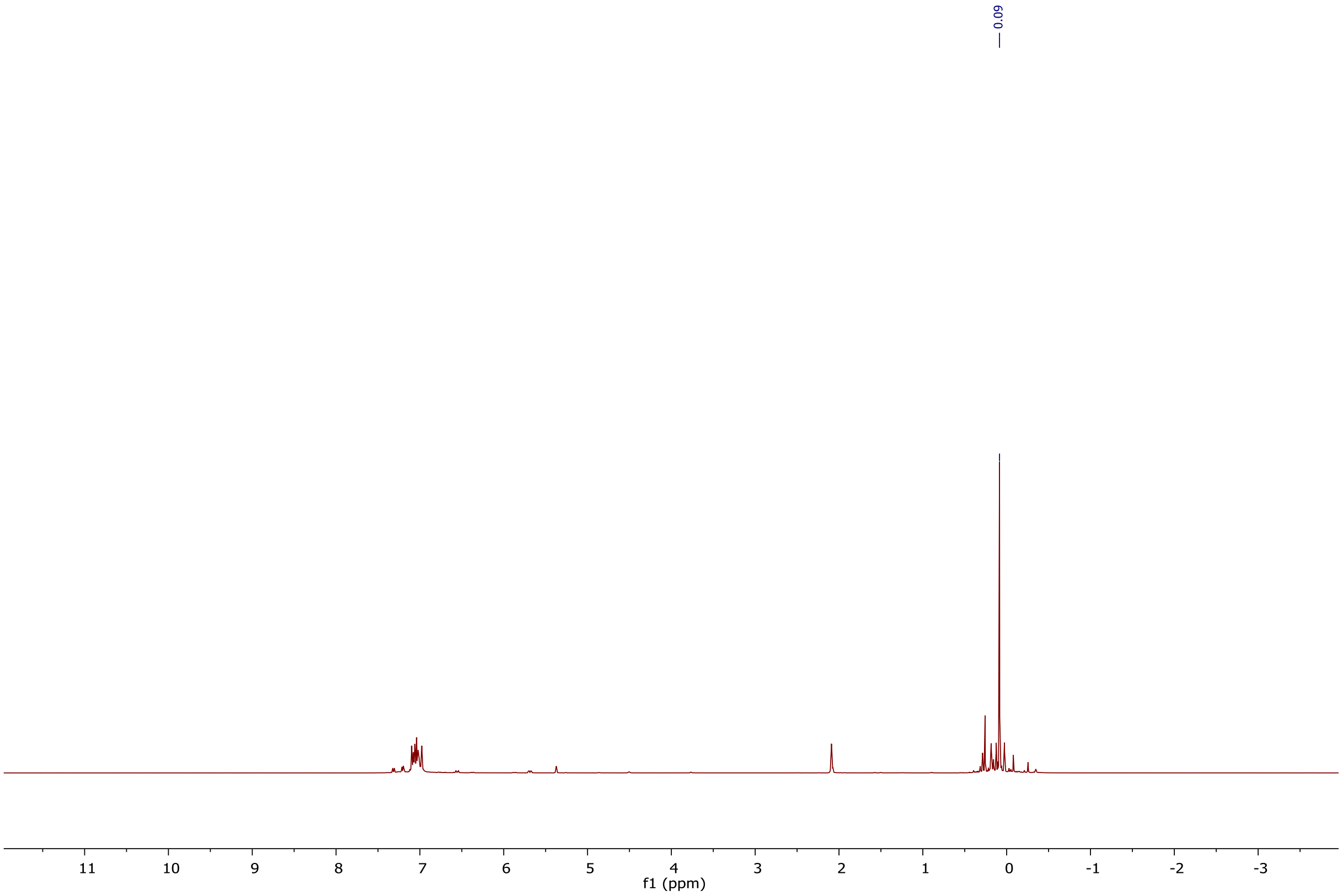


***Figure S5.*** *$^{1}$H NMR Spectrum ($C_7D_8$, 298 K, 400.13 MHz) for* ***[{Ba(HMDS)($C_6D_6$)}$_2$][Al(OC($CF_3$)$_3$)$_4$]$_2$ (3).***

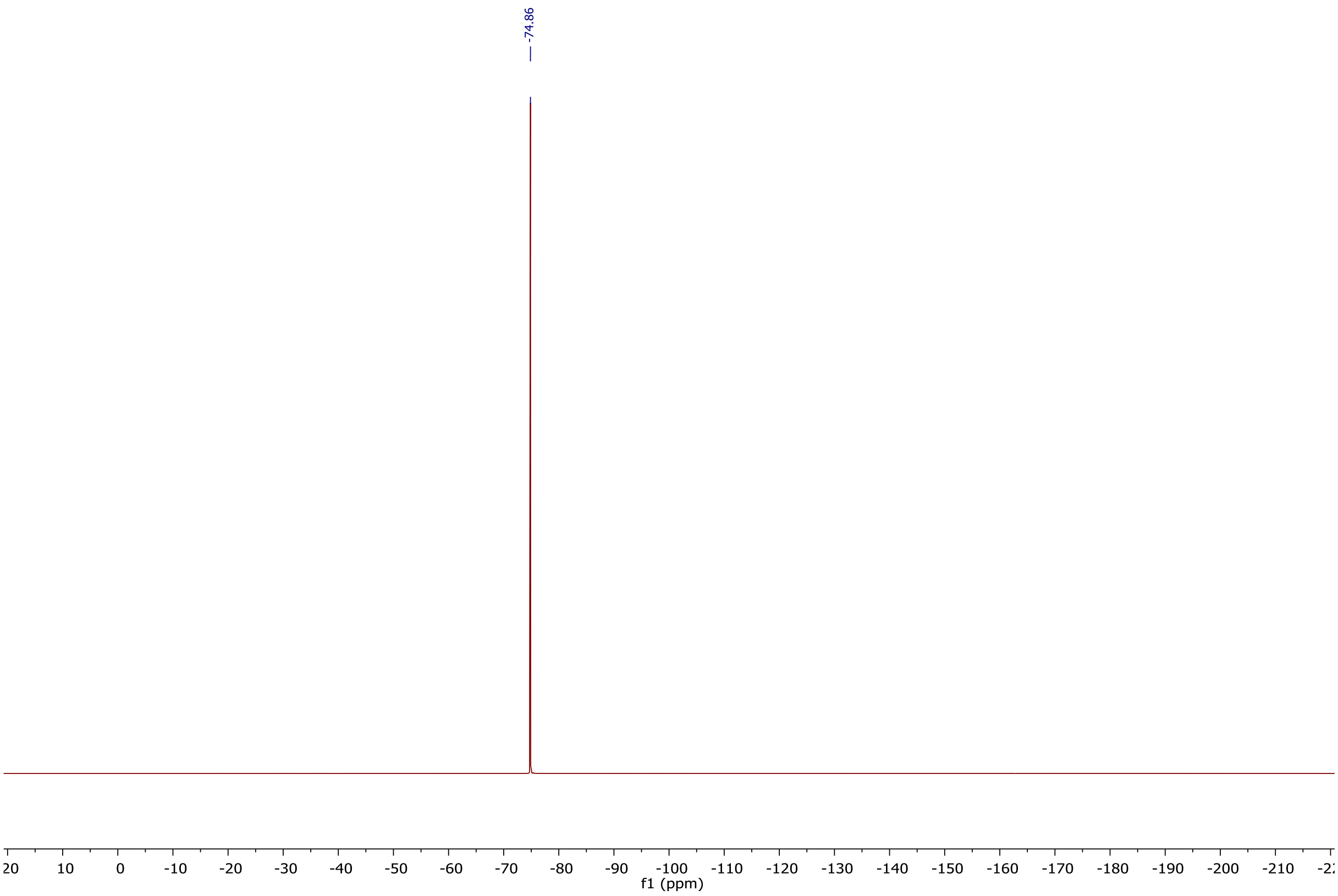


***Figure S6.*** *$^{19}$F NMR Spectrum ($C_7D_8$, 298 K, 376.48 MHz) for* ***[{Ba(HMDS)($C_6D_6$)}$_2$][Al(OC($CF_3$)$_3$)$_4$]$_2$ (3).***

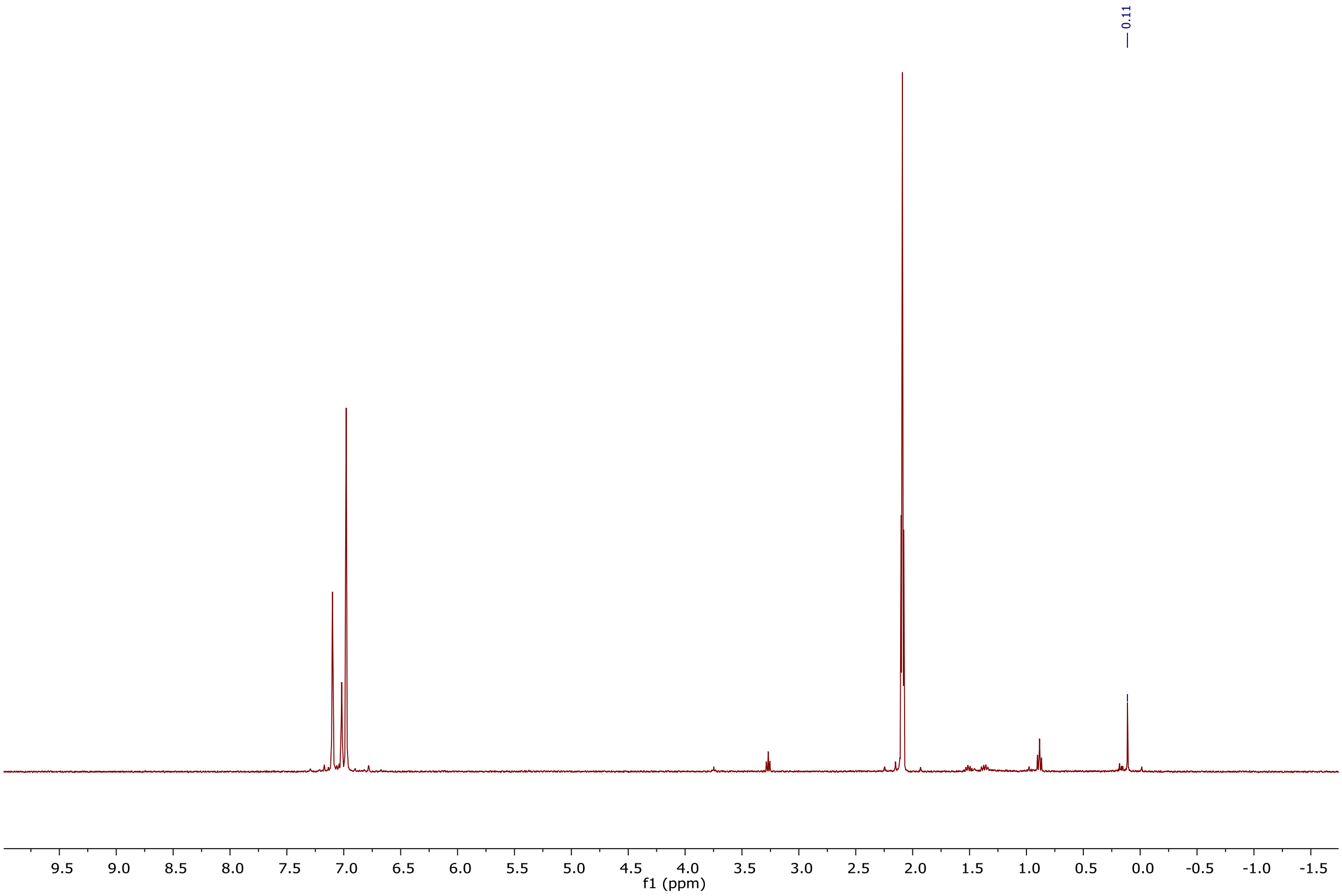


***Figure S7.*** *$^{1}$H NMR Spectrum ($C_7D_8$, 298 K, 400.13 MHz) for* ***[Ca(HMDS)($C_6H_3\{CH_3\}_3$)$_2$][Al(OC($CF_3$)$_3$)$_4$] (4).***

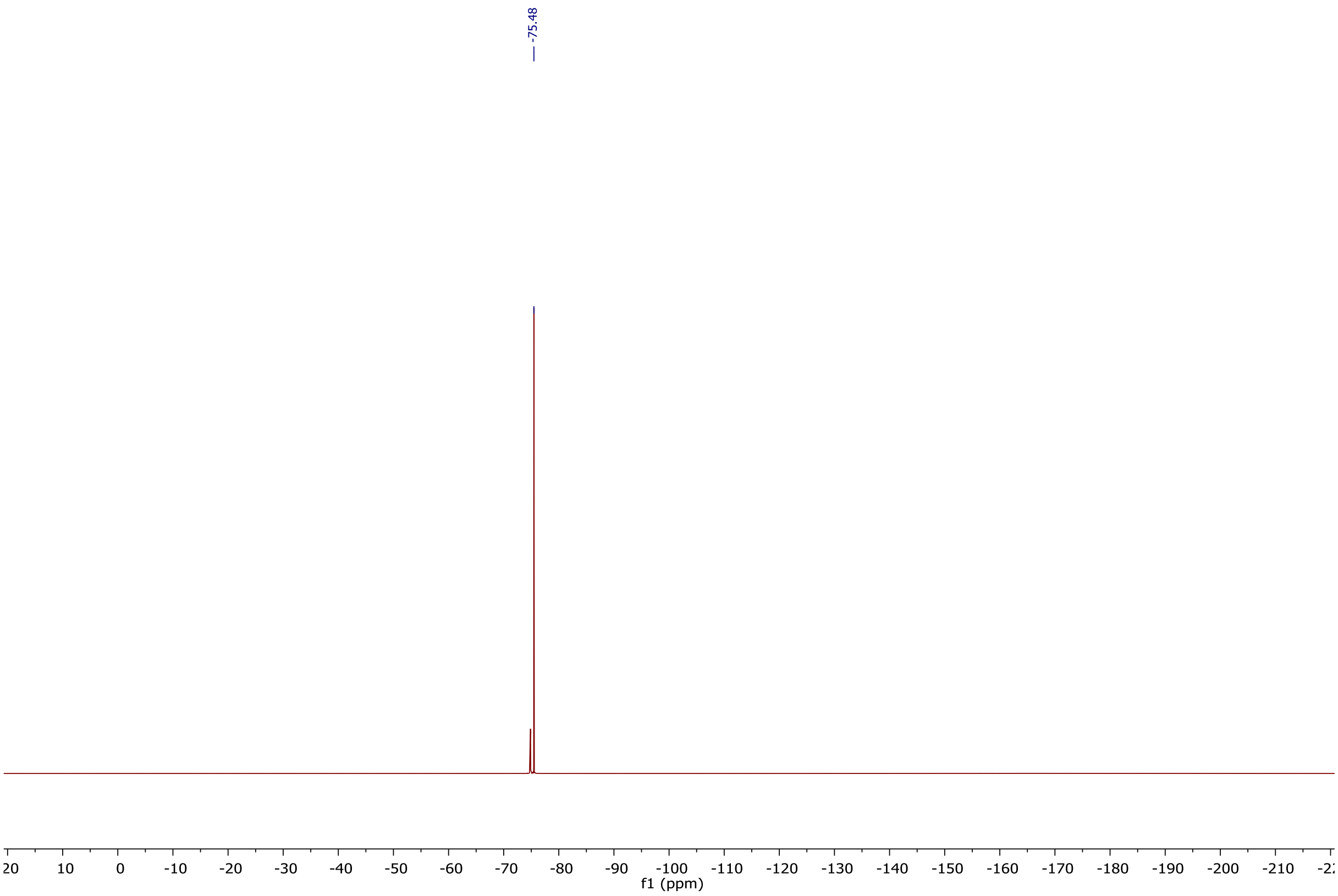


***Figure S8.*** *$^{19}$F NMR Spectrum ($C_7D_8$, 298 K, 376.48 MHz) for* ***[Ca(HMDS)($C_6H_3\{CH_3\}_3$)$_2$][Al(OC($CF_3$)$_3$)$_4$] (4).***

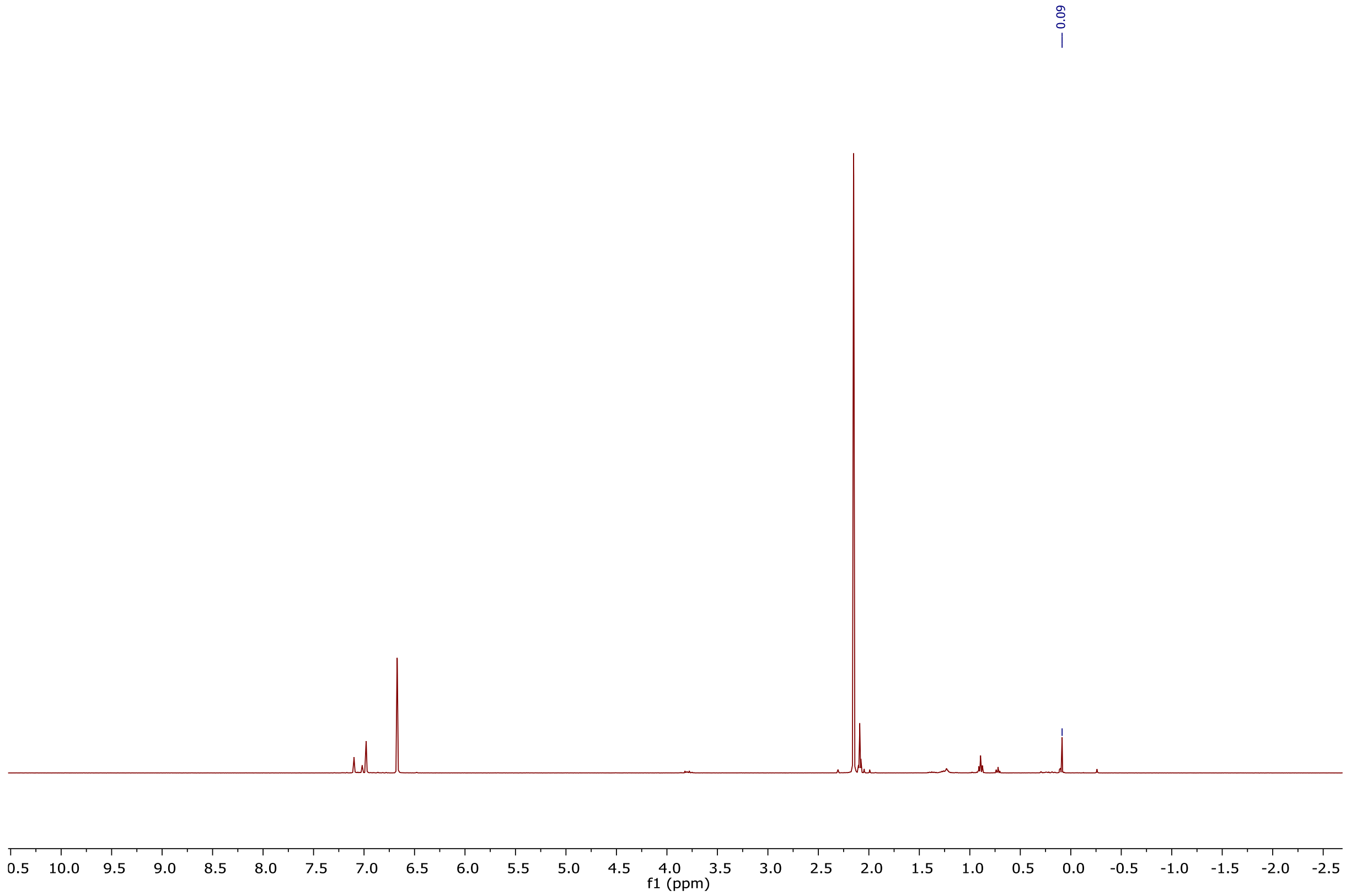


***Figure S9.*** *$^{1}$H NMR Spectrum ($C_7D_8$, 298 K, 400.13 MHz) for* ***[Sr(HMDS)($C_6H_3\{CH_3\}_3$)$_2$][Al(OC($CF_3$)$_3$)$_4$] (5).***

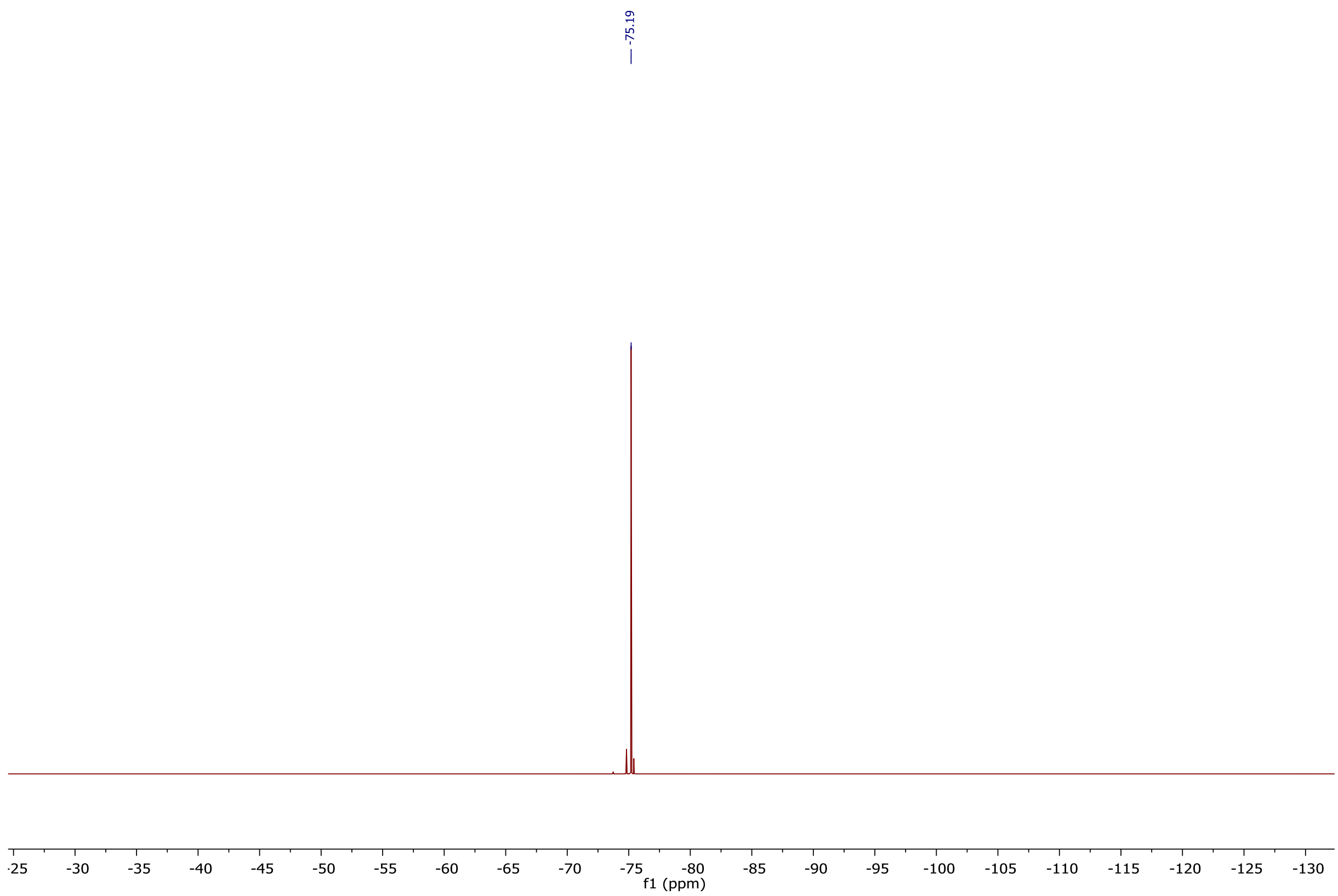


***Figure S10.*** *$^{19}$F NMR Spectrum ($C_7D_8$, 298 K, 376.48 MHz) for* ***[Sr(HMDS)($C_6H_3\{CH_3\}_3$)$_2$][Al(OC($CF_3$)$_3$)$_4$] (5).***

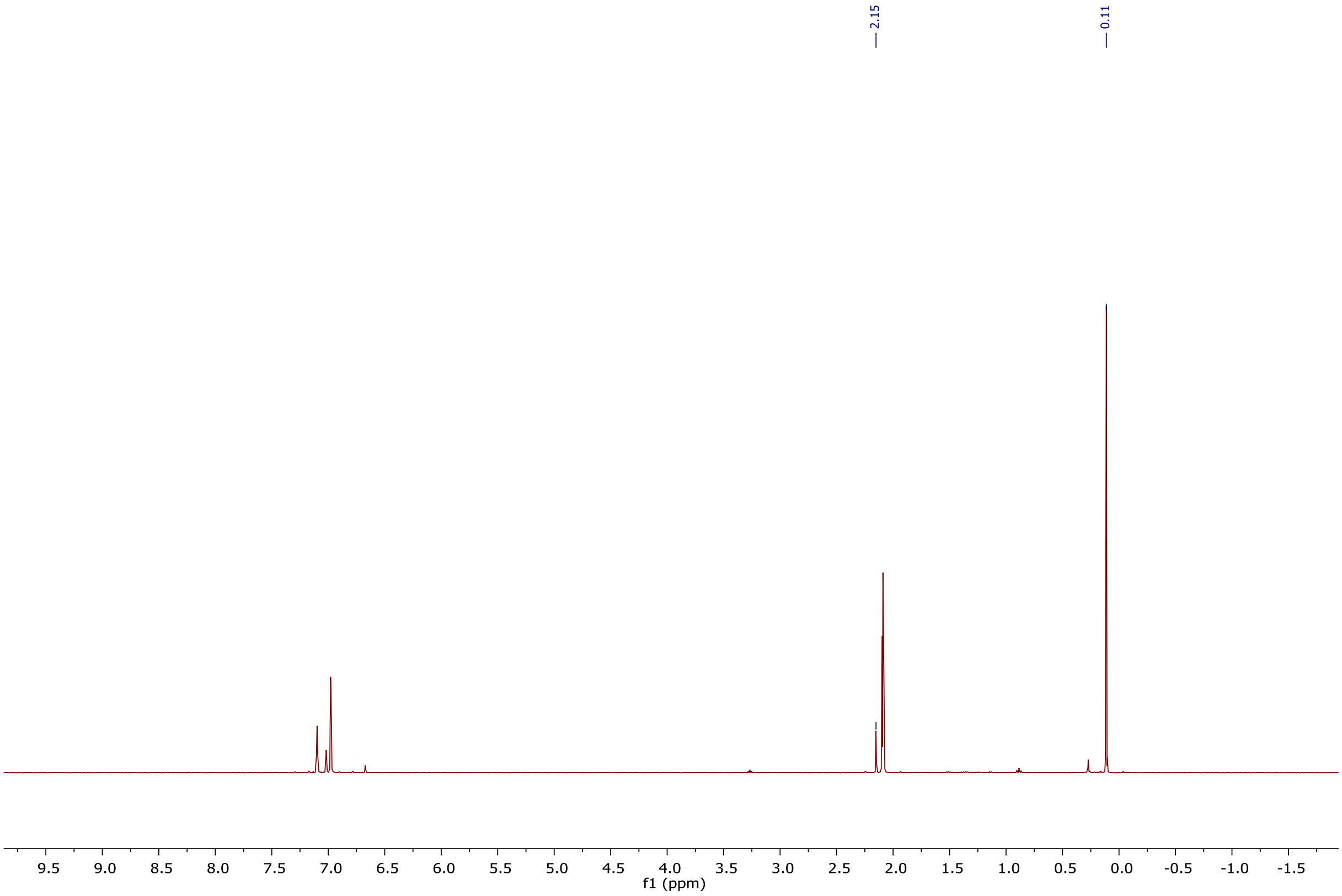


***Figure S11.*** *$^{1}$H NMR Spectrum ($C_7D_8$, 298 K, 400.13 MHz) for* ***[Ba(HMDS)($C_6H_3\{CH_3\}_3$)$_2$][Al(OC($CF_3$)$_3$)$_4$] (6).***

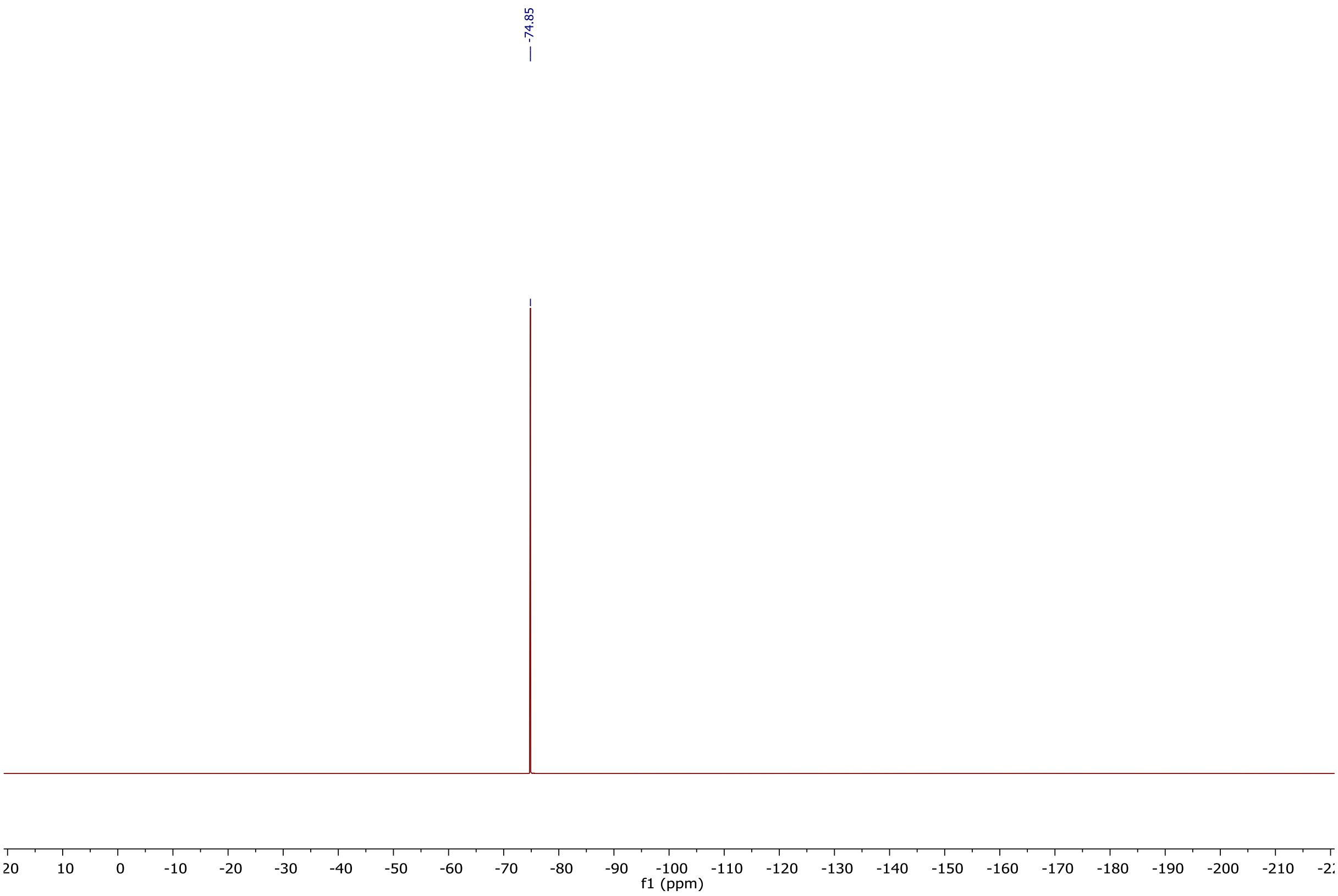


***Figure S12.*** *$^{19}$F NMR Spectrum ($C_7D_8$, 298 K, 376.48 MHz) for* ***[Ba(HMDS)($C_6H_3\{CH_3\}_3$)$_2$][Al(OC($CF_3$)$_3$)$_4$] (6).***

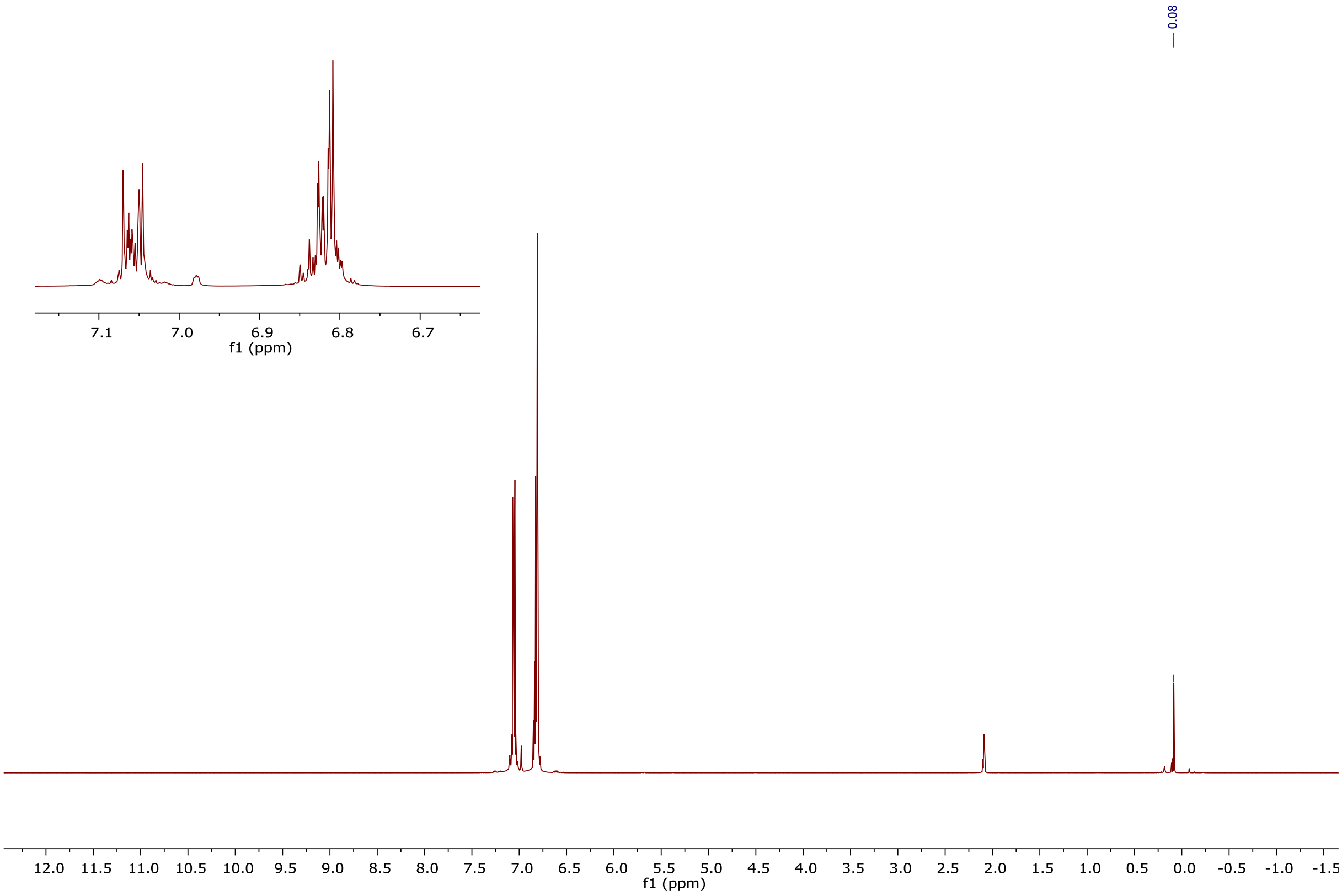


***Figure S13***. [1]*H NMR Spectrum ($C_7D_8$, 298 K, 400.13 MHz) for* ***[PhClSr(HMDS)$_2$Sr…Al(OC(CF$_3$)$_3$)$_4$][Al(OC(CF$_3$)$_3$)$_4$]$_2$ (7).***

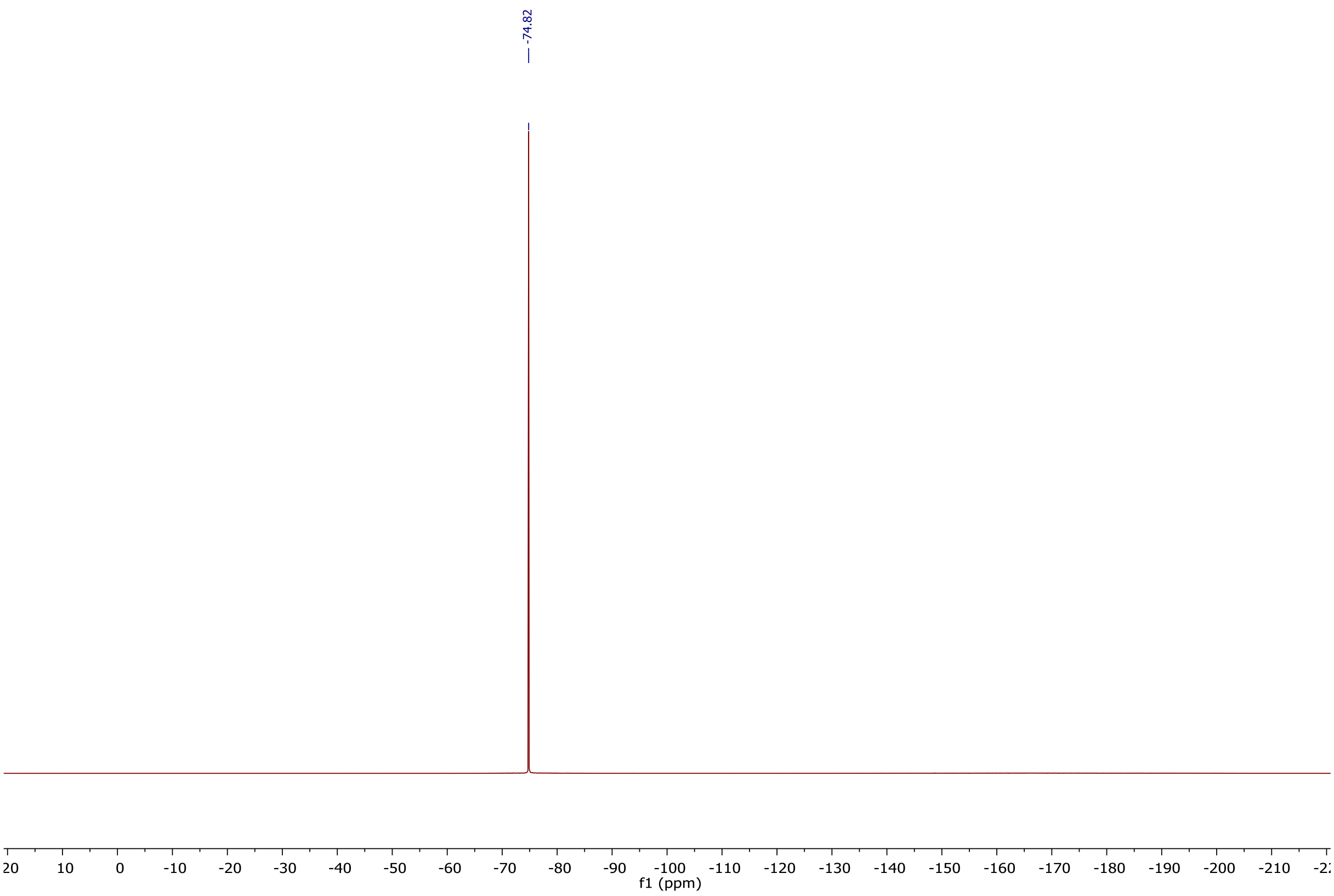


***Figure S14.*** [19]*F NMR Spectrum ($C_7D_8$, 298 K, 376.48 MHz) for* ***[PhClSr(HMDS)$_2$Sr…Al(OC(CF$_3$)$_3$)$_4$][Al(OC(CF$_3$)$_3$)$_4$]$_2$ (7).***

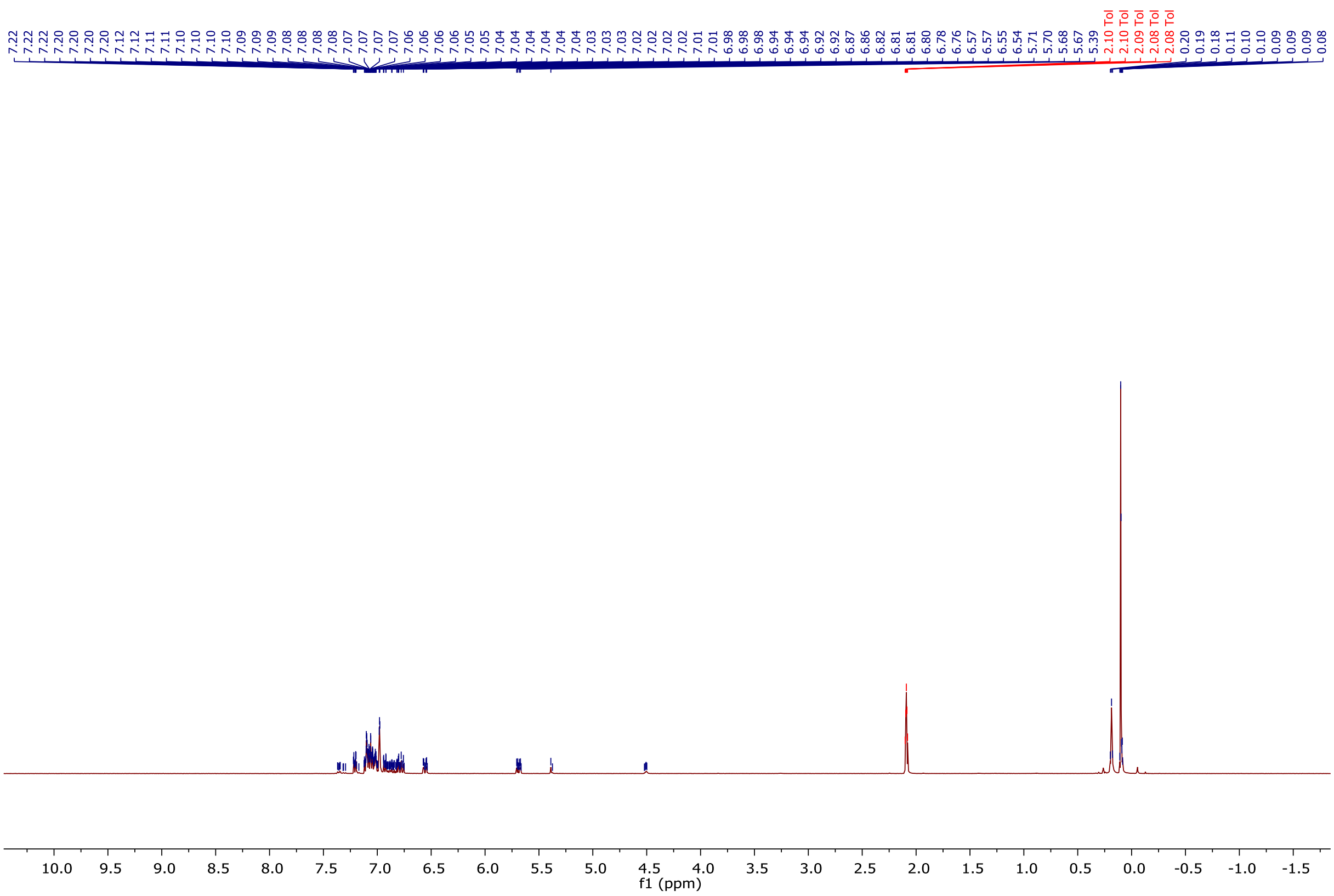


***Figure S15.*** *$^{1}$H NMR Spectrum ($C_7D_8$, 298 K, 400.13 MHz) for* ***[{Ba(HMDS)(THF)$_2$}$_2$][Al(OC(CF$_3$)$_3$)$_4$]$_2$ (8).***

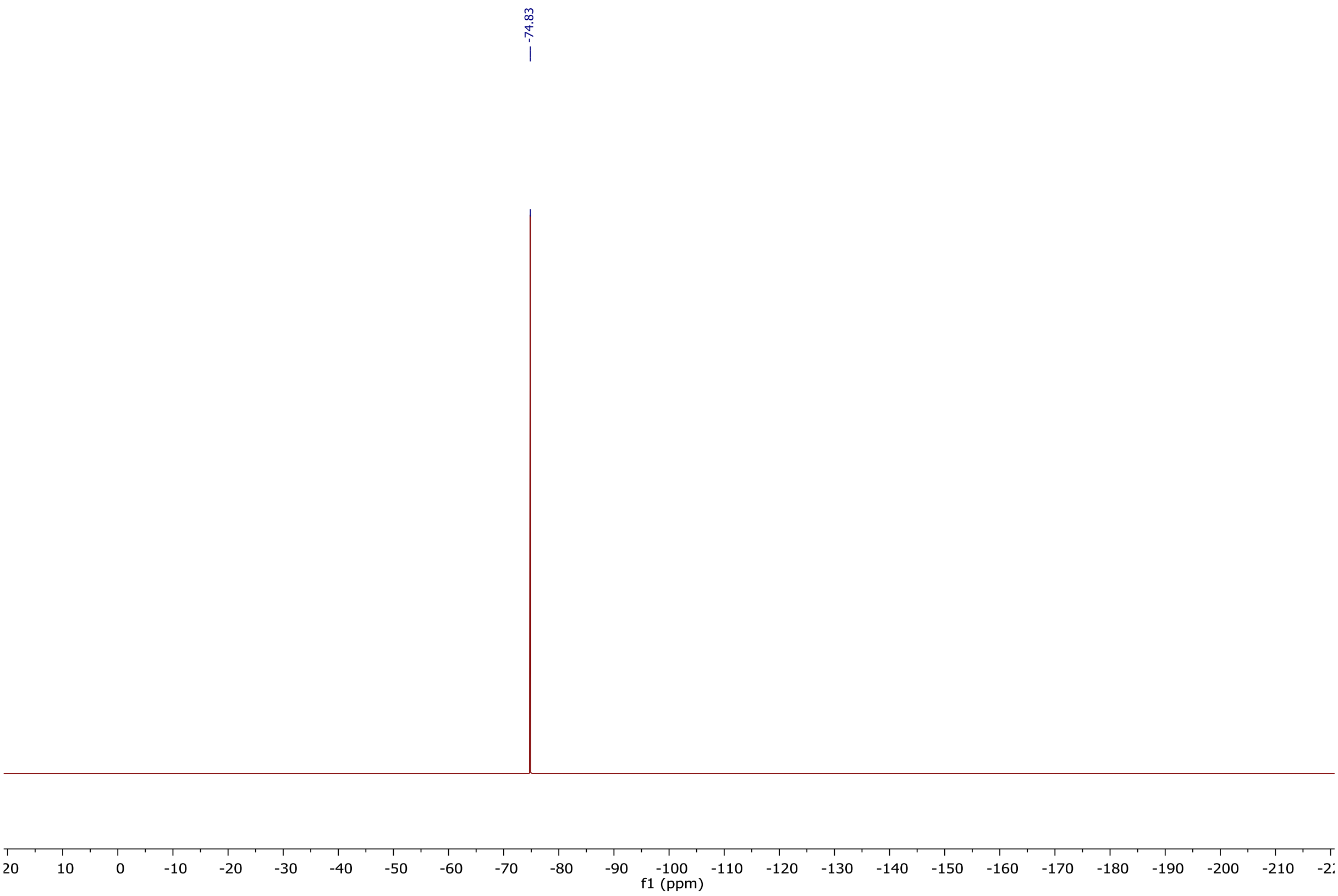


***Figure S16****. $^{19}$F NMR Spectrum ($C_7D_8$, 298 K, 376.48 MHz) for* ***[{Ba(HMDS)(THF)$_2$}$_2$][Al(OC(CF$_3$)$_3$)$_4$]$_2$ (8).***

**Crystallographic Details**

Single Crystal X-ray diffraction data for compound **1** were collected on an Agilent Xcalibur diffractometer using Mo-Kα radiation (0.71073 Å), while compounds **2**, **3**, **4**, **5** and **6** were collected on an Agilent SuperNova EosS2 diffractometer using Cu-Kα (1.54184 Å) radiation. In contrast, compounds **7** and **8** were collected on XtaLAB Synergy, Dualflex, HyPix-Arc 100 diffractometer using Cu-Kα radiation (1.54184 Å). In each case, the crystals were maintained at 150 K during data collection. Using Olex2,[5] the structures were solved with the olex2.solve[6] structure solution program or ShelXT and refined with the ShelXL[7] refinement package using Least−Squares minimisation.

The asymmetric unit in **1** comprises one (ordered) dication and two (very) disordered Krossing anions. Luckily for crystallographers, the FragmentDB plugin for Olex2, which is a GUI-specific implementation of the invaluable DSR refinement package by Kratzert et al.,[8] is available to assist in modelling the -$OC(CF_3)_3$ groups in these weakly coordinating species. In this structure, each of the disordered dendrite components was modelled as a separate residue, with only residue 13 being ordered. Residues 1 and 2 were subject to 65:35 disorder, while residue-pairs 3/4 and 5/6 exhibited 55:45 splits. Pairs 7/8, 9/10 and 11/12 were modelled to take account of 80:20 disorder and, finally, residue-pair 14/15 had site occupancies of 55:45. Distance and ADP restraints were employed, in the disordered region to assist convergence and the large number of restraints pertain only to the modelling of the anions. Although some of the errors pertaining to these restraints exceed the target sigma values, overall, this converged model provides unambiguous characterisation of this salt in the context of an X-ray experiment. Finally, although data were collected to quite a high resolution, they were truncated to a dmax of 0.77Å during integration, as there was a substantial fall-off in diffraction ability of the sample at higher Bragg angles. This is highly likely to be a symptom of the extensive anion-disorder present.

One dication, two Krossing anions, two full molecules of benzene and two benzene halves are manifest in the asymmetric unit in the structure of **2**. The latter fragments were seen to be located proximate to crystallographic inversion centres which serve to complete both molecules. The benzene molecule based on C57 was treated for 65:35 disorder and both constituents were refined as rigid hexagons and with inclusion of ADP restraints, to assist convergence. Remarkably, the anions were ordered, with the only hint of any disorder pertaining to the {$OC(CF_3)_3$} moiety based on O6. The higher than desirable R(int) for the data reflects that this sample was carrying a passenger/twin. However, integration to take account of twinning followed by refinement illustrated that the diffraction from the minor component was in the region of 4%. Moreover, integration as a twin served to substantially degrade the data as a whole. Hence, the model as presented herein is based on the dominant fraction of the crystal which was treated as being single in nature.

The asymmetric unit in **3** comprises one (ordered) dication and two Krossing anions, one of which was ordered. The FragmentDB plugin for Olex2 was used to model the -OC($CF_3$)$_3$ groups in the disordered anion. [8] In this structure, the minor component of each of the four disordered dendrites was modelled as a separate residue. As such, the -OC($CF_3$)$_3$ moieties based on O5, O6, O7 and O8 are partnered with residues 3, 1, 4 and 2 respectively, in sequential disorder ratio of 55:45, 70:30, 70:30 and 70:30. Distance and ADP restraints were employed, in the disordered region to assist convergence and the large number of restraints pertain only to the modelling of the anions. Although some of the errors pertaining to these restraints exceed the target sigma values, overall, this converged model provides unambiguous characterisation of this salt in the context of an X-ray experiment.

The asymmetric unit for **4** is comprised of two monomeric calcium cations alongside two aluminate anions. Disorder was identified on the Ca2 containing molecule, present on both of the mesityl units as well as one of the $SiCH_3$ motifs. Two-fold disorder was also identified and readily modelled on the two aluminate anions, however, despite the modelled disorder the aluminate anions elevated the R facors and GooF. ADP and distance restraints were applied to assist satisfactory convergence.

There is one cation and one anion in the asymmetric unit of **5**. The former was ordered and, not unsurprisingly, the pendant groups in the aluminate were disordered in a manner typical of these large spherical anions. In this instance, a 60:40 split was modelled for each of the –OC($CF_3$)$_3$ moieties and all 8 components were modelled as separate residues fitted to the evident electron density. This was effected using the invaluable FragmentDB plugin for Olex2.[8] The restraints in the refinement all pertain to the anion.

The asymmetric unit for **6** is comprised of a monomeric barium unit with a single aluminate counter anion. Twofold disorder was identified and readily modelled across the OC($CF_3$)$_3$ groups, which despite resulting in higher refinement parameters afforded more representative fluorine ADPs. In order to assist convergence, ADP and distance restraints were implemented.

The asymmetric unit for **7** contains a dimeric Sr molecule, one side coordinated to a ClPh motif, while the other coordinated to an aluminate anion *via* two of the $CF_3$ groups; there is a charge separated aluminate anion also present within the asymmetric unit. Attempts to model disorder based upon the charge separated aluminate anion were unsuccessful even through the use of the FragmentDB plugin for Olex2.[8] As such, ADP and distance restraints were required for the single charge separated aluminate anion, consequently resulting in high $R_1$, $wR_2$, Rint and GooF values. The bond metrics therefore are deemed unreliable and as such **7** is sufficient for connectivity only.

The asymmetric unit in **8** is comprised of two aluminate anions and two half barium dimers, the latter can be completed by virtue of inversion symmetry: $^1$1-X,2-Y,1-Z; $^2$2-X,-Y,1-Z. No disorder was

identified on the barium component, though somewhat oblate thermal ellipsoids are present for the trifluoromethyl motifs on the aluminate units. Satisfactory disorder modelling could not be achieved on the trifluoromethyl components and therefore ADP and distance restraints were implemented to assist convergence.

***Table S1.*** *Crystal Data and Structure Refinement for Compounds **1-4**.*

| | | | | |
|---|---|---|---|---|
| Identification code | e22msh07 (**1**) | s22msh71 (**2**) | s22msh66 (**3**) | y26msh11 (**4**) |
| Empirical formula | $C_{56}H_{48}Al_2Ca_2F_{72}N_2O_8Si_4$ | $C_{80}H_{72}Al_2F_{72}N_2O_8Si_4Sr_2$ | $C_{56}H_{48}Al_2Ba_2F_{72}N_2O_8Si_4$ | $C_{80}H_{84}Al_2Ca_2F_{72}N_2O_8Si_4$ |
| Formula weight | 2491.44 | 2898.95 | 2685.96 | 2815.97 |
| Crystal system | orthorhombic | monoclinic | monoclinic | orthorhombic |
| Space group | $Pca2_1$ | $P2_1/n$ | $P2_1$ | $Pna2_1$ |
| $a$/ Å | 26.3292(6) | 16.0259(4) | 15.1422(3) | 22.94080(10) |
| $b$/ Å | 16.4493(4) | 26.7426(6) | 20.8849(3) | 23.57920(10) |
| $c$/ Å | 20.8410(4) | 26.1621(6) | 15.9006(3) | 20.88910(10) |
| $\alpha$/ ° | 90 | 90 | 90 | 90 |
| $\beta$/ ° | 90 | 94.283(2) | 113.986(2) | 90 |
| $\gamma$/ ° | 90 | 90 | 90 | 90 |
| $U$/ Å$^3$ | 9026.2(4) | 11181.1(4) | 4594.22(16) | 11299.45(9) |
| $Z$ | 4 | 4 | 2 | 4 |
| $\rho_{calc}$/ g cm$^{-3}$ | 1.833 | 1.722 | 1.942 | 1.655 |
| $\mu$/ mm$^{-1}$ | 0.397 | 3.406 | 9.143 | 3.002 |
| $F$(000) | 4928.0 | 5744.0 | 2608.0 | 5648.0 |
| Crystal size/ mm$^3$ | 0.477 × 0.375 × 0.084 | 0.239 × 0.206 × 0.125 | 0.268 × 0.128 × 0.079 | 0.24 × 0.23 × 0.19 |
| 2Θ range for data collection/° | 5.84 to 55.022 | 7.086 to 151.074 | 6.8 to 147.17 | 5.374 to 160.868 |
| Index ranges | $-34 \le h \le 33, -21 \le k \le 20, -27 \le l \le 27$ | $-19 \le h \le 19, -33 \le k \le 33, -24 \le l \le 32$ | $-18 \le h \le 12, -25 \le k \le 25, -19 \le l \le 19$ | $-28 \le h \le 29, -28 \le k \le 29, -26 \le l \le 26$ |
| Reflections collected | 76731 | 108698 | 62501 | 160575 |
| Independent reflections, $R_{int}$ | 20673, 0.0477 | 22350, 0.0863 | 17951, 0.0567 | 239940. 0.0362 |
| Data/restraints/parameters | 20673/32767/2167 | 22350/186/1549 | 17951/4470/1807 | 23994/1394/1657 |
| Goodness–of–fit on $F^2$ | 1.030 | 1.035 | 1.019 | 1.632 |
| Final $R1$, $wR2$ [$I$>=2σ($I$)] | 0.0730, 0.1875 | 0.0595, 0.1431 | 0.0588, 0.1587 | 0.1111, 0.3366 |
| Final $R1$, $wR2$ [all data] | 0.1091, 0.2161 | 0.0936, 0.1643 | 0.0611, 0.1626 | 0.1154, 0.3459 |
| Largest diff. peak/hole/ e Å$^{-3}$ | 1.03/-0.47 | 0.79/-1.01 | 0.83/-1.82 | 1.42/-0.77 |

***Table S2.*** *Crystal Data and Structure Refinement for Compounds **5** - **8**.*

| | | | | |
|---|---|---|---|---|
| Identification code | s22msh77 (**5**) | s22msh78 (**6**) | y26msh14 (**7**) | y26msh08 (**8**) |
| Empirical formula | $C_{40}H_{42}AlF_{36}NO_4Si_2Sr$ | $C_{40}H_{42}AlBaF_{36}NO_4Si_2$ | $C_{50}H_{41}Al_2ClF_{72}N_2O_8Si_4Sr_2$ | $C_{60}H_{68}Al_2Ba_2F_{72}N_2O_{12}Si_4$ |
| Formula weight | 1455.52 | 1505.24 | 2542.86 | 2818.16 |
| Crystal system | monoclinic | monoclinic | monoclinic | monoclinic |
| Space group | *Cc* | *Cc* | $P2_1/n$ | $P2_1/c$ |
| *a*/ Å | 15.4604(1) | 15.4771(7) | 11.1460(3) | 24.3917(4) |
| *b*/ Å | 17.9388(2) | 18.1085(9) | 26.0322(6) | 20.6162(3) |
| *c*/ Å | 20.7194(2) | 20.9178(10) | 29.7418(7) | 20.8347(3) |
| *α*/ ° | 90 | 90 | 90 | 90 |
| *β*/ ° | 96.1960(10) | 96.952(4) | 91.486(2) | 106.836(2) |
| *γ*/ ° | 90 | 90 | 90 | 90 |
| *U*/ Å$^3$ | 5712.77(9) | 5819.5(5) | 8626.8(4) | 10028.0(3) |
| *Z* | 4 | 4 | 4 | 4 |
| $\rho_{calc}$/ g cm$^{-3}$ | 1.692 | 1.718 | 1.958 | 1.867 |
| $\mu$/ mm$^{-1}$ | 3.333 | 7.293 | 4.577 | 8.441 |
| *F*(000) | 2896.0 | 2968.0 | 4968.0 | 5520.0 |
| Crystal size/ mm$^3$ | 0.21 × 0.141 × 0.13 | 0.18 × 0.1 × 0.1 | 0.1 × 0.07 × 0.05 | 0.36 × 0.15 × 0.08 |
| 2Θ range for data collection/° | 7.574 to 145.832 | 8.316 to 147.776 | 4.512 to 160.784 | 3.784 to 175.972 |
| Index ranges | -19 ≤ h ≤ 17, -22 ≤ k ≤ 21, -25 ≤ l ≤ 24 | -16 ≤ h ≤ 19, -21 ≤ k ≤ 20, -25 ≤ l ≤ 25 | -14 ≤ h ≤ 11, -33 ≤ k ≤ 30, -35 ≤ l ≤ 36 | -31 ≤ h ≤ 27, -26 ≤ k ≤ 25, -26 ≤ l ≤ 25 |
| Reflections collected | 30773 | 13786 | 103348 | 143206 |
| Independent reflections, $R_{int}$ | 9906, 0.0271 | 7868, 0.0353 | 18194, 0.2563 | 21762, 0.1252 |
| Data/restraints/parameters | 9906/11418/1269 | 7868/1206/685 | 18194/1128/1117 | 21762/2178/1400 |
| Goodness–of–fit on $F^2$ | 1.033 | 1.148 | 1.991 | 1.086 |
| Final *R*1, *wR*2 [$I$>=2σ($I$)] | 0.0707, 0.1924 | 0.0983, 0.2558 | 0.2288, 0.5386 | 0.0861, 0.2363 |
| Final *R*1, *wR*2 [all data] | 0.0714, 0.1936 | 0.1073, 0.2692 | 0.2996, 0.5987 | 0.1135, 0.2602 |
| Largest diff. peak/hole/ e Å$^{-3}$ | 0.98/-0.71 | 1.94/-1.14 | 0.09/-0.05 | 1.70/-1.08 |

**Computational Details**

In Gaussian 16 (C.01.),[9] BP86 geometry optimisations were carried out using the 'grid=ultrafine' option.[10] The def2-SVP split-valence polarized basis set was employed for C, H, N, Si, and Ca, while the corresponding def2 effective core potentials (def2-ECPs) were used for Sr and Ba (BS1).[11] All stationary points were confirmed as minima via analytical frequency calculations through the presence of all positive eigenvalues. Dispersion corrections used Grimme's D3 parameter set with Becke Johnson damping (-D3$^{BJ}$)[12] and corrections for the effect of benzene ($\varepsilon$ = 2.2706) and mesitylene ($\varepsilon$ = 2.2650) were employed using the conductor-like polarizable continuum model (CPCM).[13] All energies were recomputed using a larger basis set. Method A employs the def2-TZVPP basis set (BS2). In method B the all-electron ZORA-def-TZVPP basis set for all atoms was used except for Sr and Ba where ZORA(-SARC-)TZVPP was instead employed (BS3) using ORCA 6.1.0.[14-16] In method B, corrections for dispersion (D3$^{BJ}$) and solvent for benzene and mesitylene (CPCM) were also employed using ORCA 6.1.0.

**Method A:** BP86-D3$^{BJ}$(CPCM)/def2-TZVPP//BP86/def2-SVP

**Method B:** BP86-D3$^{BJ}$(CPCM)/ZORA-def2-TZVPP/SARC//BP86/def2-SVP

Natural bond orbital (NBO) analyses were carried out using NBO7 within Gaussian 16 (C.01.) with BS2.[17] Second order perturbation theory donor-acceptor interactions ("$\Delta E^{(2)}$") between molecular fragments of the structures were estimated with analysis of the Fock matrix in the NBO basis, as calculated by NBO7. Canonical molecular orbitals and Natural Bonding Orbitals are represented using the Chemcraft program.[18] Energy decomposition analysis with natural orbitals for chemical valence (EDA-NOCV) was performed in the gas phase using ORCA 6.1.0 at both the method A and method B levels of theory, without the use of a continuum solvation model.[19]

## Geometries of $1^{Ben} – 6^{Ben}$ and $1^{Mes} – 6^{Mes}$

The bimetallic dications denoted as $1^{Ben}$ – $3^{Ben}$ are equivalent to the dicationic components of the experimentally identified $\eta^6$-benzene arene complexes **1 – 3.** Equivalent mesitylene congeners have not be experimentally identified, but have been modelled computationally, and are referred to as $1^{Mes} – 3^{Mes}$.

The monometallic species denoted a $4^{Mes} – 6^{Mes}$ with two mesitylene arenes coordinated to each metallic centre correspond to the synthesised complexes, **4 - 6**. Equivalent benzene congeners do not form experimentally, but have been modelled computationally, and are referred to as $4^{Ben} – 6^{Ben}$.

The following pages depict all structures that have been computationally modelled. The atom colour code is: grey = C, carbon; light brown = Si, silicon; blue = N, nitrogen; light green = Ca, Sr or Ba, calcium, strontium or barium.

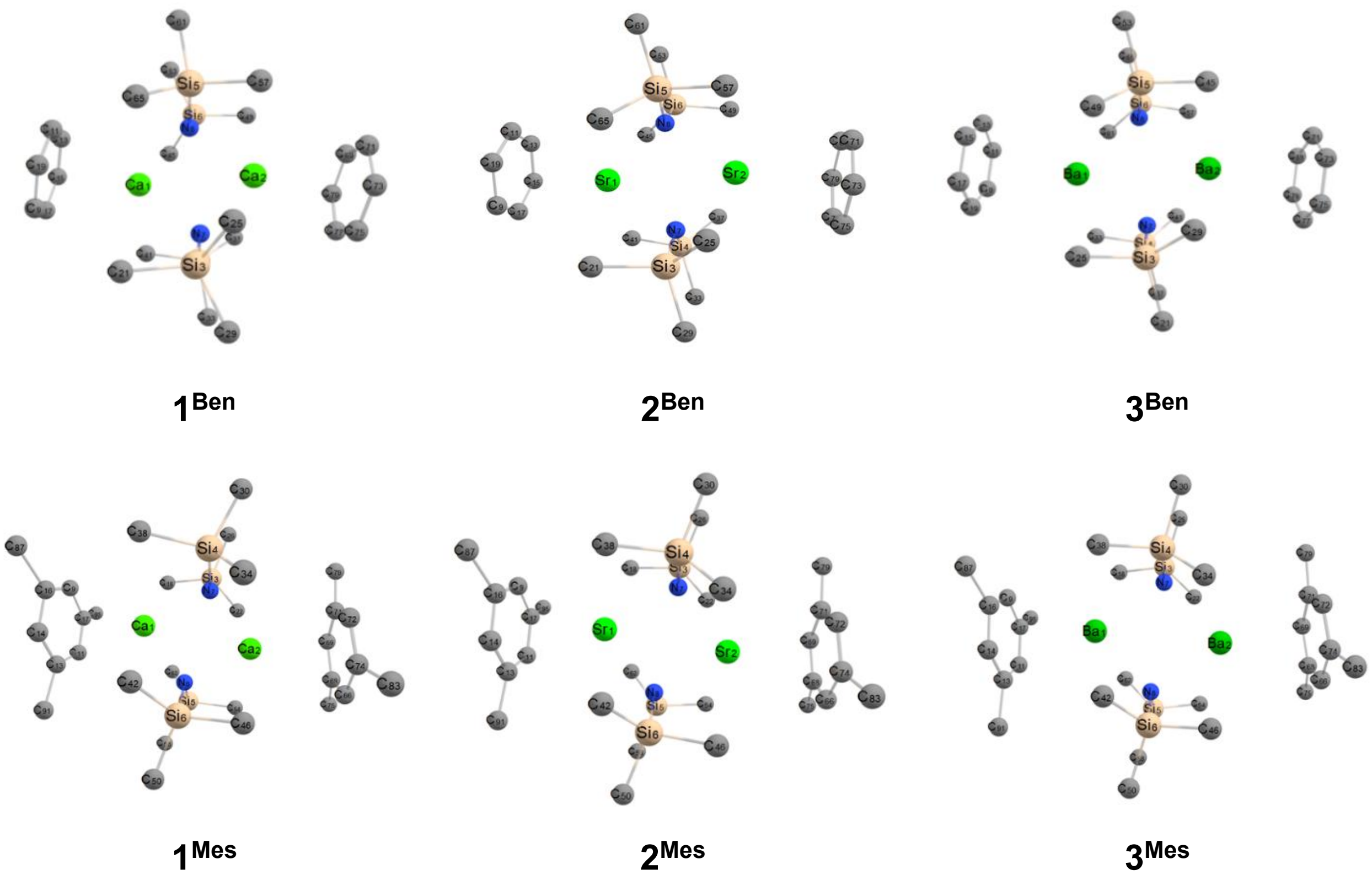


**Figure S17.** BP86/def2-SVP optimised structures of complexes $1^{Ben}$ – $3^{Ben}$ and $1^{Mes} – 3^{Mes}$**.** Hydrogen atoms have been omitted for clarity.

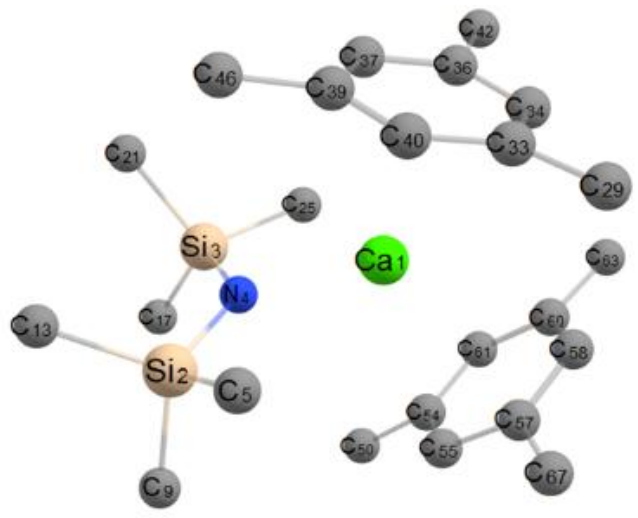

**4**$^{\mathbf{Mes}}$

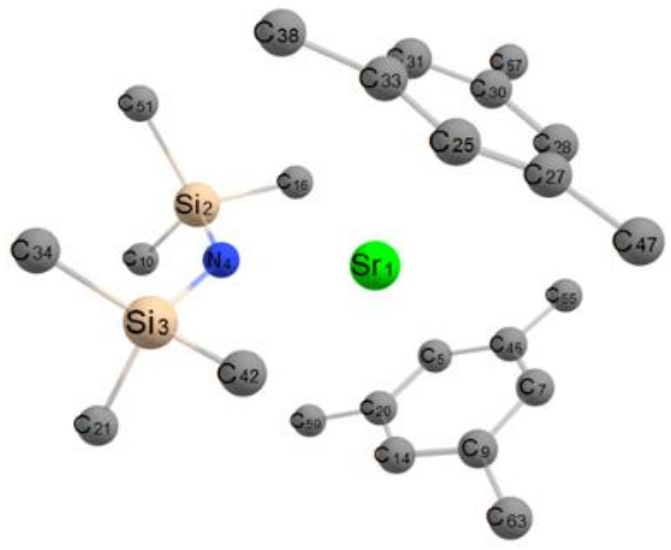

**5**$^{\mathbf{Mes}}$

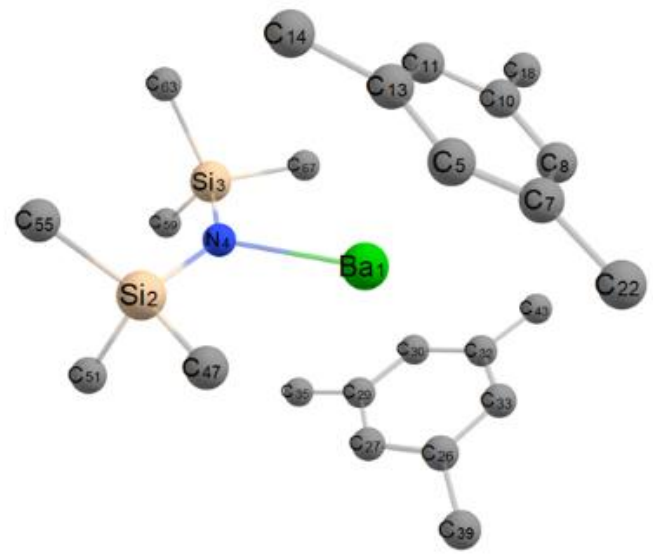

**6**$^{\mathbf{Mes}}$

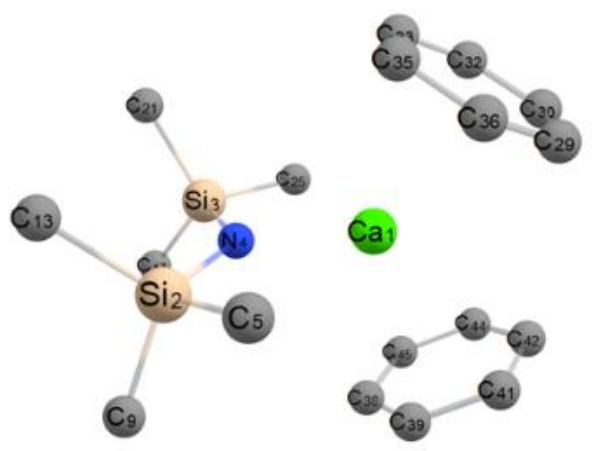

**4**$^{\mathbf{Ben}}$

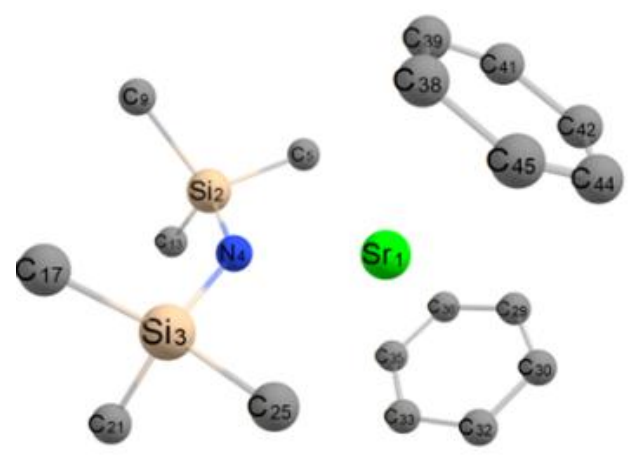

**5**$^{\mathbf{Ben}}$

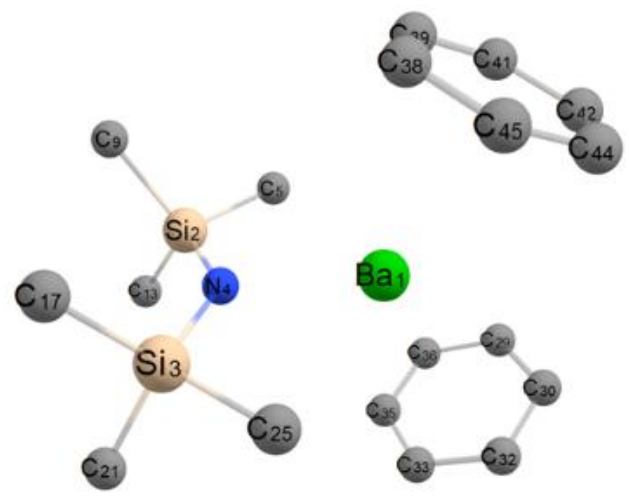

**6**$^{\mathbf{Ben}}$

**Figure S18.** BP86/def2-SVP optimised structures of complexes **4**$^{\mathbf{Mes}}$ – **6**$^{\mathbf{Mes}}$ and **4**$^{\mathbf{Ben}}$ – **6**$^{\mathbf{Ben}}$**.** Hydrogen atoms have been omitted for clarity.

**Table S3.** Ae-centroid distances **a** and **b** (Å) for compounds **1**$^{\mathbf{Ben}}$ – **3**$^{\mathbf{Ben}}$ and **1**$^{\mathbf{Mes}}$ – **3**$^{\mathbf{Mes}}$.

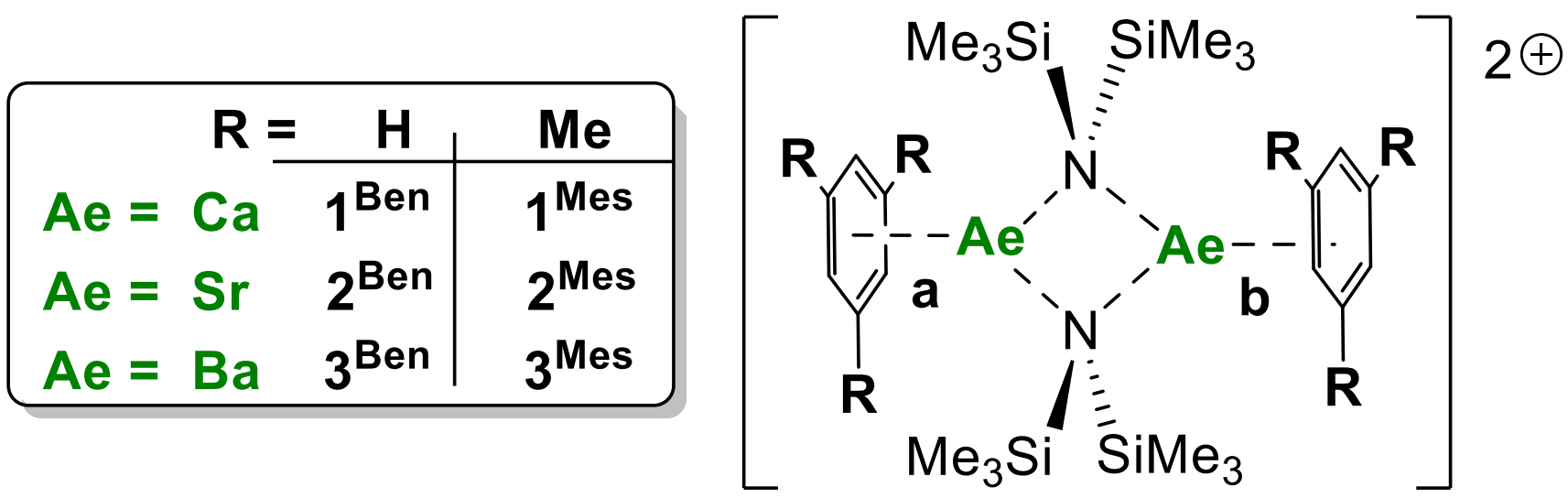


| | **1**$^{\mathbf{Ben}}$ | **2**$^{\mathbf{Ben}}$ | **3**$^{\mathbf{Ben}}$ |
|---|---|---|---|
| **a** | 2.785 | 2.938 | 3.126 |
| **b** | 2.786 | 2.938 | 3.126 |
| | **1**$^{\mathbf{Mes}}$ | **2**$^{\mathbf{Mes}}$ | **3**$^{\mathbf{Mes}}$ |
| **a** | 2.815 | 2.896 | 3.057 |
| **b** | 2.815 | 2.896 | 3.057 |

**Table S4.** Ae-centroid distances **a** and **b** (Å) for compounds **4**$^{\mathbf{Ben}}$ – **6**$^{\mathbf{Ben}}$ and **4**$^{\mathbf{Mes}}$ – **6**$^{\mathbf{Mes}}$.

.

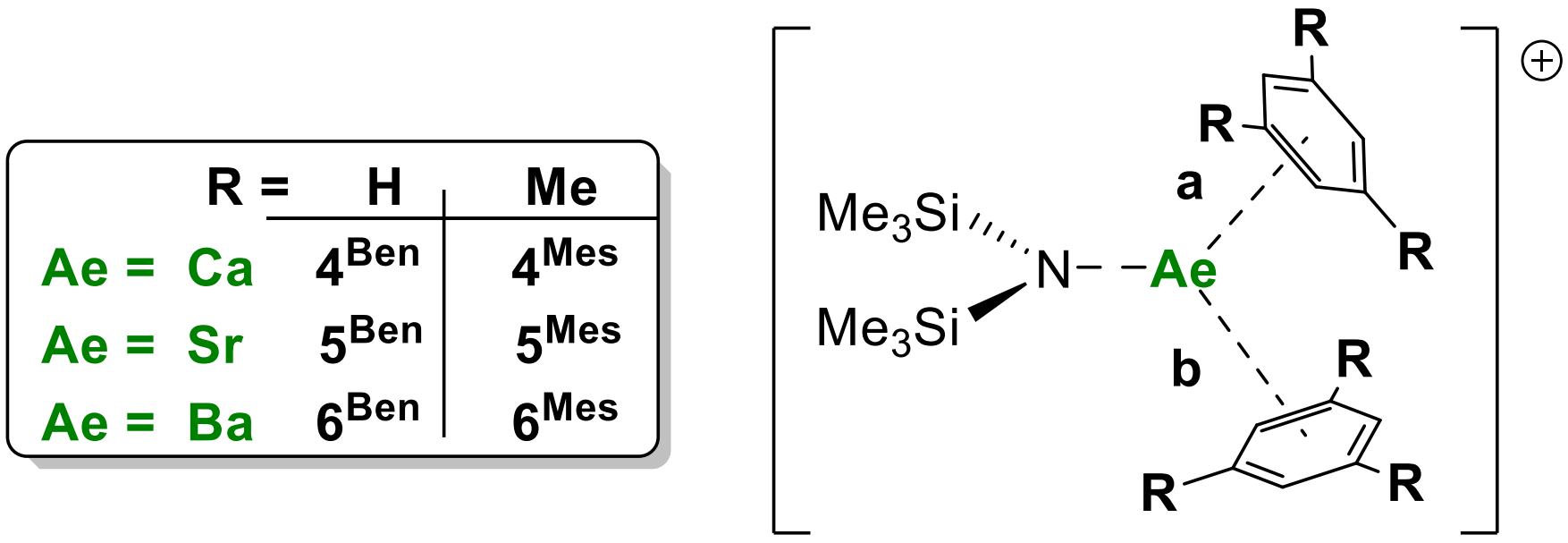


| | **4**$^{\mathbf{Mes}}$ | **5**$^{\mathbf{Mes}}$ | **6**$^{\mathbf{Mes}}$ |
|---|---|---|---|
| **a** | 2.684 | 2.868 | 3.070 |
| **b** | 2.708 | 2.868 | 3.070 |
| | **4**$^{\mathbf{Ben}}$ | **5**$^{\mathbf{Ben}}$ | **6**$^{\mathbf{Ben}}$ |
| **a** | 2.707 | 2.915 | 3.127 |
| **b** | 2.714 | 2.915 | 3.128 |

## Breakdown of Energy Contributions

**Table S5.** Detailed contributions to relative energies as the successive corrections to the initial SCF energy are included. Terms used are:

| | |
|---|---|
| $\Delta E_{BS1}$ | SCF energy computed with the BP86 functional and BS1 |
| $\Delta G_{BS1}$ | Free energy computed at 298.15 K and 1 atm with the BP86 functional and BS1 |
| $\Delta G_{BS1/CPCM}$ | Free energy corrected for benzene/mesitylene solvent using CPCM with the BP86 functional and BS1 |
| $\Delta G_{BS1/D3^{BJ}/CPCM}$ | Free energy corrected for benzene/mesitylene solvent using CPCM and dispersion (-D3$^{BJ}$) with the BP86 functional and BS1 |
| $\Delta E_{BS2}$ | SCF energy computed with the BP86 functional and BS2 |
| $\Delta E_{BS2/D3^{BJ}/CPCM}$ | SCF energy corrected for benzene/mesitylene solvent using CPCM and dispersion (-D3$^{BJ}$) with the BP86 functional and BS2 |
| $\Delta E_{BS3/D3^{BJ}/CPCM}$ | SCF energy corrected for benzene/mesitylene solvent using CPCM and dispersion (-D3$^{BJ}$) with the BP86 functional and BS3 |
| $\Delta G_A$ | Overall free energy computed at the BP86-D3$^{BJ}$(CPCM)/def2-TZVPP//BP86/def2-SVP level |
| $\Delta G_B$ | Overall free energy computed at the BP86-D3$^{BJ}$(CPCM)/ZORA-def2-TZVPP/SARC//BP86/def2-SVP level |

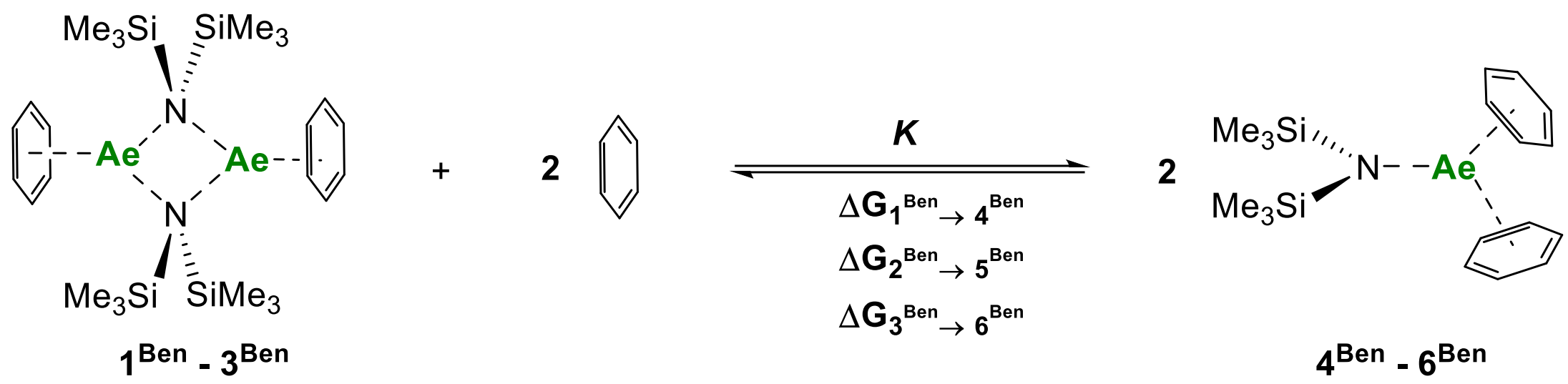


**Scheme S1**. Interchange reactions benzene complexes **1**$^{\mathbf{Ben}}$ **- 3**$^{\mathbf{Ben}}$ and **4**$^{\mathbf{Ben}}$ **– 6**$^{\mathbf{Ben}}$.

**Table S6.** Summary of Gibbs free energies (in kcal mol$^{-1}$) and equilibrium constants (298.15 K) for the interchange reactions shown in Scheme S1 using methods **A** and **B**.

| | [kcal mol$^{-1}$] | | | |
|---|---|---|---|---|
| | **A** | **B** | **K$_A$** | **K$_B$** |
| $\Delta G_{1^{Ben}\rightarrow 4^{Ben}}$ | 3.5 | **2.4** | 2.70 x $10^{-3}$ | 1.79 x $10^{-2}$ |
| $\Delta G_{2^{Ben}\rightarrow 5^{Ben}}$ | 4.8 | **4.5** | 3.01 x $10^{-4}$ | 5.21 x $10^{-4}$ |
| $\Delta G_{3^{Ben}\rightarrow 6^{Ben}}$ | -0.1 | **1.3** | 1.27 | 0.11 |

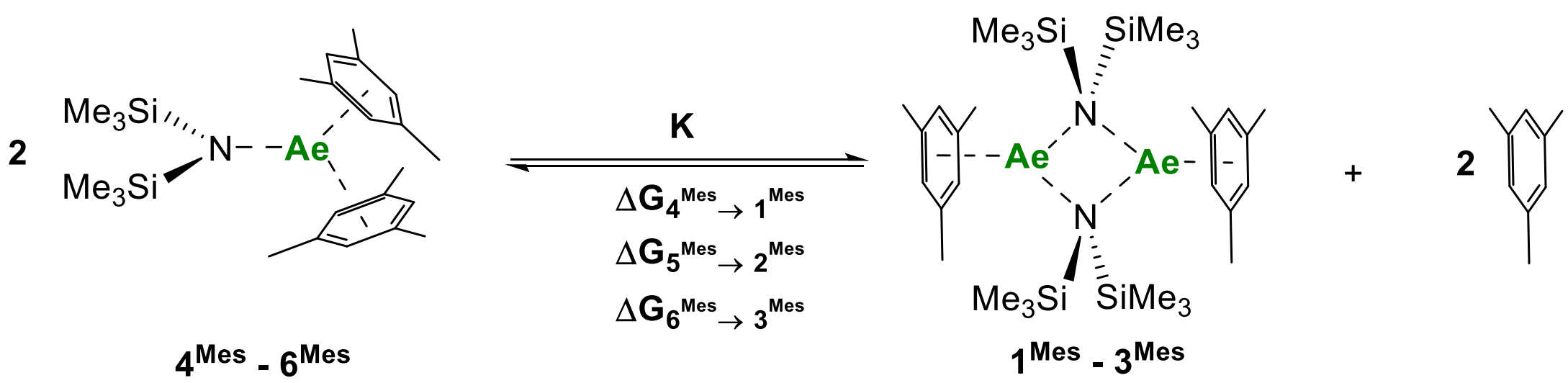


**Scheme S2**. Interchange reactions between mesitylene complexes **4**$^{\mathbf{Mes}}$ **– 6**$^{\mathbf{Mes}}$ and **1**$^{\mathbf{Mes}}$ **– 3**$^{\mathbf{Mes}}$.

**Table S7.** Summary of Gibbs free energies (in kcal mol$^{-1}$) and equilibrium constants (298.15 K) for the interchange reactions shown in Scheme S3 using methods **A** and **B**.

| | [kcal mol$^{-1}$] | | | |
|---|---|---|---|---|
| | **A** | **B** | **K$_A$** | **K$_B$** |
| $\Delta G_{4^{Mes}\rightarrow 1^{Mes}}$ | 5.8 | **5.0** | 6.02 x $10^{-5}$ | 2.35 x $10^{-4}$ |
| $\Delta G_{5^{Mes}\rightarrow 2^{Mes}}$ | 3.1 | **3.2** | 5.63 x $10^{-3}$ | 4.87 x $10^{-3}$ |
| $\Delta G_{6^{Mes}\rightarrow 3^{Mes}}$ | 4.6 | **3.0** | 4.34 x $10^{-4}$ | 6.42 x $10^{-3}$ |

**Table S8.** Relative energy corrections (in kcal mol$^{-1}$) for the interconversions as seen in Schemes **S1** and **S2**, as well as Tables **S6** and **S7**.

| | **$\Delta E_{BS1}$** | **$\Delta G_{BS1}$** | **$\Delta G_{BS1/CPCM}$** | **$\Delta G_{BS1/CPCM/D3}{}^{BJ}$** | **$\Delta E_{BS2}$** | **$\Delta E_{BS2/CPCM/D3}{}^{BJ}$** | **$\Delta E_{BS3/CPCM/D3}{}^{BJ}$** | **$\Delta G_A$** | **$\Delta G_B$** |
|---|---|---|---|---|---|---|---|---|---|
| $\Delta G_{1^{Ben} \rightarrow 4^{Ben}}$ | -35.5 | -31.7 | -5.7 | 6.3 | -38.4 | -0.3 | -1.5 | 3.5 | **2.4** |
| $\Delta G_{2^{Ben} \rightarrow 5^{Ben}}$ | -32.8 | -29.7 | -6.0 | 7.3 | -35.3 | 1.7 | 1.4 | 4.8 | **4.5** |
| $\Delta G_{3^{Ben} \rightarrow 6^{Ben}}$ | -33.3 | -32.7 | -9.5 | 2.7 | -36.1 | -0.7 | 0.7 | -0.1 | **1.3** |
| $\Delta G_{4^{Mes} \rightarrow 1^{Mes}}$ | 41.6 | 31.7 | 7.0 | 4.1 | 43.3 | 15.6 | 14.8 | 5.8 | **5.0** |
| $\Delta G_{5^{Mes} \rightarrow 2^{Mes}}$ | 38.0 | 29.3 | 5.7 | 1.3 | 39.8 | 11.8 | 11.7 | 3.1 | **3.2** |
| $\Delta G_{6^{Mes} \rightarrow 3^{Mes}}$ | 38.2 | 30.8 | 7.4 | 2.7 | 40.1 | 12.0 | 10.6 | 4.6 | **3.0** |

## Natural Bond Orbital Analysis

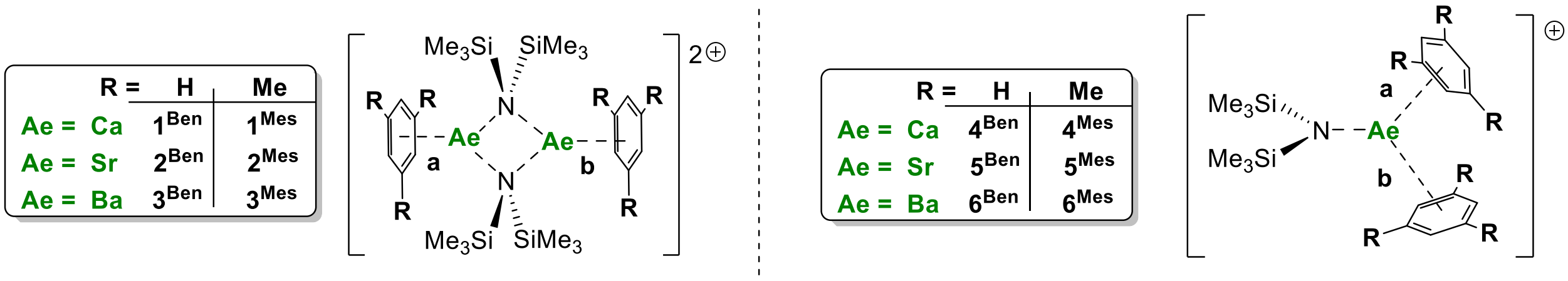


**Figure S19.** Nomenclature used to label the complexes $\mathbf{1}^{\mathbf{Ben}}$ – $\mathbf{6}^{\text{Ben}}$ and $\mathbf{1}^{\mathbf{Mes}}$ – $\mathbf{6}^{\mathbf{Mes}}$ in Tables S9 and S10.

**Table S9.** NBO natural charges for key atoms in $\mathbf{1}^{\mathbf{Ben}}$ – $\mathbf{6}^{\text{Ben}}$ and $\mathbf{1}^{\mathbf{Mes}}$ – $\mathbf{6}^{\mathbf{Mes}}$.

| | $\mathbf{1}^{\mathbf{Ben}}$ | $\mathbf{2}^{\mathbf{Ben}}$ | $\mathbf{3}^{\mathbf{Ben}}$ |
|---|---|---|---|
| $Ae_1$ | 1.777 | 1.809 | 1.816 |
| $Ae_2$ | 1.777 | 1.809 | 1.816 |
| $N_1$ | −1.916 | −1.919 | −1.909 |
| $N_2$ | −1.916 | −1.919 | −1.909 |
| | $\mathbf{1}^{\mathbf{Mes}}$ | $\mathbf{2}^{\mathbf{Mes}}$ | $\mathbf{3}^{\mathbf{Mes}}$ |
| $Ae_1$ | 1.785 | 1.819 | 1.826 |
| $Ae_2$ | 1.785 | 1.819 | 1.826 |
| $N_1$ | −1.912 | −1.918 | −1.906 |
| $N_2$ | −1.912 | −1.918 | −1.906 |
| | $\mathbf{4}^{\mathbf{Mes}}$ | **5Mes** | $\mathbf{6}^{\mathbf{Mes}}$ |
| Ae | 1.752 | 1.795 | 1.806 |
| N | −1.844 | −1.840 | −1.850 |
| | $\mathbf{4}^{\mathbf{Ben}}$ | $\mathbf{5}^{\mathbf{Ben}}$ | $\mathbf{6}^{\mathbf{Ben}}$ |
| Ae | 1.735 | 1.777 | 1.789 |
| N | −1.822 | −1.844 | −1.846 |

**Table S10.** Wiberg Bond Index (WBI) data of $\mathbf{1}^{\mathbf{Ben}}$ – $\mathbf{6}^{\mathbf{Ben}}$ and $\mathbf{1}^{\mathbf{Mes}}$ – $\mathbf{6}^{\mathbf{Mes}}$.

| | $\mathbf{1}^{\mathbf{Ben}}$ | $\mathbf{2}^{\mathbf{Ben}}$ | $\mathbf{3}^{\mathbf{Ben}}$ |
|---|---|---|---|
| $Ae_1$–$N_1$ | 0.082 | 0.067 | 0.065 |
| $Ae_1$–$N_2$ | 0.101 | 0.082 | 0.078 |
| $Ae_2$–$N_1$ | 0.101 | 0.082 | 0.078 |
| $Ae_2$–$N_2$ | 0.082 | 0.067 | 0.065 |
| | $\mathbf{1}^{\mathbf{Mes}}$ | $\mathbf{2}^{\mathbf{Mes}}$ | $\mathbf{3}^{\mathbf{Mes}}$ |
| $Ae_1$–$N_1$ | 0.069 | 0.055 | 0.055 |
| $Ae_1$–$N_2$ | 0.095 | 0.072 | 0.070 |
| $Ae_2$–$N_1$ | 0.095 | 0.072 | 0.070 |
| $Ae_2$–$N_2$ | 0.069 | 0.055 | 0.055 |
| | $\mathbf{4}^{\mathbf{Mes}}$ | $\mathbf{5}^{\mathbf{Mes}}$ | $\mathbf{6}^{\mathbf{Mes}}$ |
| Ae–N | 0.176 | 0.137 | 0.142 |
| | $\mathbf{4}^{\mathbf{Ben}}$ | $\mathbf{5}^{\mathbf{Ben}}$ | $\mathbf{6}^{\mathbf{Ben}}$ |
| Ae–N | 0.219 | 0.176 | 0.178 |

## Canonical Frontier Molecular Orbitals

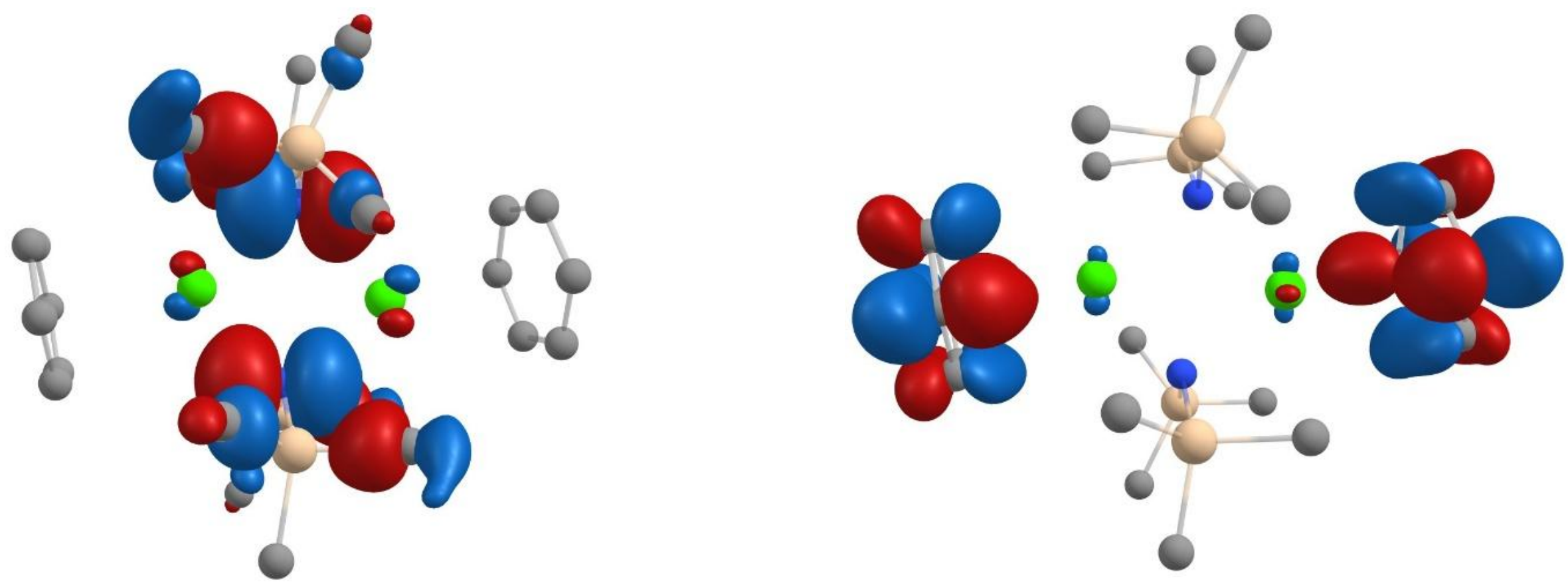

**HOMO**

MO-150 = −11.41 eV

0.546*[68] LP (2) N8(lp)

0.545*[66] LP (2) N7(lp)

**LUMO**

MO-151 = −7.82 eV

0.403*[228] BD*(2) C73- C75

0.398*[179] BD*(2) C13- C15

-0.385*[233] BD*(2) C77- C79

-0.380*[176] BD*(2) C11- C19

0.270*[138] BD (2) C69- C71

0.267*[87] BD (2) C9- C17

**Figure S19.** HOMO and LUMO for $\mathbf{1}^{\mathbf{Ben}}$ (contour value = 0.03).

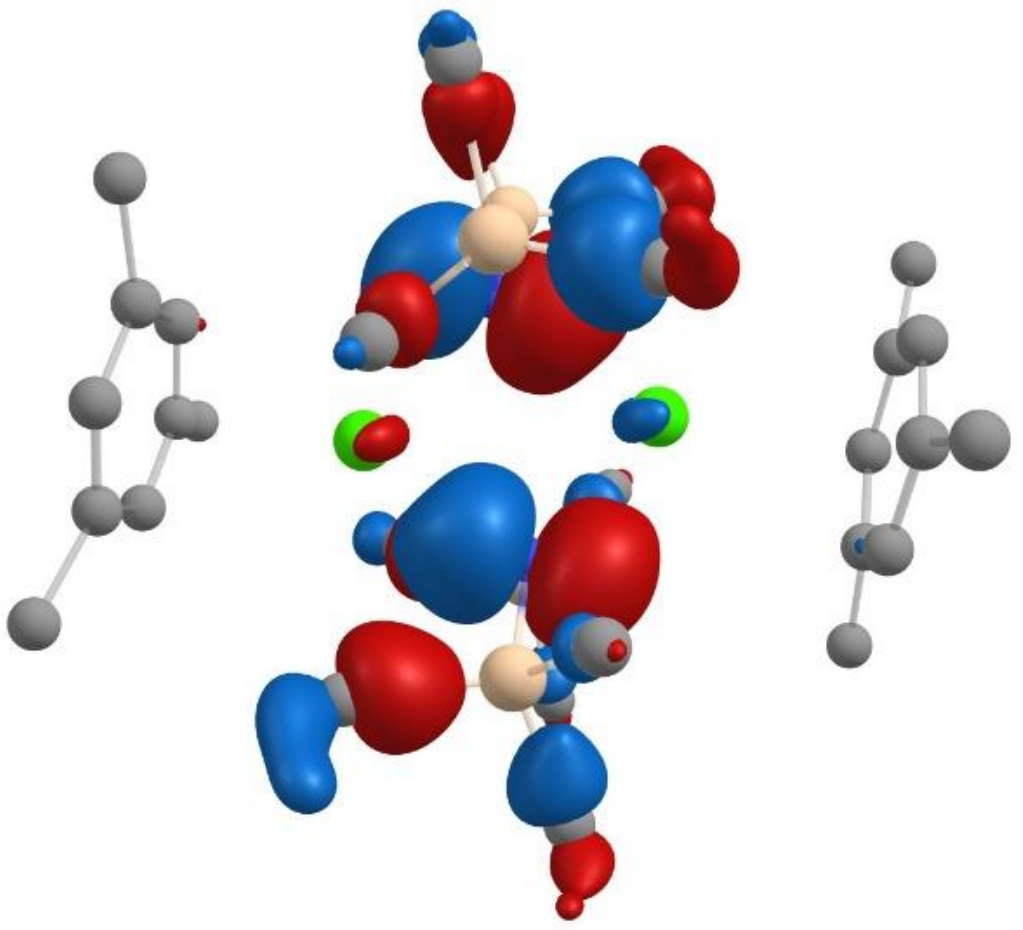

**HOMO**

MO-174 = −11.12 eV

0.535*[74] LP ( 2) N 8(lp)

-0.534*[72] LP ( 2) N 7(lp)

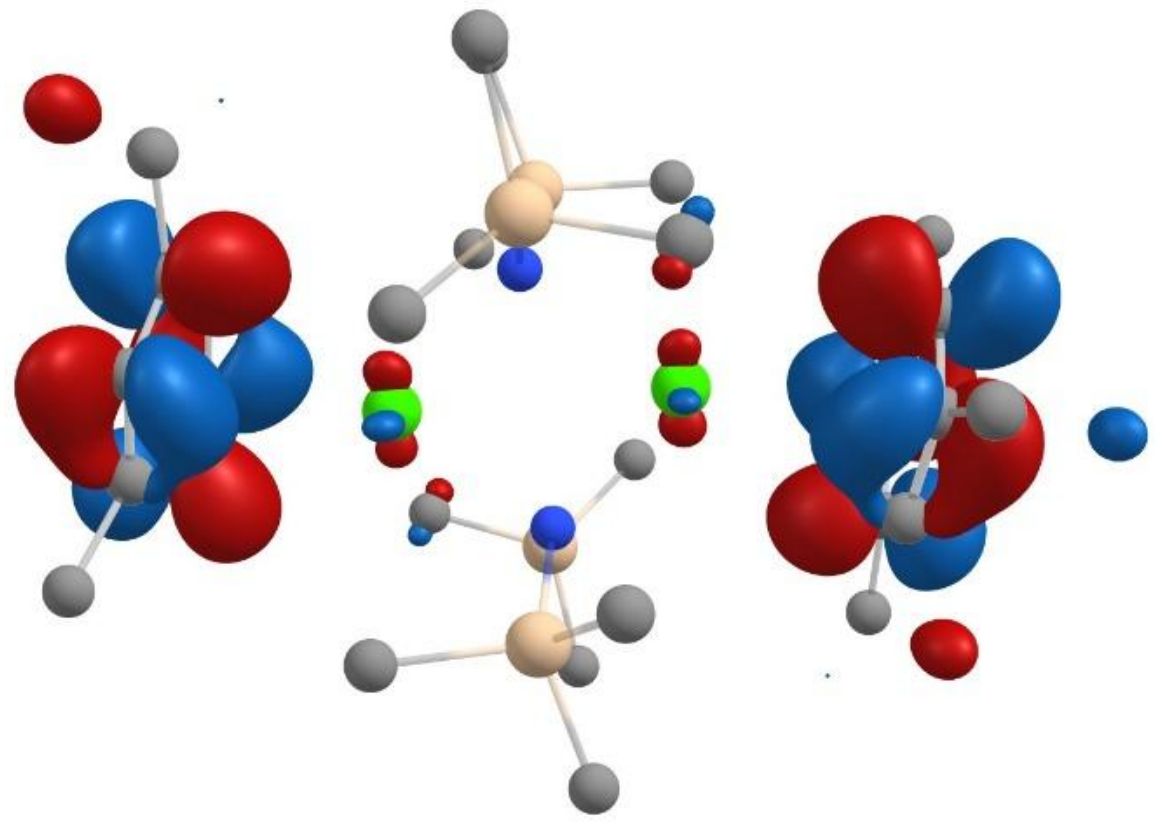

**LUMO**

MO-175 = −7.22 eV

-0.417*[202] BD*(2) C13-C14

-0.416*[252] BD*(2) C69-C71

0.290*[195] BD*(2) C 9-C16

0.289*[246] BD*(2) C66-C68

0.232*[98] BD (2) C11-C17

0.231*[155] BD (2) C72-C74

**Figure S20.** HOMO and LUMO for **1**$^{\mathbf{Mes}}$ (contour value = 0.03).

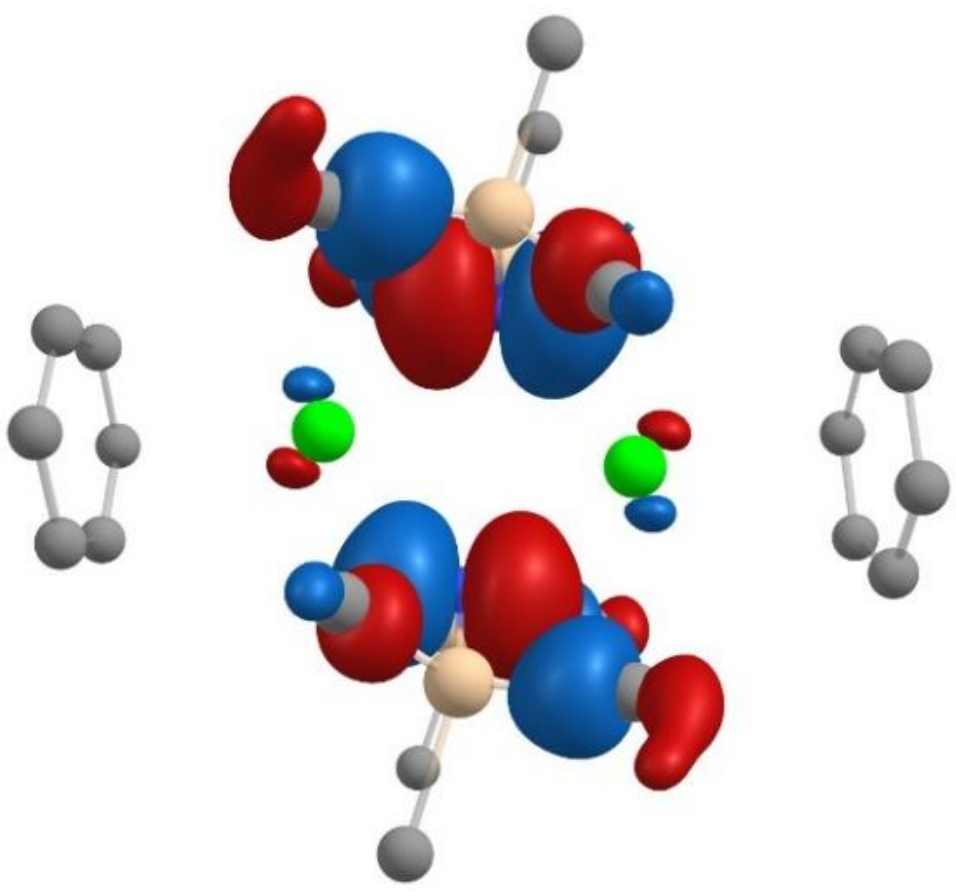

**HOMO**

MO-140 = −11.14 eV

-0.581*[58] LP (2) N8(lp)

-0.546*[56] LP (2) N7(lp)

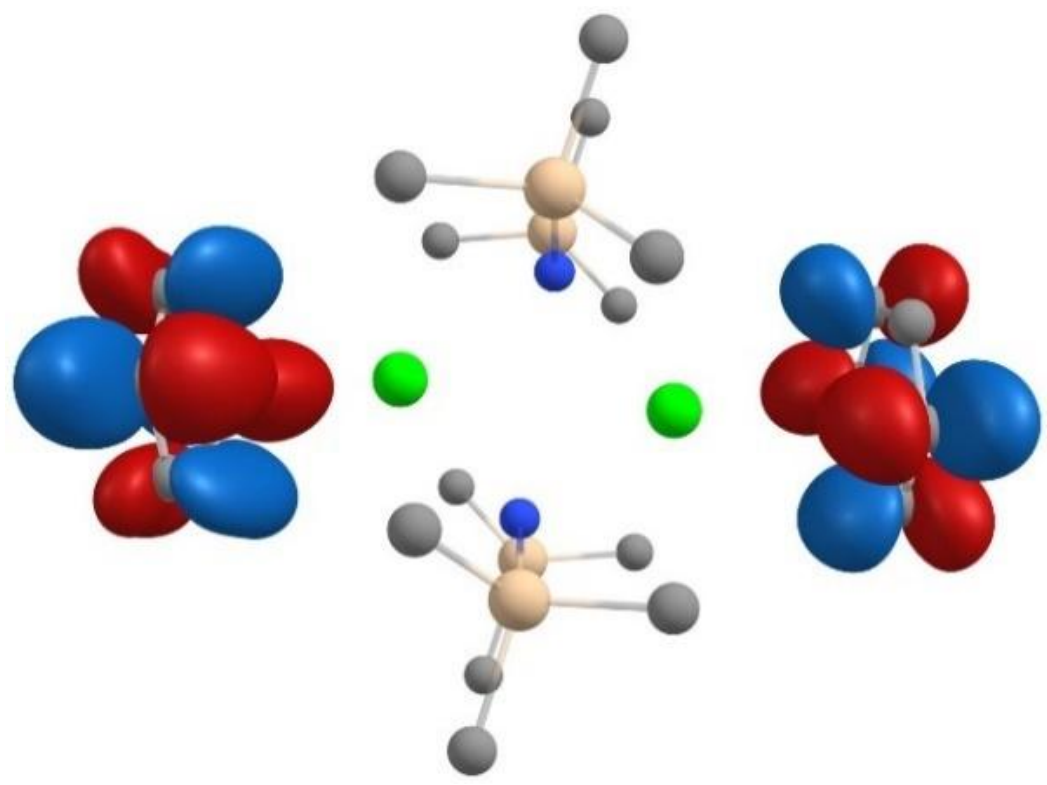

**LUMO**

MO-141 = −7.63 eV

-0.456*[218] BD*(2) C73-C75

-0.420*[223] BD*(2) C77-C79

-0.408*[166] BD*(2) C11-C19

0.297*[128] BD (2) C69-C71

-0.229*[169] BD*(2) C13-C15

**Figure S21.** HOMO and LUMO for **2**$^{\mathbf{Ben}}$ (contour value = 0.03).

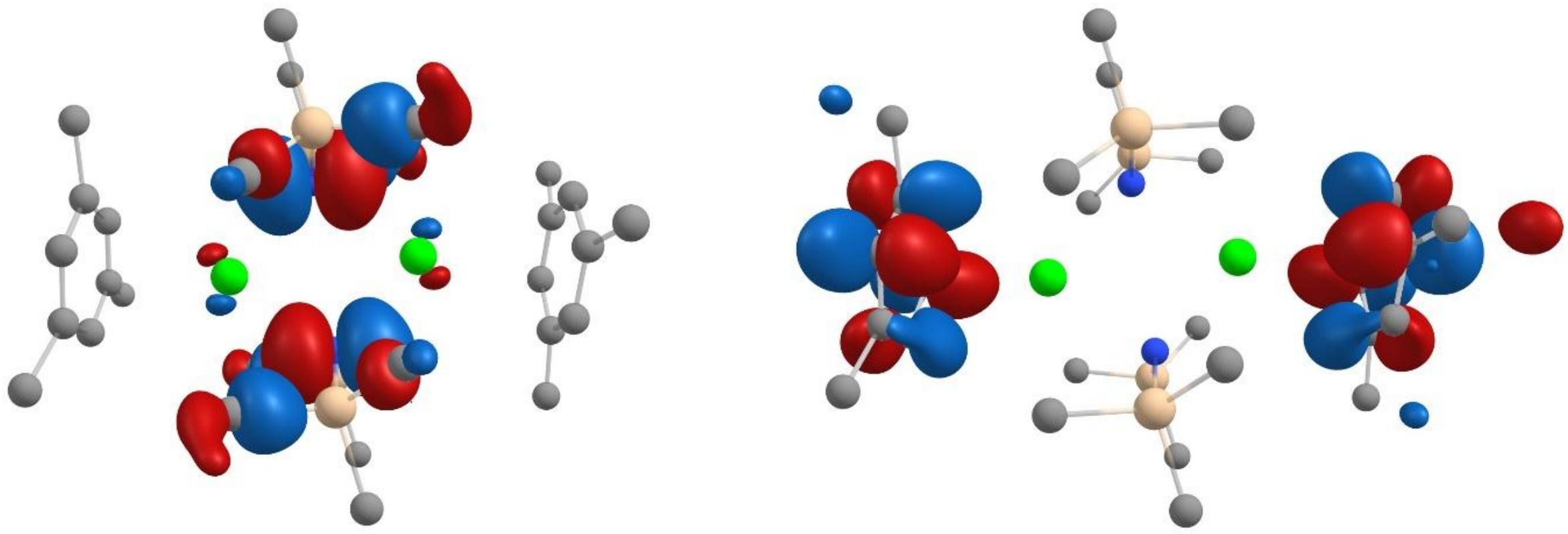

**HOMO**

MO-164 = −10.89 eV

-0.564*[64] LP (2) N 8(lp)

-0.564*[62] LP (2) N 7(lp)

**LUMO**

MO-165 = −7.12 eV

0.419*[242] BD*(2) C69-C71

0.418*[192] BD*(2) C13-C14

-0.310*[247] BD*(2) C72-C74

-0.310*[190] BD*(2) C11-C17

0.249*[134] BD (2) C66-C68

0.248*[ 83] BD (2) C 9-C16

**Figure S22.** HOMO and LUMO for $\mathbf{2}^{\mathbf{Mes}}$ (contour value = 0.03).

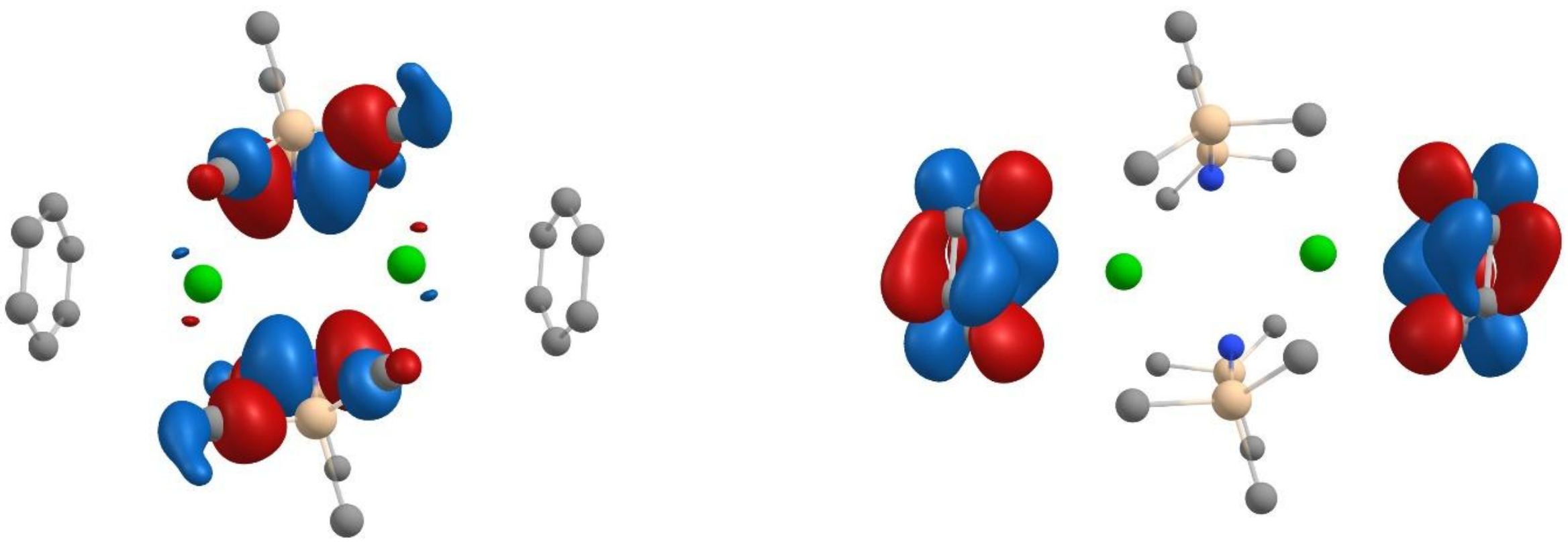

**HOMO**

MO-140 = −10.93 eV

0.575*[56] LP ( 2) N 7(lp)

0.569*[58] LP ( 2) N 8(lp)

**LUMO**

MO-141 = −7.46 eV

0.426*[165] BD*(2) C11-C13

0.424*[223] BD*(2) C77-C7

-0.337*[162] BD*(2) C 9-C19

-0.334*[212] BD*(2) C69-C71

0.254*[86] BD (2) C15-C17

0.252*[134] BD (2) C73-C75

**Figure S23.** HOMO and LUMO for $\mathbf{3}^{\mathbf{Ben}}$ (contour value = 0.03).

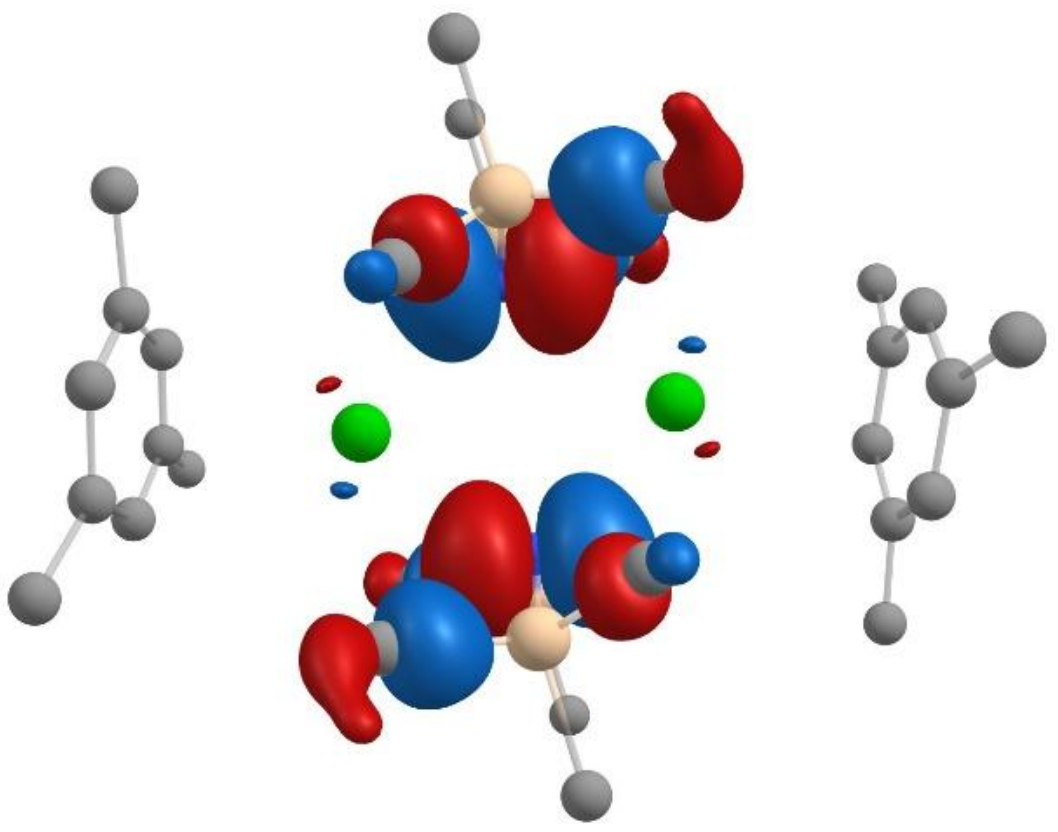

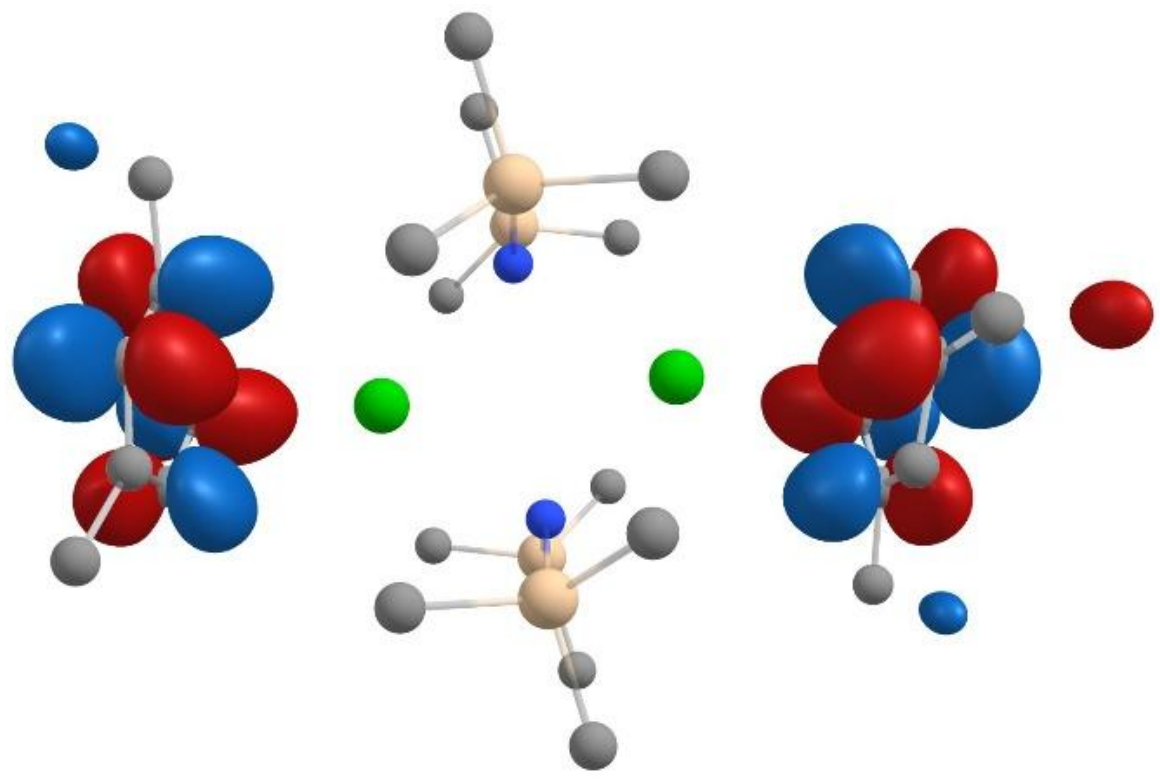

**HOMO**
MO-164 = −10.68 eV
-0.571*[62] LP (2) N 7(lp)
-0.570*[64] LP (2) N 8(lp)

**LUMO**
MO-165 = −7.01 eV
0.412*[242] BD*( 2) C69-C71
0.412*[192] BD*( 2) C13-C14
-0.255*[247] BD*( 2) C72- C74
-0.253*[190] BD*( 2) C11C17
0.227*[134] BD ( 2) C66-C68
0.226*[83] BD ( 2) C9-C16

**Figure S24.** HOMO and LUMO for $\mathbf{3^{Mes}}$ (contour value = 0.03).

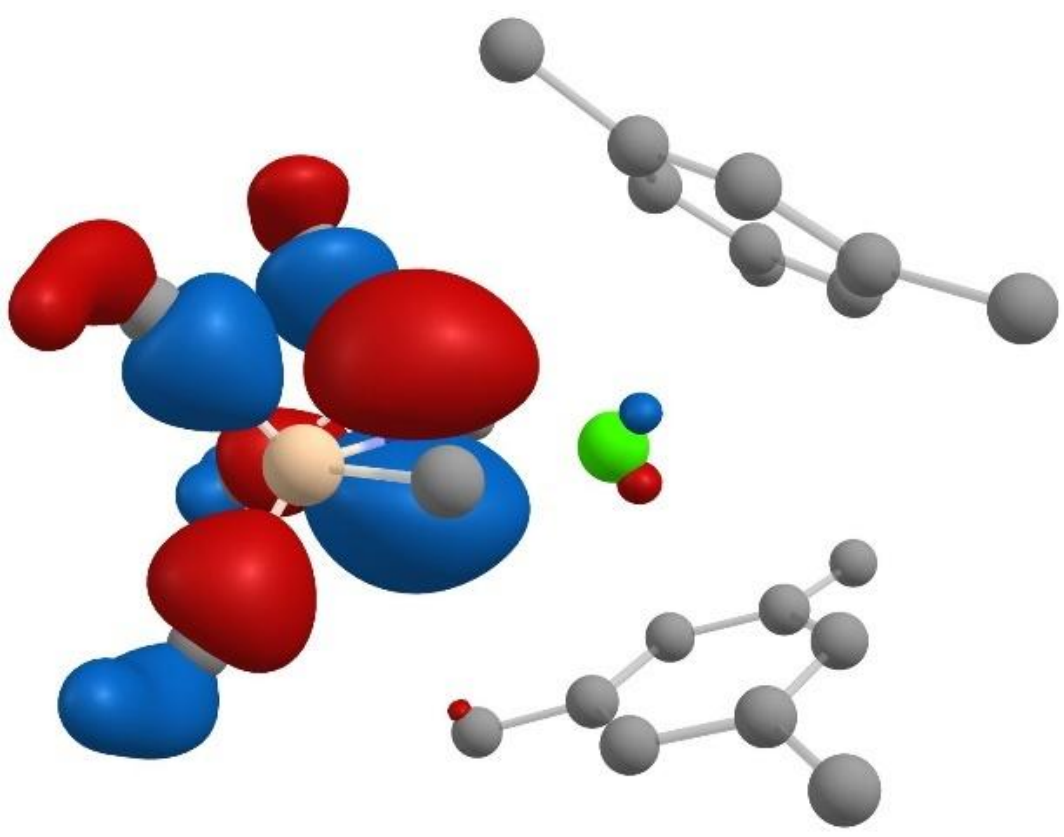

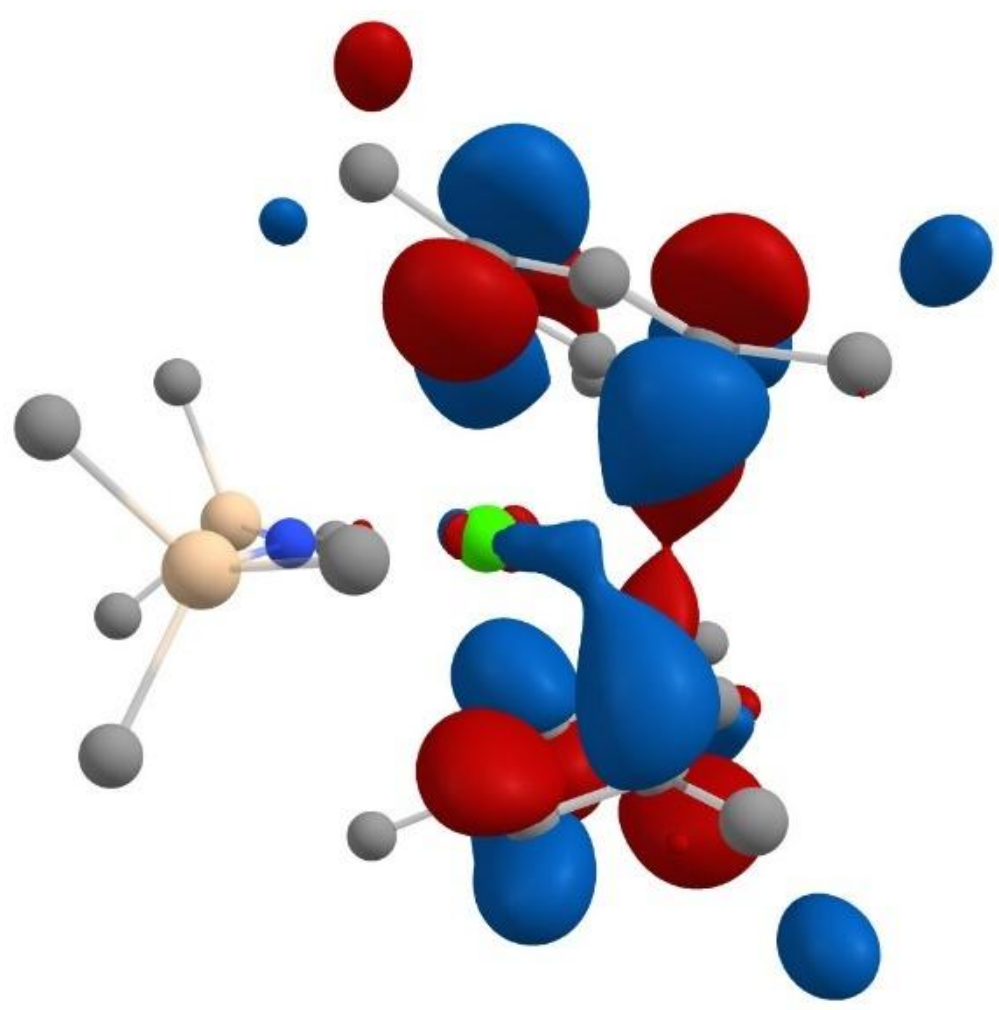

**HOMO**
MO-120 = −7.73 eV
0.819*[46] LP (2) N4(lp)
-0.259*[49] BD (1) Si2- C9

**LUMO**
MO-121 = −5.35 eV
0.466*[186] BD*(2) C58- C60
0.424*[158] BD*(2) C36- C37
-0.288*[163] BD*(2) C39- C40
0.279*[178] BD*(2) C54- C61
0.258*[106] BD (2) C55- C57

**Figure S25.** HOMO and LUMO for $\mathbf{4^{Mes}}$ (contour value = 0.03).

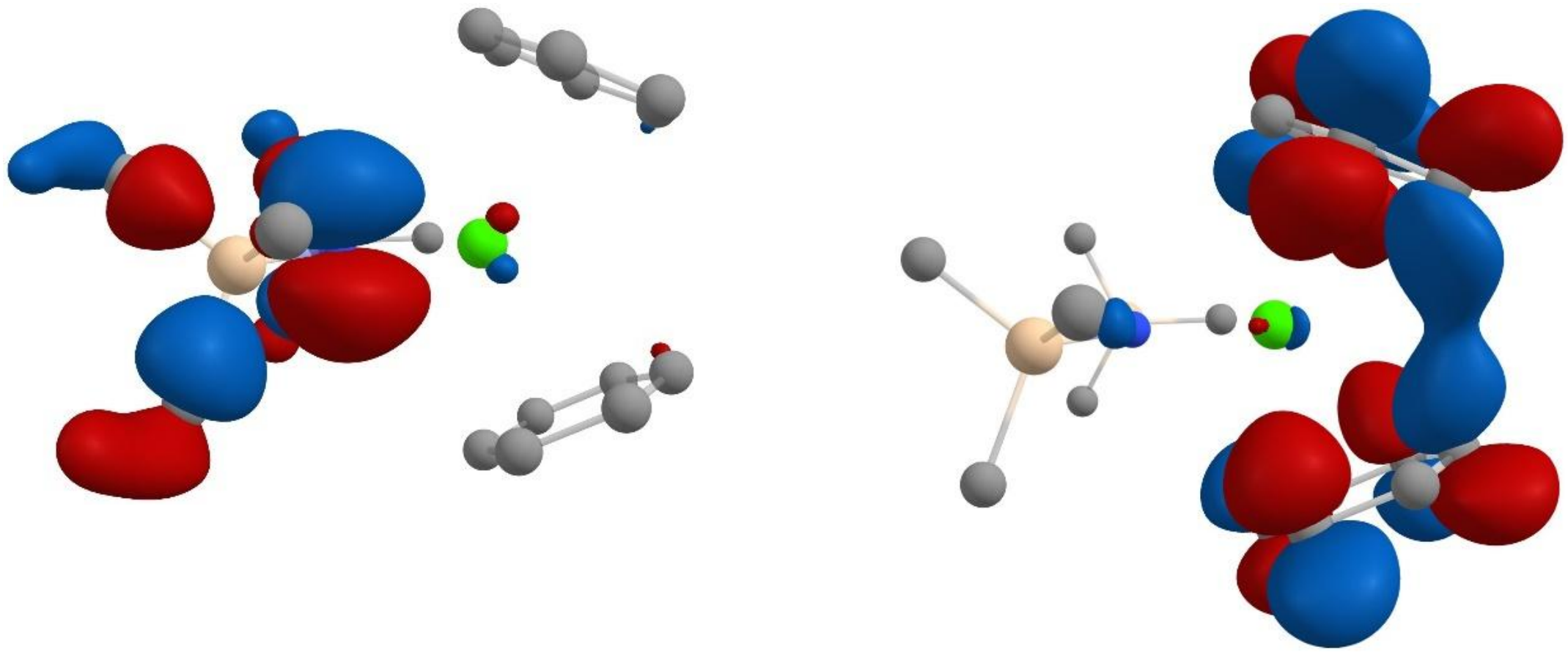

**HOMO**
MO-96 = -7.96 eV
0.812*[40] LP (2) N4(lp)
-0.270*[43] BD (1)Si2- C9

**LUMO**
MO-97 = -5.88 eV
-0.478*[126] BD*(2) C29- C36
0.468*[150] BD*(2) C42- C44
0.263*[145] BD*(2) C39- C41
0.258*[84] BD (2) C38- C45
0.252*[78] BD (2) C33- C35
-0.240*[130] BD (2) C30- C32

**Figure S26.** HOMO and LUMO for **4**$^{\mathbf{Ben}}$ (contour value = 0.03).

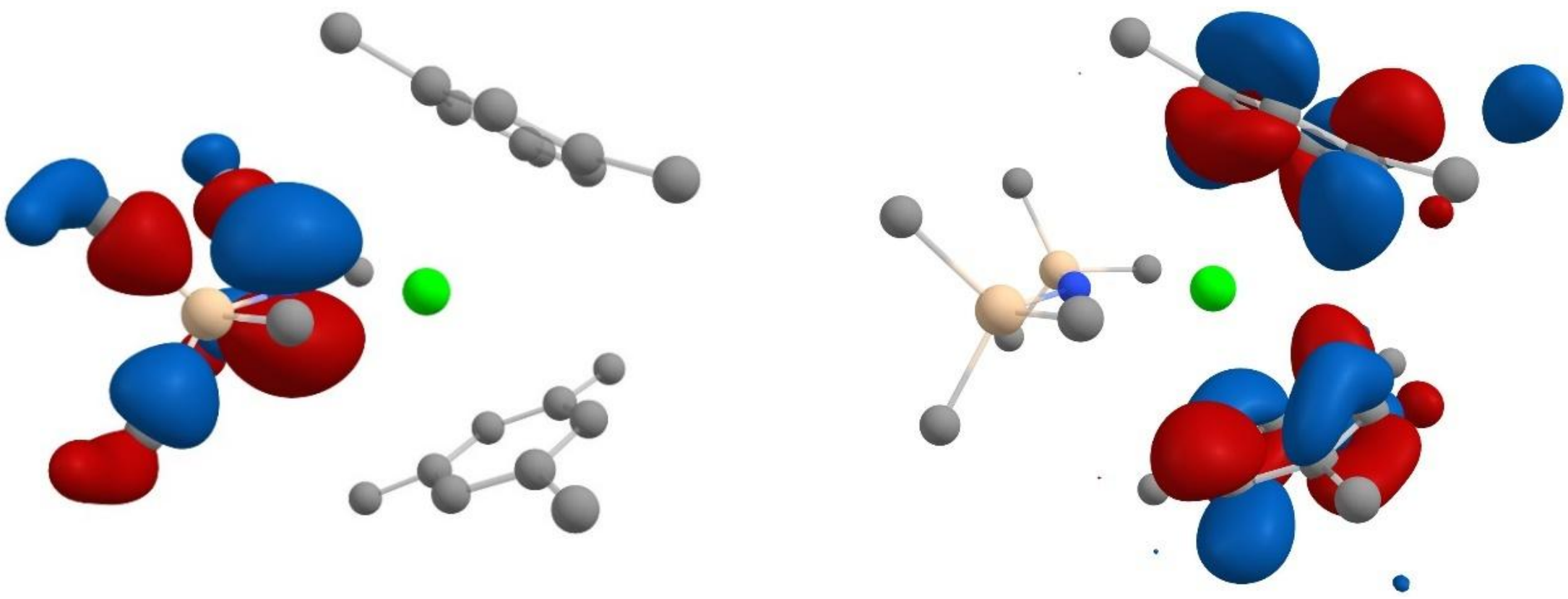

**HOMO**
MO-115 = -7.58 eV
-0.829*[41] LP (2) N 4(lp)
-0.226*[47] BD (1)Si 3- C21
0.226*[45] BD (1)Si 3- C21

**LUMO**
MO-116 = -5.26 eV
0.391*[134] BD*(2) C9- C14
-0.390*[158] BD*(2) C30- C31
-0.390*[132] BD*(2) C7- C46
0.389*[153] BD*(2) C27- C28
0.270*[52] BD (2) C5- C20
-0.269*[76] BD (2) C25- C33

**Figure S27** HOMO and LUMO for **5**$^{\mathbf{Mes}}$ (contour value = 0.03).

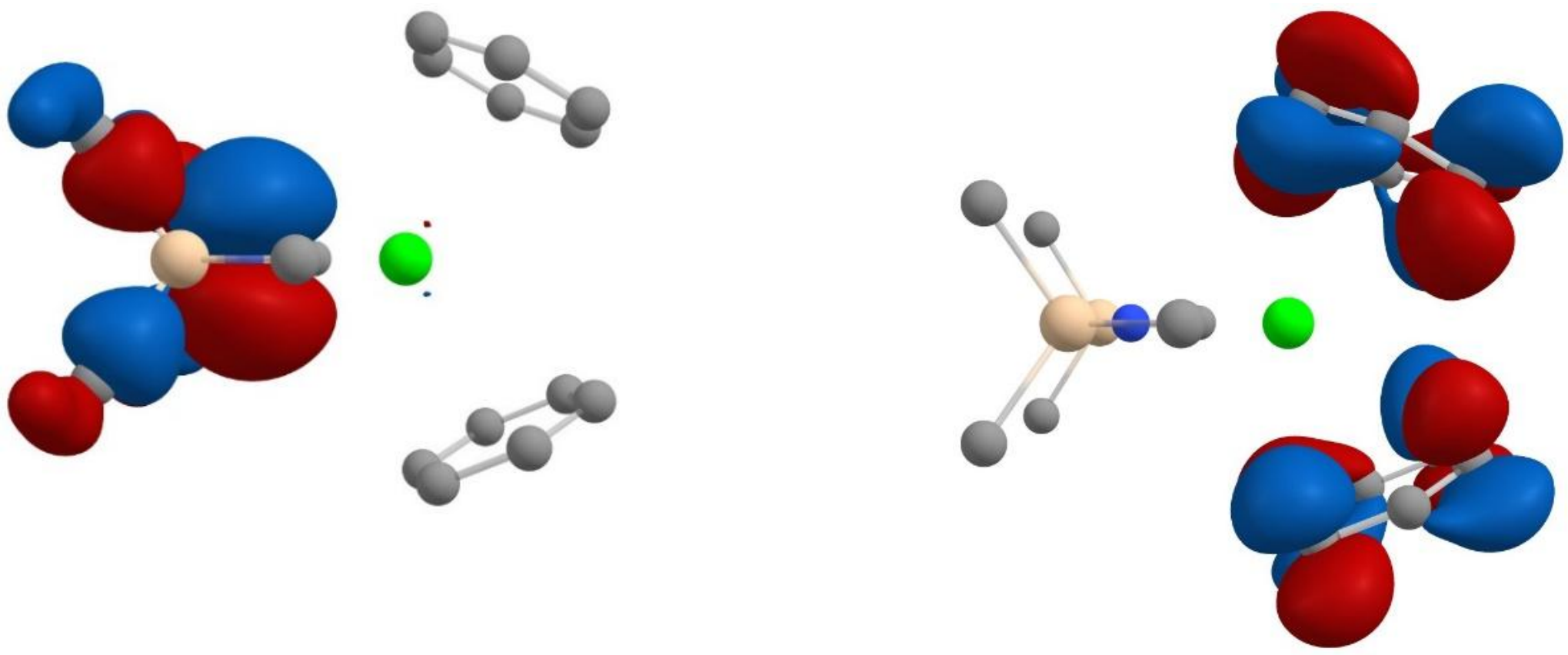

**HOMO**
MO-91 = -7.81 eV
-0.823*[35] LP (2) N 4(lp)

**LUMO**
MO-92 = -5.77 eV
0.461*[130] BD*(2) C33- C35
-0.458*[135] BD*(2) C38- C39
0.363*[146] BD*(2) C44- C45
-0.340*[121] BD*(2) C29- C36
0.258*[ 84] BD (2) C41- C42
-0.251*[ 68] BD (2) C30- C32

**Figure S28.** HOMO and LUMO for $\mathbf{5}^{\mathbf{Ben}}$ (contour value = 0.03).

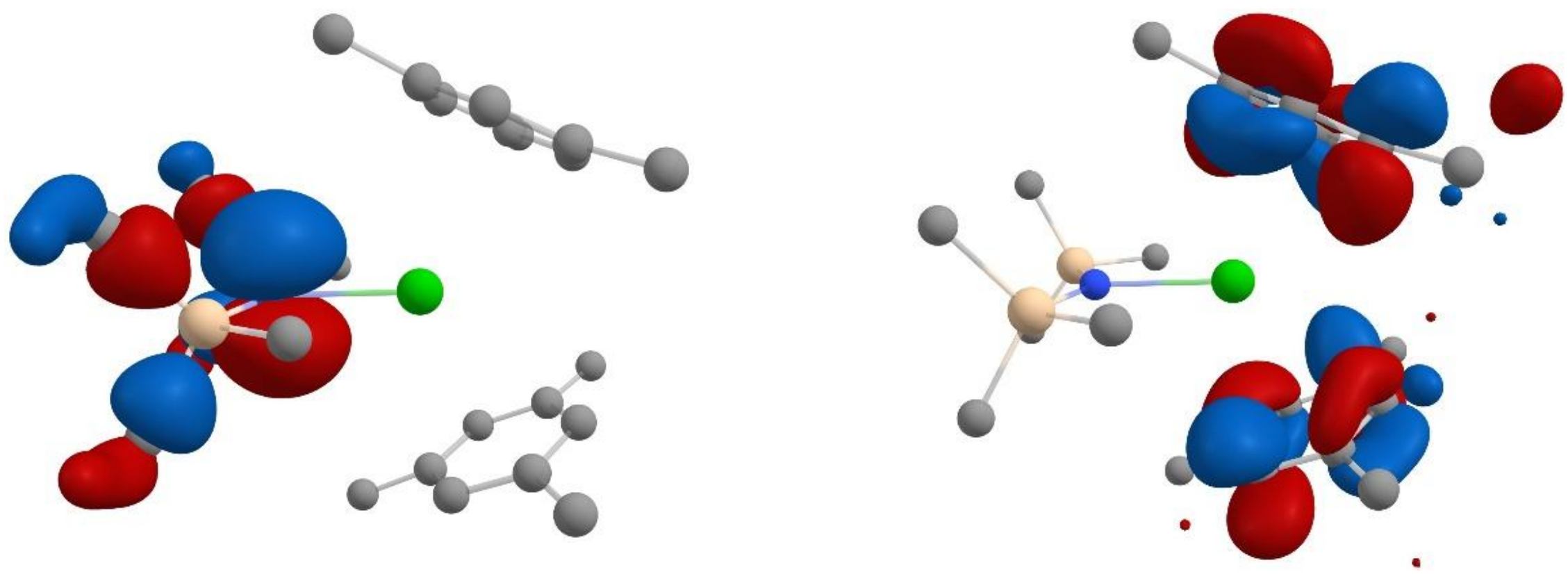

**HOMO**
MO-115 = -7.52 eV
-0.830*[41] LP (2) N 4(lp)

**LUMO**
MO-116 = -5.12 eV
0.392*[161] BD*(2) C32-C33
-0.389*[130] BD*(2) C 7-C 8
0.384*[135] BD*(2) C10-C11
-0.374*[150] BD*(2) C26-C27
0.266*[ 53] BD (2) C 5-C13
-0.264*[ 81] BD (2) C29-C30

**Figure S29.** HOMO and LUMO for $\mathbf{6}^{\mathbf{Mes}}$ (contour value = 0.03).

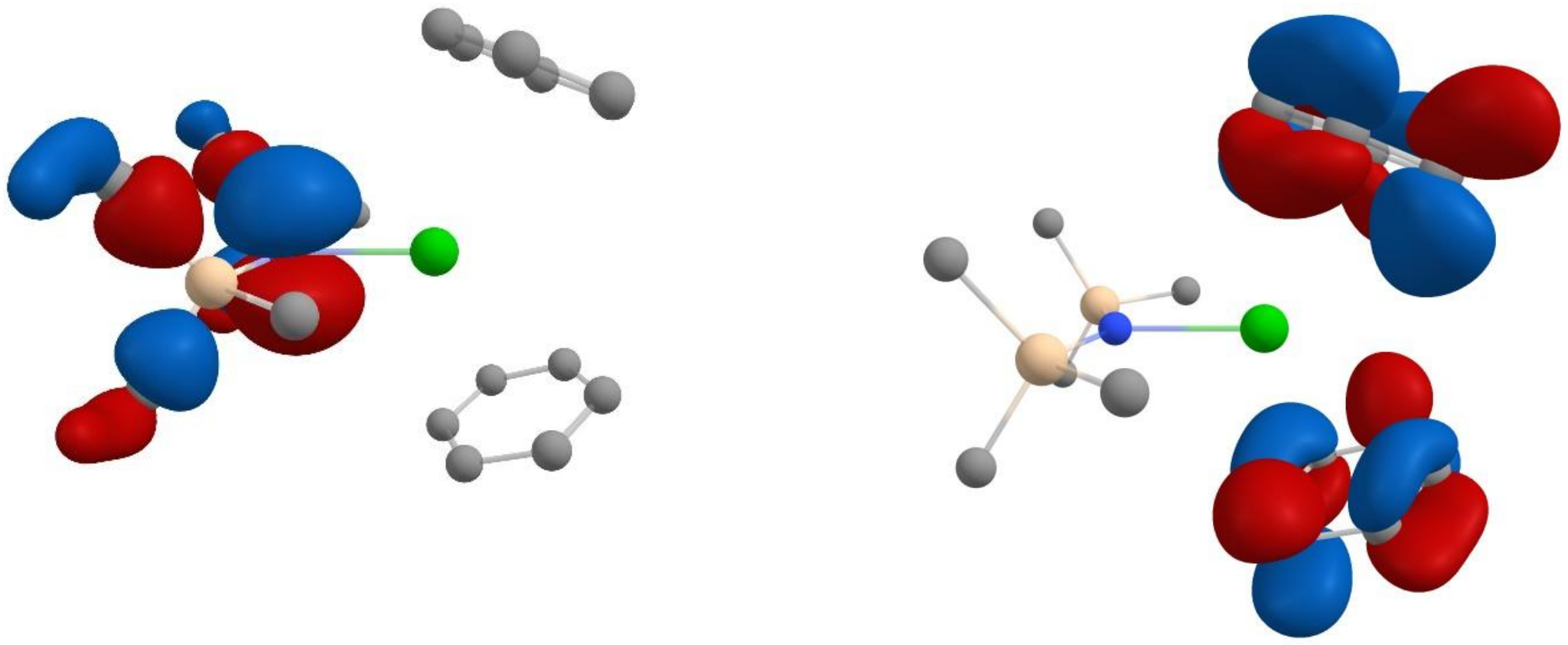

**HOMO**

MO-91 = -7.76 eV

0.824*[ 35] LP ( 2) N 4(lp)

**LUMO**

MO-92 = -5.59 eV

0.420*[126] BD*(2) C32- C33
-0.414*[140] BD*(2) C39- C41
-0.391*[145] BD*(2) C42- C44
0.387*[120] BD*(2) C29- C30
-0.280*[ 74] BD (2) C35- C36
0.279*[ 79] BD (2) C38- C45

**Figure S30.** HOMO and LUMO for **6**$^{\mathbf{Ben}}$ (contour value = 0.03).

**Table S11.** Selected NBO information for the Lone Vacancy (LV) of the alkali earth metal in species **1**$^{\mathbf{Ben}}$ – **6**$^{\mathbf{Ben}}$ and **1**$^{\mathbf{Mes}}$ – **6**$^{\mathbf{Mes}}$ and the dominant Atomic Orbital (AO) contributions.

| Structure | NBO | Occupancy | | *s* % | *p* % | Dominant *p* AO Contribution | *d* % | Dominant *d* AO Contribution |
|---|---|---|---|---|---|---|---|---|
| **1**$^{\mathbf{Ben}}$ | 151 | 0.09302 | LV Ca1 | 96.37 | 1.27 | $p_x$ (1.01%) | 2.37 | $d_{z^2}$ (1.49%) |
| | 152 | 0.09302 | LV Ca2 | 96.37 | 1.27 | $p_x$ (1.01%) | 2.37 | $d_{z^2}$ (1.49%) |
| **1**$^{\mathbf{Mes}}$ | 175 | 0.08864 | LV Ca1 | 96.43 | 1.01 | $p_x$ (0.73%) | 2.56 | $d_{xy}$ (1.29%), $d_{z^2}$ (1.12%) |
| | 176 | 0.08864 | LV Ca2 | 96.43 | 1.01 | $p_x$ (0.73%) | 2.56 | $d_{xy}$ (1.29%), $d_{z^2}$ (1.12%) |
| **2**$^{\mathbf{Ben}}$ | 141 | 0.09290 | LV Sr1 | 95.94 | 1.94 | $p_x$ (1.79%) | 2.11 | $d_{z^2}$ (1.27%) |
| | 142 | 0.09289 | LV Sr2 | 95.96 | 1.92 | $p_x$ (1.76%) | 2.10 | $d_{z^2}$ (1.27%) |
| **2**$^{\mathbf{Mes}}$ | 165 | 0.08578 | LV Sr1 | 97.05 | 1.15 | $p_x$ (0.95%) | 1.79 | $d_{z^2}$ (0.90%) |
| | 166 | 0.08578 | LV Sr2 | 97.05 | 1.15 | $p_x$ (0.95%) | 1.79 | $d_{z^2}$ (0.90%) |
| **3**$^{\mathbf{Ben}}$ | 141 | 0.08513 | LV Ba1 | 95.99 | 1.95 | $p_x$ (1.83%) | 1.97 | $d_{z^2}$ (1.24%) |
| | 142 | 0.08513 | LV Ba2 | 95.99 | 1.95 | $p_x$ (1.83%) | 1.97 | $d_{z^2}$ (1.24%) |
| **3**$^{\mathbf{Mes}}$ | 165 | 0.07995 | LV Ba1 | 97.15 | 1.18 | $p_x$ (1.04%) | 1.59 | $d_{z^2}$ (0.09%) |
| | 166 | 0.07995 | LV Ba2 | 97.15 | 1.18 | $p_x$ (1.04%) | 1.59 | $d_{z^2}$ (0.09%) |
| **4**$^{\mathbf{Mes}}$ | 121 | 0.09344 | LV Ca1 | 72.37 | 2.22 | $p_x$(2.16%) | 25.40 | $d_{x^2-y^2}$ (19.51%), $d_{z^2}$ (4.62%) |
| **4**$^{\mathbf{Ben}}$ | 97 | 0.10817 | LV Ca1 | 56.92 | 4.48 | $p_x$(4.26%) | 38.60 | $d_{xz}$*(3.14%),* $d_{x^2-y^2}$ (27.51%), $d_{z^2}$ (7.71%) |
| **5**$^{\mathbf{Mes}}$ | 116 | 0.08325 | LV Sr1 | 80.13 | 3.53 | $p_x$(3.52%) | 16.30 | $d_{x^2-y^2}$ (12.64%), $d_{z^2}$ (3.17%) |
| **5**$^{\mathbf{Ben}}$ | 92 | 0.09605 | LV Sr1 | 69.60 | 6.39 | $p_x$(6.36%) | 23.93 | $d_{x^2-y^2}$ (17.98%), $d_{z^2}$ (5.33%) |
| **6**$^{\mathbf{Mes}}$ | 116 | 0.07160 | LV Ba1 | 61.61 | 5.81 | $p_x$(5.76%) | 32.00 | $d_{x^2-y^2}$ (25.25%), $d_{z^2}$ (6.27%) |
| **6**$^{\mathbf{Ben}}$ | 92 | 0.04629 | LV Ba1 | 52.85 | 2.23 | $p_x$(2.03%) | 44.53 | $d_{x^2-y^2}$ (38.00%), $d_{z^2}$ (6.34%) |

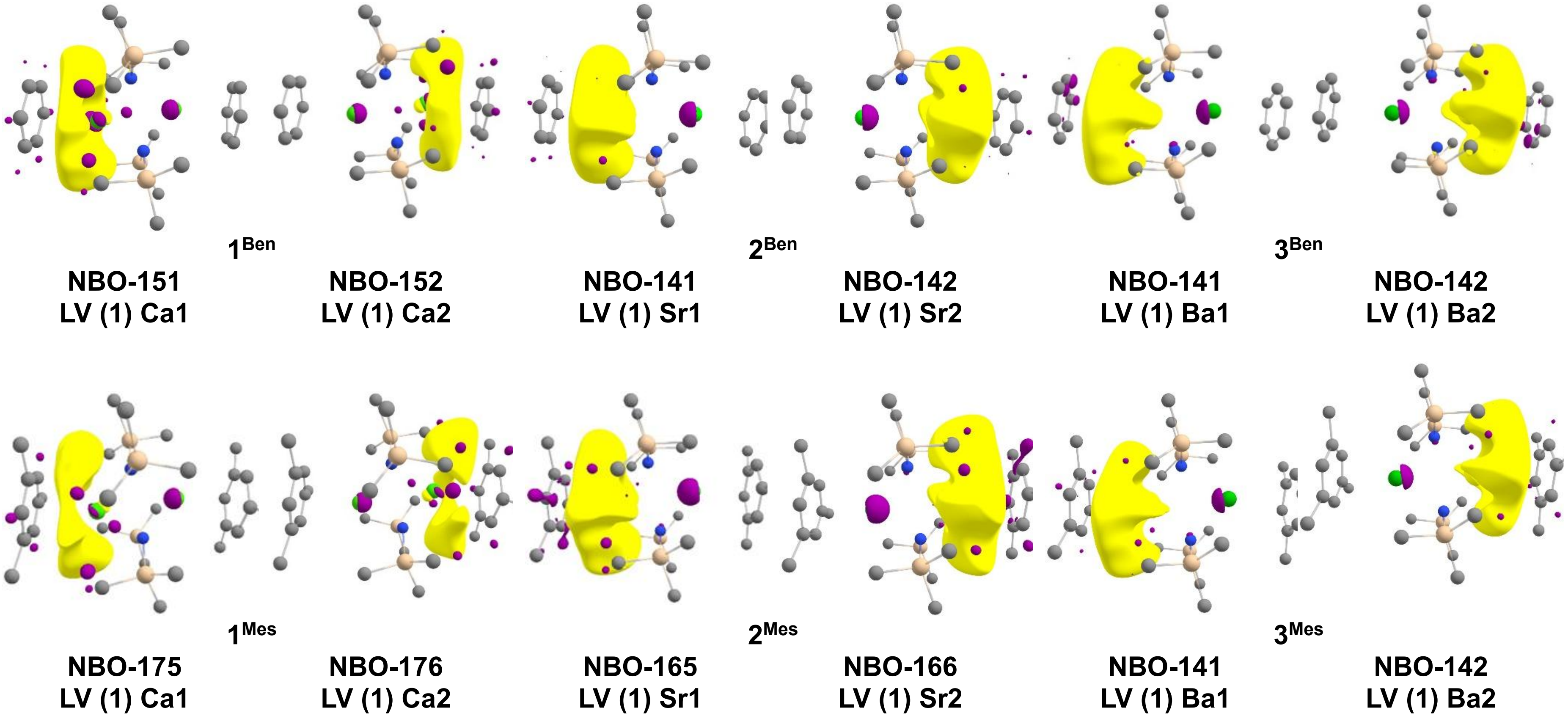


**Figure S31.** The alkali earth metal lone vacancy (LV) NBOs for species $\mathbf{1^{Ben}} – \mathbf{3^{Ben}}$ and $\mathbf{1^{Mes}} – \mathbf{3^{Mes}}$.

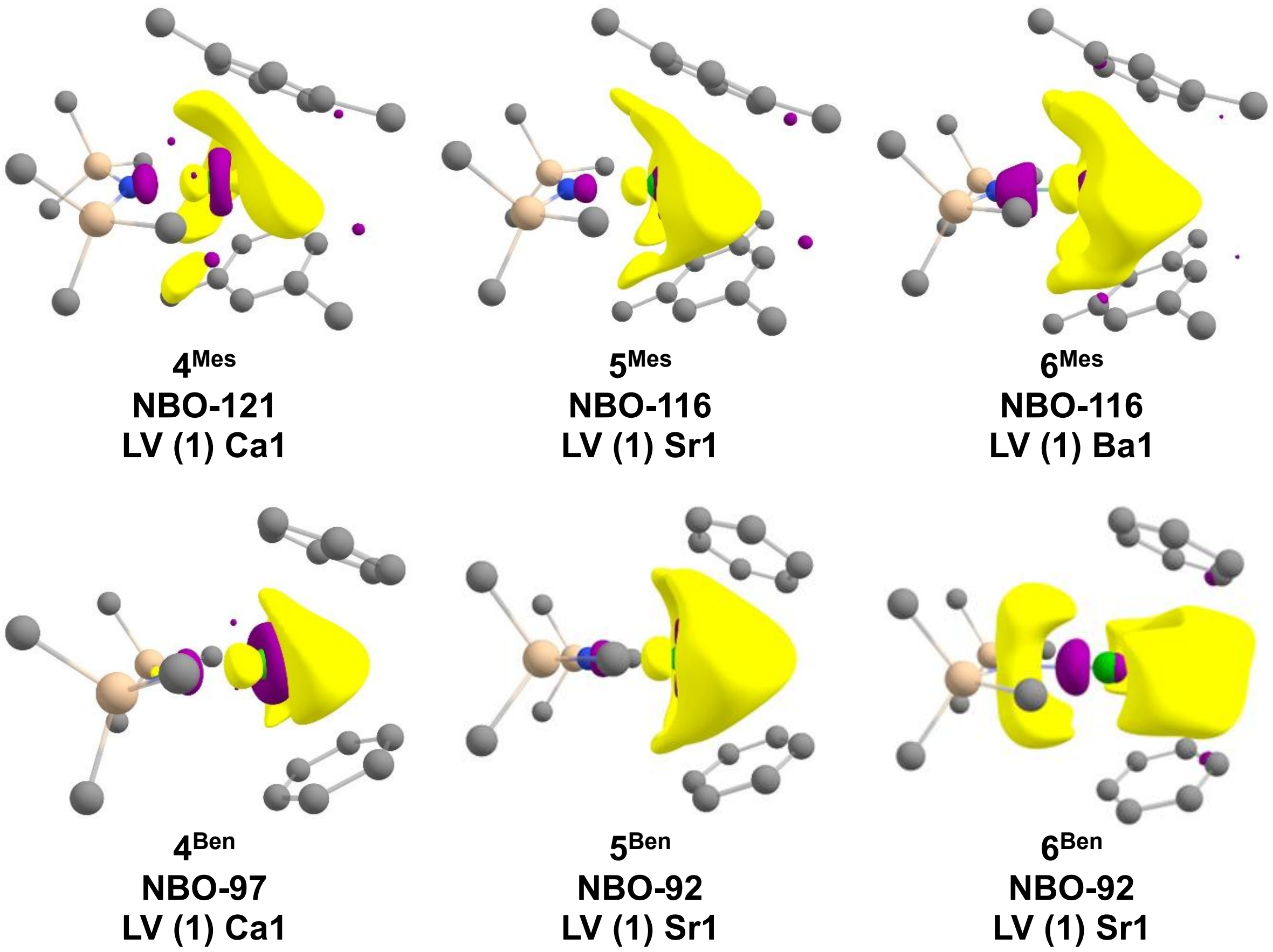


**Figure S32.** The alkali earth metal lone vacancy (LV) NBOs for species $\mathbf{4^{Mes}}$ – $\mathbf{6^{Mes}}$ and $\mathbf{4^{Ben}}$ – $\mathbf{6^{Ben}}$.

## EDA-NOCV analysis

Energy decomposition analysis with natural orbitals for chemical valence (EDA-NOCV) was performed to investigate the electronic contribution to the binding interaction between the Ae cation and a arene ligand in complexes $\mathbf{1}^{\mathbf{Ben}}$ – $\mathbf{6}^{\mathbf{Ben}}$, $\mathbf{1}^{\mathbf{Mes}}$ – $\mathbf{6}^{\mathbf{Mes}}$, Calculations were completed at the BP86-D3$^{BJ}$/BS3 level.

**Table S12.** EDA analysis of the Ae cation–arene interactions in complexes $\mathbf{1}^{\mathbf{Ben}}$–$\mathbf{3}^{\mathbf{Ben}}$ and $\mathbf{1}^{\mathbf{Mes}}$–$\mathbf{3}^{\mathbf{Mes}}$. Percentages given in parentheses represent the relative contribution of each energy component to the total attractive interaction.

| Complex | $\Delta E_{\text{int}}$ | $\Delta E_{\text{orb}}$ | $\Delta E_{\text{Elstat}}$ | $\Delta E_{\text{Pauli}}$ | $\Delta E^{0}_{XC}$ | $\Delta E_{D3}{}^{BJ}$ |
|---|---|---|---|---|---|---|
| $\mathbf{1}^{\mathbf{Ben}}$ | −34.8 | −23.5 (42.3%) | −17.8 (32.0%) | 30.7 | −10.0 | −14.3 (25.8%) |
| $\mathbf{1}^{\mathbf{Mes}}$ | −44.2 | −27.8 (40.1%) | −22.4 (32.2%) | 38.0 | −13.0 | −19.2 (27.7%) |
| $\mathbf{2}^{\mathbf{Ben}}$ | −31.5 | −20.8 (42.6%) | −15.3 (33.7%) | 25.1 | −7.0 | −11.6 (23.7%) |
| $\mathbf{2}^{\mathbf{Mes}}$ | −41.7 | −26.3 (41.2%) | −21.5 (33.6%) | 32.5 | −9.4 | −16.2 (25.3%) |
| $\mathbf{3}^{\mathbf{Ben}}$ | −27.8 | −17.5 (41.2%) | −14.8 (34.9%) | 21.0 | −4.6 | −10.2 (23.9%) |
| $\mathbf{3}^{\mathbf{Mes}}$ | −36.9 | −22.9 (40.6%) | −19.5 (35.0%) | 27.8 | −6.4 | −14.2 (25.0%) |

**Table S13.** EDA analysis of the Ae cation–arene interactions in complexes $\mathbf{4}^{\mathbf{Mes}}$–$\mathbf{6}^{\mathbf{Mes}}$ and $\mathbf{4}^{\mathbf{Ben}}$–$\mathbf{6}^{\mathbf{Ben}}$. Percentages given in parentheses represent the relative contribution of each energy component to the total attractive interaction.

| Complex | $\Delta E_{\text{int}}$ | $\Delta E_{\text{orb}}$ | $\Delta E_{\text{Elstat}}$ | $\Delta E_{\text{Pauli}}$ | $\Delta E^{0}_{XC}$ | $\Delta E_{D3}{}^{BJ}$ |
|---|---|---|---|---|---|---|
| $\mathbf{4}^{\mathbf{Mes}}$ | −38.7 | −24.5 (37.8%) | −21.9 (33.9%) | 39.1 | −13.1 | −18.3 (28.3%) |
| $\mathbf{4}^{\mathbf{Ben}}$ | −29.3 | −20.0 (40.5%) | −17.0 (34.4%) | 30.1 | −10.0 | −12.4 (25.1%) |
| $\mathbf{5}^{\mathbf{Mes}}$ | −35.3 | −21.0 (37.1%) | −19.5 (34.5%) | 31.1 | −9.3 | −16.0 (28.3%) |
| $\mathbf{5}^{\mathbf{Ben}}$ | −26.3 | −16.9 (39.9%) | −15.0 (35.5%) | 23.3 | −6.9 | −10.4 (24.6%) |
| $\mathbf{6}^{\mathbf{Mes}}$ | −30.9 | −17.6 (36.1%) | −17.1 (35.2%) | 25.2 | −6.5 | −14.0 (28.7%) |
| $\mathbf{6}^{\mathbf{Ben}}$ | −23.4 | −14.0 (38.1%) | −13.4 (36.4%) | 19.2 | −5.0 | −9.4 (25.5%) |

## References


1. M. Westerhausen and W. Schwarz, *Z. Anorg. Allg. Chem.*, 1991, **604**, 127-140.
2. M. Westerhausen and W. Schwarz, *Z. Anorg. Allg. Chem.*, 1991, **604**, 177-190.
3. B. A. Vaartstra, J. C. Huffman, W. E. Streib and K. G. Caulton, *Inorg. Chem.*, 1991, **30**, 121-125.
4. I. Krossing, H. Brands, R. Fueuerhake and S. Koenig, *J. Flourine Chem.*, 2001, **112**, 83-90.
5. O. V. Dolomanov, L. J. Bourhis, R. J. Gildea, J. A. K. Howard and H. Puschmann, *J. Appl. Cryst.,* 2009, **42**, 339-341
6. G. M. Sheldrick, *Acta Cryst.,* 2015, A71, 3-8.
7. G. M. Sheldrick, *Acta Cryst.,* 2015, C71, 3-8.
8. D. Kratzert, J. J. Holstein and I. Krossing, *J. Appl. Cryst.*, 2015, **48**, 933-938.
9. M. J. Frisch, G. W. Trucks, H. B. Schlegel, G. E. Scuseria, M. A. Robb, J. R. Cheeseman, G. Scalmani, V. Barone, G. A. Petersson, H. Nakatsuji, X. Li, M. Caricato, A. V. Marenich, J. Bloino, B. G. Janesko, R. Gomperts, B. Mennucci, H. P. Hratchian, J. V. Ortiz, A. F. Izmaylov, J. L. Sonnenberg, Williams, F. Ding, F. Lipparini, F. Egidi, J. Goings, B. Peng, A. Petrone, T. Henderson, D. Ranasinghe, V. G. Zakrzewski, J. Gao, N. Rega, G. Zheng, W. Liang, M. Hada, M. Ehara, K. Toyota, R. Fukuda, J. Hasegawa, M. Ishida, T. Nakajima, Y. Honda, O. Kitao, H. Nakai, T. Vreven, K. Throssell, J. A. Montgomery Jr., J. E. Peralta, F. Ogliaro, M. J. Bearpark, J. J. Heyd, E. N. Brothers, K. N. Kudin, V. N. Staroverov, T. A. Keith, R. Kobayashi, J. Normand, K. Raghavachari, A. P. Rendell, J. C. Burant, S. S. Iyengar, J. Tomasi, M. Cossi, J. M. Millam, M. Klene, C. Adamo, R. Cammi, J. W. Ochterski, R. L. Martin, K. Morokuma, O. Farkas, J. B. Foresman, D. J. Fox. Gaussian 16 (Rev C.01), Gaussian Inc, Wallingford, CT, 2016.
10. a) A. D. Becke, *Phys. Rev. A* 1988, **38**, 3098-3100; b) J. P. Perdew, *Phys. Rev. B* 1986, **33**, 8822 882.
11. F. Weigend; R. Ahlrichs, *Phys. Chem. Chem. Phys.* 2005, **7**, 3297-330.
12. S. Grimme, S. Ehrlich, L. Goerigk, *J. Comp. Chem.* 2011, **32**, 1456-1465.
13. V. Barone, M. J. Cossi, *Phys. Chem. A* 1998, **102**, 1995–2001.
14. P. Pollak, F. Weigend, *J. Chem. Theory Comput.* 2017, **13**, 3696–3705.
15. J. D. Rolfes, F. Neese, D. A. Pantazis, *J. Comput. Chem.* 2020, **41**, 1842–1849.
16. F. Neese, *WIREs Comput. Mol. Sci.* 2024, **14**, e1706.
17. E. D. Glendening, J. K. Badenhoop, A. E. Reed, J. E. Carpenter, J. A. Bohmann, C. M. Morales, P. Karafiloglou, C. R. Landis, and F. Weinhold. NBO 7.0, Theoretical Chemistry Institute, University of Wisconsin, Madison, USA, 2003.
18. G. Zhurko, D. Zhurko, *Chemcraft*, http://www.chemcraftprog.com.
19. M. Mitoraj, A. Michalak, T. Ziegler, *J. Chem. Theory Comput.* 2009, **5**, 962–975.

## Raw Computed Energies (in Hartrees) and Cartesian Coordinates

**1[Ben]**

SCF (BP86) Energy = -3564.98249713
Enthalpy 0K = -3564.340259
Enthalpy 298K = -3564.289539
Free Energy 298K = -3564.424189
Lowest Frequency = 20.9519 $cm^{-1}$
Second Frequency = 21.9312 $cm^{-1}$
SCF (Benzene) Energy = -3565.10275011
SCF (BP86-D3[BJ]) Energy = -3565.21973458
SCF (BS2) Energy = -3566.73373923
SCF (BS3) Energy = -3580.90372052

```
Ca -1.67033  0.47053  0.12812
Ca  1.67078 -0.47033 -0.12845
Si  0.77931  2.22433 -1.65659
Si  0.41141  2.60710  1.43271
Si -0.41091 -2.60691 -1.43294
Si -0.77963 -2.22420  1.65624
N   0.49223  1.56325 -0.02090
N  -0.49179 -1.56308  0.02070
C  -4.17846  1.73323 -1.12087
H  -4.03373  2.61215 -1.76811
C  -4.30897 -0.69417 -0.84507
H  -4.26537 -1.70707 -1.27408
C  -4.66195 -0.51963  0.51006
H  -4.88822 -1.39686  1.13637
C  -4.76810  0.77950  1.05088
H  -5.07325  0.91651  2.10053
C  -4.52739  1.90565  0.23566
H  -4.64746  2.91897  0.64942
C  -4.06805  0.43271 -1.66218
H  -3.84186  0.29918 -2.73195
C  -0.86307  2.64697 -2.51651
H  -1.55074  1.77845 -2.62769
H  -0.67739  3.01855 -3.54699
H  -1.40486  3.45376 -1.97790
C   1.57461  0.80303 -2.67926
H   0.96627 -0.12220 -2.77461
H   2.61559  0.53493 -2.38105
H   1.68728  1.16989 -3.72199
C   1.95179  3.69145 -1.83210
H   1.50581  4.64637 -1.49254
H   2.19896  3.81298 -2.90872
H   2.90636  3.54937 -1.28535
C   1.10730  4.35547  1.30751
H   2.18022  4.37494  1.03021
H   1.01941  4.82711  2.31023
H   0.55371  4.99938  0.59466
C   1.27693  1.76225  2.90149
H   1.12957  0.66327  2.97200
H   0.91464  2.19210  3.85983
H   2.36942  1.95604  2.86780
C  -1.44759  2.86137  1.87069
H  -1.98208  3.40116  1.05798
H  -1.49473  3.53014  2.75743
H  -2.06448  1.98551  2.18017
C  -1.57462 -0.80261  2.67885
H  -0.96544  0.12197  2.77510
H  -1.68850 -1.16991  3.72128
H  -2.61511 -0.53323  2.38001
C   0.86214 -2.64769  2.51683
H   1.40366 -3.45489  1.97853
H   0.67578 -3.01905  3.54728
H   1.55027 -1.77956  2.62813
C  -1.95298 -3.69073  1.83109
H  -2.90698 -3.54832  1.28345
H  -2.20118 -3.81188  2.90753
H  -1.50717 -4.64595  1.49217
C   1.44808 -2.86093 -1.87117
H   2.06502 -1.98504 -2.18049
H   1.49508 -3.52944 -2.75811
H   1.98267 -3.40098 -1.05870
C  -1.10642 -4.35542 -1.30764
H  -0.55281 -4.99914 -0.59464
H  -1.01834 -4.82716 -2.31029
H  -2.17937 -4.37504 -1.03048
C  -1.27673 -1.76218 -2.90158
H  -2.36923 -1.95588 -2.86755
H  -0.91476 -2.19206 -3.86002
H  -1.12930 -0.66320 -2.97215
C   4.17803 -1.73208  1.12374
H   4.03311 -2.60975  1.77262
C   4.52663 -1.90711 -0.23256
H   4.64622 -2.92123 -0.64448
C   4.76759 -0.78253 -1.04990
H   5.07250 -0.92154 -2.09945
C   4.66202  0.51759 -0.51133
H   4.88857  1.39355 -1.13918
C   4.30933  0.69474  0.84352
H   4.26616  1.70846  1.27065
C   4.06815 -0.43056  1.66270
H   3.84208 -0.29499  2.73224
```

**1[Mes]**

SCF (BP86) Energy = -3800.71591762
Enthalpy 0K = -3799.915009
Enthalpy 298K = -3799.853784
Free Energy 298K = -3800.008667
Lowest Frequency = 19.3482 $cm^{-1}$
Second Frequency = 23.5387 $cm^{-1}$
SCF (Mesitylene) Energy = -3800.82628301
SCF (BP86-D3[BJ]) Energy = -3801.00009575
SCF (BS2) Energy = -3802.72237366
SCF (BS3) Energy = -3817.09026924

```
Ca -1.77193 -0.26142 -0.07140
Ca  1.77191  0.26136  0.07138
Si  0.26106 -2.10643  1.74602
Si  0.25150 -2.72557 -1.33178
Si -0.25143  2.72554  1.33175
Si -0.26112  2.10638 -1.74603
N   0.30702 -1.58726  0.04764
N  -0.30700  1.58720 -0.04764
C  -4.66539 -1.36132  0.63124
H  -4.79666 -2.35324  1.09526
C  -4.40819  1.04784  0.85249
H  -4.34766  1.94731  1.48760
C  -4.48654  1.20599 -0.55400
C  -4.63022  0.04395 -1.35235
H  -4.73741  0.15215 -2.44477
C  -4.74989 -1.24715 -0.77856
C  -4.53551 -0.22346  1.46694
```

| | | | |
|---|---|---|---|
| C | -1.50997 | -1.85944 | 2.42930 |
| H | -1.88910 | -0.81177 | 2.46498 |
| H | -1.53368 | -2.18390 | 3.49178 |
| H | -2.25542 | -2.49354 | 1.90092 |
| C | 1.38362 | -0.91521 | 2.74015 |
| H | 1.13498 | 0.16662 | 2.65098 |
| H | 2.46385 | -1.05725 | 2.52455 |
| H | 1.26946 | -1.13192 | 3.82374 |
| C | 0.76362 | -3.86337 | 2.20580 |
| H | 0.04108 | -4.62047 | 1.84026 |
| H | 0.78484 | -3.93968 | 3.31433 |
| H | 1.76856 | -4.14175 | 1.83226 |
| C | 1.00495 | -4.43870 | -1.06924 |
| H | 2.05866 | -4.39219 | -0.72433 |
| H | 1.00581 | -4.96794 | -2.04638 |
| H | 0.44026 | -5.07081 | -0.35666 |
| C | 1.15098 | -1.97920 | -2.83201 |
| H | 0.73600 | -1.02089 | -3.20315 |
| H | 1.09536 | -2.69619 | -3.67897 |
| H | 2.23215 | -1.83058 | -2.62264 |
| C | -1.58436 | -2.98920 | -1.79874 |
| H | -2.14239 | -3.48088 | -0.97268 |
| H | -1.66417 | -3.66390 | -2.67806 |
| H | -2.14765 | -2.07405 | -2.09845 |
| C | -1.38363 | 0.91508 | -2.74011 |
| H | -1.13486 | -0.16672 | -2.65099 |
| H | -1.26958 | 1.13184 | -3.82370 |
| H | -2.46387 | 1.05698 | -2.52443 |
| C | 1.50990 | 1.85945 | -2.42936 |
| H | 2.25536 | 2.49357 | -1.90102 |
| H | 1.53355 | 2.18392 | -3.49184 |
| H | 1.88906 | 0.81179 | -2.46505 |
| C | -0.76379 | 3.86329 | -2.20581 |
| H | -1.76872 | 4.14161 | -1.83221 |
| H | -0.78507 | 3.93958 | -3.31433 |
| H | -0.04127 | 4.62044 | -1.84031 |
| C | 1.58445 | 2.98912 | 1.79865 |
| H | 2.14768 | 2.07396 | 2.09843 |
| H | 1.66431 | 3.66389 | 2.67790 |
| H | 2.14251 | 3.48069 | 0.97254 |
| C | -1.00484 | 4.43869 | 1.06923 |
| H | -0.44013 | 5.07076 | 0.35663 |
| H | -1.00562 | 4.96793 | 2.04637 |
| H | -2.05856 | 4.39223 | 0.72435 |
| C | -1.15088 | 1.97920 | 2.83202 |
| H | -2.23205 | 1.83061 | 2.62264 |
| H | -1.09523 | 2.69619 | 3.67897 |
| H | -0.73593 | 1.02088 | 3.20316 |
| C | 4.66533 | 1.36147 | -0.63106 |
| H | 4.79657 | 2.35345 | -1.09496 |
| C | 4.74987 | 1.24715 | 0.77872 |
| C | 4.63021 | -0.04403 | 1.35237 |
| H | 4.73740 | -0.15235 | 2.44478 |
| C | 4.48653 | -1.20598 | 0.55390 |
| C | 4.40823 | -1.04767 | -0.85259 |
| H | 4.34775 | -1.94707 | -1.48780 |
| C | 4.53551 | 0.22370 | -1.46689 |
| C | 5.09077 | 2.44305 | 1.63447 |
| H | 4.68743 | 2.35782 | 2.66243 |
| H | 6.19645 | 2.51848 | 1.72922 |
| H | 4.74210 | 3.39499 | 1.18938 |
| C | 4.53986 | -2.58362 | 1.17025 |
| H | 5.59093 | -2.94653 | 1.17094 |
| H | 4.20215 | -2.59206 | 2.22541 |
| H | 3.94747 | -3.32269 | 0.59561 |
| C | 4.65701 | 0.34660 | -2.96698 |
| H | 5.71619 | 0.17802 | -3.26123 |
| H | 4.05465 | -0.41143 | -3.50639 |
| H | 4.37845 | 1.35386 | -3.33196 |
| C | -5.09079 | -2.44316 | -1.63417 |
| H | -6.19646 | -2.51871 | -1.72878 |
| H | -4.68755 | -2.35800 | -2.66218 |
| H | -4.74199 | -3.39504 | -1.18902 |
| C | -4.53988 | 2.58356 | -1.17052 |
| H | -4.20176 | 2.59197 | -2.22555 |
| H | -5.59102 | 2.94626 | -1.17167 |
| H | -3.94786 | 3.32280 | -0.59572 |
| C | -4.65699 | -0.34617 | 2.96705 |
| H | -4.05451 | 0.41182 | 3.50636 |
| H | -5.71614 | -0.17741 | 3.26130 |
| H | -4.37858 | -1.35344 | 3.33212 |

**2^Ben**

SCF (BP86) Energy = -2271.34163285
Enthalpy 0K = -2270.702329
Enthalpy 298K = -2270.650044
Free Energy 298K = -2270.794288
Lowest Frequency = 6.7052 $cm^{-1}$
Second Frequency = 7.0347 $cm^{-1}$
SCF (Benzene) Energy = -2271.46161069
SCF (BP86-D3$^{BJ}$) Energy = -2271.57171633
SCF (BS2) Energy = -2272.94896357
SCF (BS3) Energy = -8627.27224602

| | | | |
|---|---|---|---|
| Sr | -1.87913 | 0.24805 | -0.00226 |
| Sr | 1.87951 | -0.24648 | 0.00065 |
| Si | 0.26709 | 2.51956 | -1.56694 |
| Si | 0.26289 | 2.49255 | 1.59003 |
| Si | -0.27132 | -2.49409 | -1.58245 |
| Si | -0.25762 | -2.51791 | 1.57387 |
| N | 0.26928 | 1.69326 | 0.00488 |
| N | -0.26898 | -1.69231 | 0.00131 |
| C | -4.83463 | 1.22824 | -0.96180 |
| H | -4.89327 | 2.03392 | -1.71066 |
| C | -4.80628 | -1.15673 | -0.41843 |
| H | -4.84169 | -2.20917 | -0.74230 |
| C | -4.80254 | -0.84248 | 0.95791 |
| H | -4.84307 | -1.65033 | 1.70587 |
| C | -4.80861 | 0.50680 | 1.37497 |
| H | -4.84607 | 0.75153 | 2.44864 |
| C | -4.82489 | 1.54211 | 0.41458 |
| H | -4.87608 | 2.59347 | 0.74003 |
| C | -4.82319 | -0.12105 | -1.37820 |
| H | -4.87466 | -0.36379 | -2.45169 |
| C | -1.51578 | 2.54650 | -2.27674 |
| H | -1.99359 | 1.56007 | -2.48526 |
| H | -1.51053 | 3.05515 | -3.26528 |
| H | -2.19941 | 3.14150 | -1.63090 |
| C | 1.35721 | 1.49202 | -2.75670 |

H 1.04801 0.43116 -2.88397
H 2.43209 1.52185 -2.46540
H 1.32203 1.93016 -3.77744
C 0.90117 4.29414 -1.64831
H 0.24624 5.01409 -1.11839
H 0.93674 4.60662 -2.71417
H 1.92621 4.40199 -1.23835
C 0.86134 4.27765 1.70105
H 1.88272 4.41442 1.29096
H 0.89255 4.57196 2.77223
H 0.18979 4.99282 1.18531
C 1.37875 1.46287 2.75425
H 1.08871 0.39437 2.86367
H 1.34295 1.87963 3.78384
H 2.45067 1.51725 2.45601
C -1.51540 2.47451 2.31307
H -2.21469 3.07080 1.68548
H -1.50976 2.96503 3.31074
H -1.97525 1.47705 2.50832
C -1.34436 -1.49404 2.76866
H -1.01992 -0.44023 2.91232
H -1.32529 -1.94767 3.78299
H -2.41707 -1.50266 2.46755
C 1.52806 -2.53648 2.27750
H 2.21305 -3.12830 1.63036
H 1.52777 -3.04520 3.26602
H 2.00195 -1.54797 2.48530
C -0.88101 -4.29621 1.65630
H -1.90855 -4.40967 1.25432
H -0.90627 -4.61085 2.72182
H -0.22570 -5.01119 1.12005
C 1.50646 -2.50022 -2.30549
H 1.97900 -1.50876 -2.50160
H 1.49604 -2.99271 -3.30213
H 2.19715 -3.10315 -1.67480
C -0.89732 -4.26982 -1.69255
H -0.23452 -4.99611 -1.18122
H -0.93917 -4.56233 -2.76385
H -1.91849 -4.39077 -1.27685
C -1.37402 -1.45072 -2.74767
H -2.44770 -1.49584 -2.45385
H -1.33904 -1.86639 -3.77774
H -1.07368 -0.38482 -2.85515
C 4.83819 -1.44998 0.63405
H 4.90053 -2.42733 1.13845
C 4.83265 -1.38187 -0.77590
H 4.89083 -2.30588 -1.37275
C 4.80715 -0.12652 -1.42221
H 4.84446 -0.07445 -2.52226
C 4.79292 1.06100 -0.65795
H 4.82206 2.04091 -1.16074
C 4.79975 0.99277 0.75212
H 4.83580 1.91971 1.34655
C 4.81928 -0.26272 1.39851
H 4.86535 -0.31632 2.49816

**$2^{Mes}$**

SCF (BP86) Energy = -2507.07848788
Enthalpy 0K = -2506.280070
Enthalpy 298K = -2506.217539
Free Energy 298K = -2506.379614
Lowest Frequency = 11.0223 $cm^{-1}$
Second Frequency = 12.1690 $cm^{-1}$
SCF (Mesitylene) Energy = -2507.19041973
SCF (BP86-$D3^{BJ}$) Energy = -2507.35274411
SCF (BS2) Energy = -2508.93981140
SCF (BS3) Energy = -8863.45857448

Sr 1.91088 -0.21682 0.03082
Sr -1.91089 0.21686 -0.03082
Si -0.21967 -2.48177 -1.59133
Si -0.21909 -2.53218 1.55785
Si 0.21914 2.53219 -1.55785
Si 0.21960 2.48180 1.59135
N -0.24877 -1.69569 -0.00434
N 0.24874 1.69572 0.00436
C 4.86870 -1.40501 -0.42722
H 4.97783 -2.45488 -0.74790
C 4.70411 0.95846 -0.98137
H 4.69671 1.76383 -1.73519
C 4.72728 1.30523 0.39277
C 4.80449 0.25920 1.34667
H 4.86771 0.51355 2.41837
C 4.91127 -1.10027 0.95648
C 4.79532 -0.38903 -1.41369
C 1.56041 -2.43646 -2.30525
H 1.99211 -1.42738 -2.50183
H 1.57296 -2.93354 -3.29944
H 2.27117 -3.00794 -1.66747
C -1.32764 -1.45438 -2.76659
H -1.01460 -0.39451 -2.89174
H -2.40083 -1.47915 -2.46818
H -1.30149 -1.88879 -3.78906
C -0.79518 -4.27464 -1.72414
H -0.11350 -4.98536 -1.21557
H -0.82195 -4.55822 -2.79829
H -1.81386 -4.43031 -1.31480
C -0.78582 -4.33203 1.62401
H -1.80754 -4.47457 1.21649
H -0.80561 -4.65699 2.68651
H -0.10670 -5.02119 1.08354
C -1.32938 -1.56136 2.77211
H -1.04414 -0.49643 2.91572
H -1.28226 -2.02073 3.78284
H -2.40061 -1.60678 2.47290
C 1.56636 -2.50888 2.26122
H 2.27379 -3.04941 1.59406
H 1.58825 -3.05144 3.23112
H 1.99904 -1.50954 2.50288
C 1.32762 1.45451 2.76661
H 1.01463 0.39463 2.89179
H 1.30150 1.88894 3.78907
H 2.40081 1.47931 2.46818
C -1.56048 2.43635 2.30526
H -2.27130 3.00776 1.66749
H -1.57306 2.93345 3.29945
H -1.99209 1.42724 2.50189
C 0.79496 4.27472 1.72413

H   1.81364  4.43046  1.31481
H   0.82167  4.55833  2.79827
H   0.11322  4.98538  1.21551
C  -1.56630  2.50900 -2.26124
H  -1.99906  1.50967 -2.50285
H  -1.58813  3.05151 -3.23117
H  -2.27369  3.04961 -1.59411
C   0.78602  4.33198 -1.62404
H   0.10694  5.02122 -1.08361
H   0.80588  4.65691 -2.68655
H   1.80772  4.47445 -1.21648
C   1.32941  1.56127 -2.77205
H   2.40063  1.60666 -2.47284
H   1.28230  2.02058 -3.78281
H   1.04412  0.49635 -2.91563
C  -4.86868  1.40492  0.42739
H  -4.97779  2.45474  0.74820
C  -4.91125  1.10035 -0.95635
C  -4.80448 -0.25905 -1.34673
H  -4.86771 -0.51326 -2.41846
C  -4.72728 -1.30523 -0.39296
C  -4.70409 -0.95862  0.98121
H  -4.69668 -1.76411  1.73494
C  -4.79528  0.38880  1.41373
C  -5.18109  2.17776 -1.97959
H  -4.70117  1.96610 -2.95541
H  -6.27554  2.23657 -2.16786
H  -4.86330  3.18017 -1.63179
C  -4.77674 -2.75296 -0.82242
H  -5.83242 -3.10205 -0.83111
H  -4.38380 -2.90409 -1.84718
H  -4.22809 -3.41667 -0.12504
C  -4.92092  0.72097  2.88220
H  -5.98188  0.61105  3.19618
H  -4.32955  0.03648  3.52313
H  -4.62620  1.76499  3.10424
C   5.18113 -2.17753  1.97987
H   6.27559 -2.23626  2.16818
H   4.70117 -1.96577  2.95564
H   4.86342 -3.18000  1.63217
C   4.77671  2.75302  0.82203
H   4.38371  2.90430  1.84676
H   5.83239  3.10214  0.83074
H   4.22809  3.41663  0.12454
C   4.92096 -0.72138 -2.88212
H   4.32960 -0.03698 -3.52315
H   5.98193 -0.61150 -3.19610
H   4.62624 -1.76543 -3.10404

$\mathbf{3}^{\mathbf{Ben}}$

SCF (BP86) Energy = -2260.91154937
Enthalpy 0K = -2260.272665
Enthalpy 298K = -2260.220587
Free Energy 298K = -2260.362888
Lowest Frequency = 13.7186 cm$^{-1}$
Second Frequency = 14.3378 cm$^{-1}$
SCF (Benzene) Energy = -2261.04427602
SCF (BP86-D3$^{BJ}$) Energy = -2261.13844982
SCF (BS2) Energy = -2262.55931637
SCF (BS3) Energy = -18768.03477163

Ba -2.04908 -0.23145 -0.00078
Ba  2.04905  0.23121 -0.00094
Si  0.24839 -2.58102  1.57949
Si  0.24598 -2.58145 -1.58129
Si -0.24856  2.58050  1.57966
Si -0.24587  2.58149 -1.58113
N   0.24774 -1.78443 -0.00102
N  -0.24767  1.78418 -0.00099
C  -5.19088 -0.94785 -1.14924
H  -5.24653 -1.58293 -2.04801
C  -5.16399  0.45803 -1.27987
H  -5.20249  0.91731 -2.28083
C  -5.14738  1.27438 -0.12820
H  -5.16772  2.37136 -0.23019
C  -5.15695  0.68471  1.15427
H  -5.19126  1.32134  2.05305
C  -5.18198 -0.72116  1.28553
H  -5.23038 -1.17989  2.28632
C  -5.19972 -1.53720  0.13356
H  -5.26198 -2.63273  0.23583
C   0.83005 -4.37392  1.67581
H   0.14607 -5.07787  1.16074
H   0.87055 -4.67800  2.74383
H   1.84518 -4.51689  1.25231
C  -1.50959 -2.54342  2.36010
H  -1.90420 -1.54581  2.66509
H  -1.48786 -3.12334  3.30824
H  -2.26521 -3.05293  1.71942
C   1.38382 -1.57820  2.75761
H   1.09775 -0.51135  2.89959
H   2.46059 -1.63405  2.47241
H   1.33839 -2.02345  3.77487
C  -1.51235 -2.53956 -2.36139
H  -2.26919 -3.04694 -1.72036
H  -1.49209 -3.12034 -3.30903
H  -1.90455 -1.54130 -2.66742
C   0.82175 -4.37625 -1.67725
H   1.83631 -4.52268 -1.25363
H   0.86127 -4.68063 -2.74522
H   0.13527 -5.07777 -1.16213
C   1.38349 -1.58217 -2.76029
H   2.46034 -1.64192 -2.47627
H   1.10080 -0.51427 -2.90139
H   1.33526 -2.02668 -3.77776
C   1.50934  2.54309  2.36039
H   1.90411  1.54543  2.66503
H   1.48747  3.12267  3.30873
H   2.26489  3.05292  1.71989
C  -1.38375  1.57707  2.75753
H  -2.46055  1.63251  2.47233
H  -1.33856  2.02222  3.77485
H  -1.09722  0.51033  2.89942
C  -0.83074  4.37321  1.67649
H  -0.14698  5.07752  1.16162
H  -0.87126  4.67693  2.74461
H  -1.84593  4.51605  1.25308
C   1.51242  2.53961 -2.36135
H   1.90474  1.54142 -2.66742
H   2.26926  3.04707 -1.72039

```
H   1.49198  3.12040 -3.30898
C  -1.38356  1.58257 -2.76023
H  -1.10066  0.51479 -2.90178
H  -1.33577  2.02747 -3.77755
H  -2.46032  1.64192 -2.47581
C  -0.82117  4.37647 -1.67659
H  -1.83580  4.52306 -1.25320
H  -0.86028  4.68128 -2.74445
H  -0.13460  5.07756 -1.16100
C   5.19112  0.95044 -1.14767
H   5.24687  1.58688 -2.04547
C   5.19939  1.53785  0.13601
H   5.26130  2.63324  0.23996
C   5.18151  0.72007  1.28675
H   5.22946  1.17730  2.28824
C   5.15694 -0.68561  1.15335
H   5.19106 -1.32359  2.05118
C   5.14794 -1.27336 -0.13001
H   5.16865 -2.37017 -0.23364
C   5.16469 -0.45525 -1.28043
H   5.20367 -0.91300 -2.28208
```

**3^Mes**

SCF (BP86) Energy = -2496.64841466
Enthalpy 0K = -2495.850924
Enthalpy 298K = -2495.788204
Free Energy 298K = -2495.952482
Lowest Frequency = 8.9718 $cm^{-1}$
Second Frequency = 9.5044 $cm^{-1}$
SCF (Mesitylene) Energy = -2496.77251763
SCF (BP86-D3$^{BJ}$) Energy = -2496.91732519
SCF (BS2) Energy = -2498.55039975
SCF (BS3) Energy = -19004.22019783

```
Ba  2.06748 -0.22065  0.03238
Ba -2.06748  0.22060 -0.03256
Si -0.21794 -2.58459 -1.57974
Si -0.26152 -2.58498  1.57662
Si  0.26137  2.58494 -1.57681
Si  0.21807  2.58448  1.57957
N  -0.24502 -1.79274 -0.00160
N   0.24500  1.79269  0.00141
C   5.18311 -1.42149 -0.34167
H   5.27785 -2.48851 -0.60632
C   5.04728  0.91133 -1.01870
H   5.04414  1.67600 -1.81426
C   5.05358  1.32762  0.33592
C   5.10646  0.33219  1.34298
H   5.14508  0.64077  2.40176
C   5.19865 -1.04699  1.02457
C   5.12931 -0.45701 -1.37940
C   1.54984 -2.54230 -2.33732
H   1.93974 -1.54231 -2.63908
H   1.54592 -3.12716 -3.28259
H   2.29972 -3.04044 -1.68129
C  -1.33487 -1.58605 -2.77984
H  -1.04586 -0.51894 -2.91340
H  -2.41649 -1.64065 -2.51411
H  -1.26928 -2.03017 -3.79646
C  -0.78937 -4.38161 -1.68963
H  -0.10915 -5.08219 -1.16484
H  -0.81252 -4.68509 -2.75833
H  -1.80937 -4.53056 -1.28047
C  -0.83377 -4.38294  1.66842
H  -1.84195 -4.53224  1.23073
H  -0.88675 -4.68793  2.73560
H  -0.13911 -5.08254  1.16158
C  -1.41320 -1.58994  2.74465
H  -1.12996 -0.52252  2.88737
H  -1.37838 -2.03552  3.76212
H  -2.48491 -1.64595  2.44249
C   1.48379 -2.54033  2.38352
H   2.25334 -3.03620  1.74938
H   1.45478 -3.12606  3.32777
H   1.86241 -1.53963  2.69758
C   1.33484  1.58568  2.77962
H   1.04575  0.51858  2.91306
H   1.26924  2.02970  3.79628
H   2.41649  1.64022  2.51397
C  -1.54970  2.54251  2.33717
H  -2.29948  3.04079  1.68114
H  -1.54568  3.12734  3.28246
H  -1.93979  1.54258  2.63888
C   0.78992  4.38136  1.68954
H   1.80993  4.53008  1.28031
H   0.81324  4.68478  2.75826
H   0.10984  5.08214  1.16484
C  -1.48387  2.53973 -2.38388
H  -1.86208  1.53891 -2.69807
H  -1.45491  3.12552 -3.32809
H  -2.25371  3.03531 -1.74987
C   0.83298  4.38309 -1.66854
H   0.13804  5.08241 -1.16166
H   0.88584  4.68816 -2.73570
H   1.84109  4.53277 -1.23083
C   1.41338  1.59024 -2.74480
H   2.48504  1.64640 -2.44250
H   1.37860  2.03589 -3.76223
H   1.13034  0.52278 -2.88762
C  -5.18292  1.42125  0.34365
H  -5.27753  2.48796  0.60957
C  -5.19883  1.04834 -1.02302
C  -5.10681 -0.33048 -1.34306
H  -5.14568 -0.63783 -2.40219
C  -5.05377 -1.32708 -0.33716
C  -5.04709 -0.91236  1.01794
H  -5.04380 -1.67796  1.81260
C  -5.12892  0.45556  1.38024
C  -5.39953  2.08124 -2.10738
H  -4.89293  1.80490 -3.05361
H  -6.48323  2.16901 -2.34085
H  -5.05813  3.08882 -1.79789
C  -5.08558 -2.79523 -0.69430
H  -6.13406 -3.16484 -0.67092
H  -4.70252 -2.98897 -1.71574
H  -4.51548 -3.41482  0.02677
C  -5.24465  0.86650  2.82984
H  -6.31300  0.85129  3.13798
```

H  -4.70950  0.17281  3.50894
H  -4.87627  1.89642  3.00587
C   5.39917 -2.07861  2.11018
H   6.48287 -2.16638  2.34368
H   4.89271 -1.80097  3.05611
H   5.05747 -3.08648  1.80197
C   5.08517  2.79618  0.69137
H   4.70149  2.99109  1.71237
H   6.13366  3.16577  0.66821
H   4.51550  3.41493 -0.03075
C   5.24547 -0.86961 -2.82849
H   4.71067 -0.17661 -3.50855
H   6.31393 -0.85492 -3.13627
H   4.87699 -1.89967 -3.00348

**$4^{Mes}$**

SCF (BP86) Energy = -2250.32723372
Enthalpy 0K = -2249.750439
Enthalpy 298K = -2249.707546
Free Energy 298K = -2249.826372
Lowest Frequency = 23.5201 $cm^{-1}$
Second Frequency = 25.4880 $cm^{-1}$
SCF (Mesitylene) Energy =
-2250.36418343
SCF (BP86-$D3^{BJ}$) Energy = -2250.49865250
SCF (BS2) Energy = -2251.71049739
SCF (BS3) Energy = -2259.14358226

Ca  0.44475 -0.00167 -0.15119
Si -2.67509 -0.24549  1.46384
Si -2.35802  0.32586 -1.59562
N  -1.72978  0.04530  0.01142
C  -1.50804 -0.01838  2.96177
H  -2.00354 -0.32796  3.90654
H  -0.58415 -0.63534  2.87623
H  -1.20111  1.04162  3.08723
C  -3.37343 -2.01470  1.55089
H  -3.98553 -2.15412  2.46779
H  -4.02669 -2.23620  0.68092
H  -2.57227 -2.78397  1.57229
C  -4.14208  0.94455  1.67439
H  -4.65949  0.75922  2.64007
H  -3.83155  2.01036  1.66192
H  -4.89552  0.80610  0.86985
C  -3.60467 -0.95751 -2.22726
H  -3.87652 -0.75922 -3.28612
H  -3.21927 -1.99532 -2.16187
H  -4.54413 -0.91251 -1.63627
C  -3.12039  2.04419 -1.87175
H  -3.44075  2.17216 -2.92803
H  -4.01899  2.18571 -1.23535
H  -2.41328  2.86533 -1.63175
C  -0.80727  0.22827 -2.75582
H  -1.12304  0.41990 -3.80318
H  -0.02692  1.00306 -2.56128
H  -0.32315 -0.77459 -2.77396
C   3.50095  1.34384  2.37694
H   4.24829  0.61514  2.00634
H   4.07003  2.21863  2.76084
H   2.96796  0.90630  3.24430
C   2.55195  1.79711  1.29273
C   2.94207  1.79601 -0.06854
H   3.93370  1.40082 -0.34334
C   2.12605  2.36884 -1.07563
C   0.87470  2.91396 -0.69528
H   0.24106  3.38875 -1.46221
C   0.44025  2.92203  0.65387
C   1.28496  2.33499  1.62769
H   0.97438  2.35382  2.68511
C   2.61946  2.48158 -2.50018
H   3.33386  1.67563 -2.76007
H   1.78974  2.47175 -3.23477
H   3.15620  3.44582 -2.63534
C  -0.87679  3.54596  1.03973
H  -1.70503  2.82750  0.85908
H  -0.89598  3.82749  2.11040
H  -1.08431  4.45176  0.43613
C  -0.78884 -3.64954 -1.20126
H  -0.92618 -3.45655 -2.28268
H  -0.69859 -4.74971 -1.06988
H  -1.70198 -3.32814 -0.66305
C   0.44155 -2.96872 -0.65687
C   0.63221 -2.83512  0.74148
H  -0.15245 -3.19901  1.42424
C   1.82844 -2.30548  1.28324
C   2.82752 -1.84994  0.38575
H   3.77200 -1.45430  0.79259
C   2.67190 -1.95325 -1.01708
C   1.46432 -2.50156 -1.51776
H   1.34055 -2.61708 -2.60740
C   3.79203 -1.57823 -1.95916
H   3.41384 -1.14568 -2.90724
H   4.50530 -0.86645 -1.49959
H   4.37279 -2.48597 -2.23290
C   2.06689 -2.31486  2.77552
H   2.84940 -1.59311  3.07879
H   1.14263 -2.10172  3.34920
H   2.41330 -3.32305  3.09094

**$4^{Ben}$**

SCF (BP86) Energy = -2014.59579304
Enthalpy 0K = -2014.177278
Enthalpy 298K = -2014.145212
Free Energy 298K = -2014.241732
Lowest Frequency = 20.0907 $cm^{-1}$
Second Frequency = 24.2637 $cm^{-1}$
SCF (Benzene) Energy = -2014.63655646
SCF (BP86-$D3^{BJ}$) Energy = -2014.72175188
SCF (BS2) Energy = -2015.72401944
SCF (BS3) Energy = -2022.95778664

Ca -0.82686 -0.02515 -0.20125
Si  2.25004  0.24168  1.48300
Si  1.90658 -0.38804 -1.58590
N   1.31481 -0.06624  0.02449
C   1.06408  0.06710  2.97432
H   1.54950  0.42309  3.90798
H   0.12963  0.66383  2.86293
H   0.77650 -0.99224  3.14612
C   2.96510  2.00371  1.51363

| | | | |
|---|---|---|---|
| H | 3.54542 | 2.18175 | 2.44430 |
| H | 3.65151 | 2.17581 | 0.65806 |
| H | 2.17321 | 2.78153 | 1.46457 |
| C | 3.67879 | -0.98149 | 1.73215 |
| H | 4.16931 | -0.81685 | 2.71538 |
| H | 3.33314 | -2.03608 | 1.69981 |
| H | 4.46147 | -0.85952 | 0.95358 |
| C | 2.99158 | 0.97369 | -2.33435 |
| H | 3.22897 | 0.76459 | -3.39924 |
| H | 2.50862 | 1.97153 | -2.27877 |
| H | 3.95810 | 1.04305 | -1.79157 |
| C | 2.77011 | -2.06031 | -1.80911 |
| H | 3.00578 | -2.25484 | -2.87719 |
| H | 3.72836 | -2.08403 | -1.24941 |
| H | 2.15103 | -2.90241 | -1.43493 |
| C | 0.29430 | -0.46982 | -2.68394 |
| H | 0.60910 | -0.65008 | -3.73332 |
| H | -0.39631 | -1.31854 | -2.46092 |
| H | -0.29700 | 0.47613 | -2.73734 |
| C | -3.15186 | -1.50773 | 1.20675 |
| C | -3.41307 | -1.81642 | -0.14521 |
| H | -4.35558 | -1.49391 | -0.61453 |
| C | -2.47928 | -2.57114 | -0.88689 |
| C | -1.28587 | -3.01553 | -0.27735 |
| H | -0.56414 | -3.61782 | -0.85035 |
| C | -1.01993 | -2.69872 | 1.07131 |
| C | -1.95490 | -1.94480 | 1.81422 |
| H | -1.75714 | -1.71468 | 2.87270 |
| C | -0.66303 | 2.95667 | -0.36628 |
| C | -1.17334 | 2.72456 | 0.92951 |
| H | -0.56080 | 2.96952 | 1.81077 |
| C | -2.47243 | 2.19863 | 1.09391 |
| C | -3.26462 | 1.90483 | -0.03799 |
| H | -4.28870 | 1.52170 | 0.09009 |
| C | -2.75583 | 2.13577 | -1.33284 |
| C | -1.45670 | 2.66629 | -1.49589 |
| H | -1.07079 | 2.87551 | -2.50590 |
| H | -2.69354 | -2.83429 | -1.93489 |
| H | -0.09051 | -3.04836 | 1.54474 |
| H | -3.89266 | -0.94648 | 1.79725 |
| H | -3.38047 | 1.92906 | -2.21634 |
| H | 0.34727 | 3.37337 | -0.49161 |
| H | -2.87714 | 2.04360 | 2.10653 |

**5^Mes**

SCF (BP86) Energy = -1603.50569074
Enthalpy 0K = -1602.930179
Enthalpy 298K = -1602.886545
Free Energy 298K = -1603.009939
Lowest Frequency = 18.3913 $cm^{-1}$
Second Frequency = 20.4163 $cm^{-1}$
SCF (Mesitylene) Energy = -1603.54425464
SCF (BP86-D3$^{BJ}$) Energy = -1603.67103653
SCF (BS2) Energy = -1604.81643255
SCF (BS3) Energy = -4782.32525206

| | | | |
|---|---|---|---|
| Sr | 0.37621 | 0.00011 | -0.00052 |
| Si | -2.65909 | -0.21486 | 1.57913 |
| Si | -2.66004 | 0.21369 | -1.57875 |
| N | -1.96074 | -0.00060 | -0.00002 |
| C | 1.40405 | 2.59950 | 1.52394 |
| H | 1.18210 | 2.68652 | 2.60078 |
| C | 2.91874 | 1.95829 | -0.26651 |
| H | 3.88448 | 1.54175 | -0.59752 |
| C | 2.01952 | 2.46948 | -1.23676 |
| C | -3.86212 | 1.14964 | 2.12186 |
| H | -3.41235 | 2.16124 | 2.04405 |
| H | -4.19128 | 1.00247 | 3.17281 |
| H | -4.77487 | 1.14314 | 1.48870 |
| C | 0.79749 | 3.02796 | -0.79015 |
| H | 0.09438 | 3.44506 | -1.53023 |
| C | -1.15530 | -0.17913 | 2.79398 |
| H | -0.42796 | -1.00694 | 2.61245 |
| H | -1.48916 | -0.31857 | 3.84441 |
| H | -0.60850 | 0.79243 | 2.77984 |
| C | 0.47800 | 3.12104 | 0.58842 |
| C | -3.52495 | 1.88765 | -1.83142 |
| H | -4.38870 | 1.99624 | -1.14197 |
| H | -3.91297 | 1.99258 | -2.86730 |
| H | -2.84154 | 2.74273 | -1.64100 |
| C | 1.40609 | -2.59835 | -1.52410 |
| H | 1.18462 | -2.68503 | -2.60107 |
| C | 2.63995 | -2.02980 | -1.11775 |
| C | 2.91979 | -1.95725 | 0.26723 |
| H | 3.88528 | -1.54057 | 0.59882 |
| C | 2.02022 | -2.46888 | 1.23693 |
| C | 0.79858 | -3.02760 | 0.78961 |
| H | 0.09519 | -3.44500 | 1.52926 |
| C | 0.47979 | -3.12044 | -0.58914 |
| C | -3.86167 | -1.15206 | -2.12140 |
| H | -3.41053 | -2.16309 | -2.04410 |
| H | -4.19158 | -1.00500 | -3.17213 |
| H | -4.77409 | -1.14695 | -1.48776 |
| C | -0.78678 | -3.80674 | -1.03977 |
| H | -1.67066 | -3.43568 | -0.48288 |
| H | -0.71563 | -4.89963 | -0.85283 |
| H | -0.97107 | -3.66383 | -2.12182 |
| C | -1.15674 | 0.18015 | -2.79426 |
| H | -0.43048 | 1.00892 | -2.61281 |
| H | -1.49121 | 0.31931 | -3.84453 |
| H | -0.60862 | -0.79069 | -2.78057 |
| C | 2.63821 | 2.03112 | 1.11833 |
| C | 3.65849 | -1.59242 | -2.14523 |
| H | 4.24790 | -2.46977 | -2.48995 |
| H | 4.38123 | -0.86125 | -1.73276 |
| H | 3.18210 | -1.15671 | -3.04660 |
| C | -3.52202 | -1.88968 | 1.83296 |
| H | -4.38675 | -1.99901 | 1.14484 |
| H | -3.90833 | -1.99513 | 2.86943 |
| H | -2.83810 | -2.74407 | 1.64132 |
| C | 3.65634 | 1.59414 | 2.14640 |
| H | 3.17958 | 1.15832 | 3.04753 |
| H | 4.24527 | 2.47170 | 2.49141 |
| H | 4.37953 | 0.86318 | 1.73436 |
| C | -0.78942 | 3.80637 | 1.03815 |
| H | -1.67353 | 3.42912 | 0.48572 |
| H | -0.72227 | 4.89829 | 0.84438 |
| H | -0.97059 | 3.66925 | 2.12147 |
| C | 2.39204 | 2.49241 | -2.70187 |

```
H   3.08067  1.66737 -2.97175
H   1.50306  2.44317 -3.36166
H   2.91797  3.44235 -2.94084
C   2.39215 -2.49205  2.70218
H   3.07790 -1.66498  2.97325
H   2.92125 -3.44044  2.94037
H   1.50273 -2.44651  3.36160
```

**5Ben**

SCF (BP86) Energy = -1367.77318673
Enthalpy 0K = -1367.355928
Enthalpy 298K = -1367.323205
Free Energy 298K = -1367.425218
Lowest Frequency = 5.1437 $cm^{-1}$
Second Frequency = 8.2296 $cm^{-1}$
SCF (Benzene) Energy = -1367.81561827
SCF (BP86-D3$^{BJ}$) Energy = -1367.89455317
SCF (BS2) Energy = -1368.82919662
SCF (BS3) Energy = -4546.13976928

```
Sr -0.70704  0.00041 -0.00010
Si  2.27043  0.00646  1.60760
Si  2.27081 -0.00216 -1.60761
N   1.60452  0.00099 -0.00008
C   0.71830  0.00708  2.76953
H   1.03244  0.00632  3.83517
H   0.07974  0.91668  2.66207
H   0.07948 -0.90235  2.66097
C   3.28093  1.55333  2.03567
H   3.59308  1.55436  3.10186
H   4.20498  1.60217  1.42152
H   2.70998  2.48668  1.84522
C   3.28499 -1.53554  2.04359
H   3.59785 -1.53018  3.10956
H   2.71598 -2.47122  1.85881
H   4.20863 -1.58570  1.42894
C   3.28253  1.54198 -2.04262
H   3.59620  1.53730 -3.10835
H   2.71125  2.47638 -1.85834
H   4.20560  1.59417 -1.42728
C   3.28430 -1.54696 -2.03609
H   3.59450 -1.54851 -3.10284
H   4.20957 -1.59291 -1.42355
H   2.71596 -2.48144 -1.84344
C   0.71890 -0.00496 -2.76975
H   1.03303 -0.00064 -3.83539
H   0.08313 -0.91670 -2.66425
H   0.07729  0.90233 -2.65917
C  -3.15762 -2.03279  0.82978
C  -3.28643 -1.96330 -0.57355
H  -4.19874 -1.54110 -1.02357
C  -2.26256 -2.47145 -1.40174
C  -1.10989 -3.04997 -0.82647
H  -0.31983 -3.46622 -1.47071
C  -0.98029 -3.11866  0.57676
C  -2.00508 -2.61053  1.40478
H  -1.91924 -2.69269  2.49995
C  -0.97560  3.13039 -0.52691
C  -1.15243  3.03568  0.86968
H  -0.38781  3.44575  1.54774
C  -2.32007  2.44000  1.39517
C  -3.31202  1.94098  0.52373
H  -4.23627  1.50528  0.93479
C  -3.13626  2.03667 -0.87298
C  -1.96869  2.63158 -1.39812
H  -1.84666  2.73453 -2.48805
H  -2.37605 -2.44373 -2.49728
H  -0.08893 -3.58556  1.02351
H  -3.96911 -1.66534  1.47772
H  -3.92295  1.67615 -1.55455
H  -0.07274  3.61047 -0.93485
H  -2.47006  2.39177  2.48562
```

**6Mes**

SCF (BP86) Energy = -1598.29078878
Enthalpy 0K = -1597.715617
Enthalpy 298K = -1597.671882
Free Energy 298K = -1597.797518
Lowest Frequency = 9.2259 $cm^{-1}$
Second Frequency = 15.9708 $cm^{-1}$
SCF (Mesitylene) Energy =
-1598.33570579
SCF (BP86-D3$^{BJ}$) Energy = -1598.45318154
SCF (BS2) Energy = -1599.62193904
SCF (BS3) Energy = -9852.70518326

```
Ba  0.39546 -0.00012 -0.00114
Si -2.79543  0.16275 -1.58256
Si -2.79396 -0.17139  1.58300
N  -2.09139 -0.00252  0.00007
C   1.45063 -2.82791 -1.47820
H   1.22533 -2.91793 -2.55449
C   2.69579 -2.28256 -1.07733
C   2.97675 -2.20013  0.30681
H   3.94870 -1.79438  0.63469
C   2.06166 -2.66998  1.28124
C   0.82835 -3.20817  0.84000
H   0.11075 -3.59141  1.58492
C   0.51121 -3.31503 -0.53702
C  -0.76868 -3.98078 -0.98347
H  -1.00962 -3.74941 -2.03910
H  -1.63043 -3.68170 -0.35394
H  -0.67103 -5.08505 -0.90082
C   2.42114 -2.66089  2.75005
H   3.13716 -1.85295  3.00092
H   2.91000 -3.62063  3.02618
H   1.52873 -2.55922  3.39962
C   3.72174 -1.86289 -2.10555
H   3.25132 -1.49865 -3.04102
H   4.40322 -1.07881 -1.71934
H   4.35715 -2.73133 -2.38546
C   2.04936  2.67871 -1.28063
C   0.81391  3.21145 -0.83889
H   0.09476  3.59246 -1.58346
C   0.49639  3.31581  0.53825
C   1.43787  2.83186  1.47897
H   1.21227  2.92012  2.55533
C   2.68522  2.29191  1.07759
C   2.96641  2.21171 -0.30663
H   3.93997  1.81016 -0.63492
```

```
C  -0.78658  3.97532  0.98506
H  -0.69683  5.07976  0.89629
H  -1.02288  3.74765  2.04252
H  -1.64804  3.66695  0.35956
C   2.40893  2.67249 -2.74945
H   1.51689  2.56854 -3.39917
H   3.12771  1.86726 -3.00123
H   2.89458  3.63417 -3.02446
C   3.71302  1.87569  2.10536
H   3.24426  1.50955  3.04092
H   4.34544  2.74635  2.38522
H   4.39719  1.09417  1.71872
C  -1.31192  0.15782 -2.82926
H  -0.71469 -0.78527 -2.81782
H  -0.62402  1.03001 -2.71346
H  -1.69995  0.24053 -3.86720
C  -3.71166  1.80415 -1.86318
H  -4.59134  1.88800 -1.19076
H  -4.08310  1.89036 -2.90685
H  -3.05925  2.68097 -1.66325
C  -3.94662 -1.25686 -2.09644
H  -3.45454 -2.24698 -1.99746
H  -4.28246 -1.14520 -3.14966
H  -4.85757 -1.27516 -1.46118
C  -3.94880  1.24448  2.09905
H  -3.45944  2.23607  2.00147
H  -4.28422  1.13039  3.15214
H  -4.85986  1.26120  1.46391
C  -3.70569 -1.81546  1.86266
H  -4.58576 -1.90094  1.19097
H  -4.07596 -1.90360  2.90659
H  -3.05124 -2.69043  1.66132
C  -1.30957 -0.16381  2.82858
H  -0.62007 -1.03459  2.71182
H  -1.69654 -0.24766  3.86682
H  -0.71422  0.78046  2.81685
```

**$6^{Ben}$**

SCF (BP86) Energy = -1362.55852708
Enthalpy 0K = -1362.141464
Enthalpy 298K = -1362.108713
Free Energy 298K = -1362.211873
Lowest Frequency = 6.7832 cm$^{-1}$
Second Frequency = 9.5079 cm$^{-1}$
SCF (Benzene) Energy = -1362.60778379
SCF (BP86-D3$^{BJ}$) Energy = -1362.67918701
SCF (BS2) Energy = -1363.63500239
SCF (BS3) Energy = -9616.52159716

```
Ba  0.74075 -0.00025 -0.00032
Si -2.41228  0.00644  1.59971
Si -2.41302 -0.00133 -1.59926
N  -1.72047  0.00218  0.00007
C  -0.90581  0.01129  2.82244
H  -1.28742  0.01575  3.86607
H  -0.26158 -0.89925  2.76329
H  -0.26352  0.92262  2.75448
C  -3.43496 -1.53684  2.01406
H  -3.77091 -1.52887  3.07303
H  -4.34500 -1.58993  1.37986
H  -2.86045 -2.47208  1.84485
C  -3.43419  1.55271  2.00476
H  -3.77042  1.55165  3.06366
H  -2.85807  2.48602  1.83033
H  -4.34382  1.60335  1.36979
C  -3.43789 -1.54589 -2.00340
H  -3.77452 -1.54452 -3.06218
H  -2.86346 -2.48022 -1.82890
H  -4.34737 -1.59465 -1.36808
C  -3.43310  1.54366 -2.01363
H  -3.76911  1.53612 -3.07259
H  -4.34302  1.59852 -1.37941
H  -2.85686  2.47787 -1.84464
C  -0.90718 -0.00868 -2.82272
H  -1.28937 -0.01441 -3.86613
H  -0.26288  0.90189 -2.76502
H  -0.26495 -0.92002 -2.75411
C   3.03864  2.40706  1.00527
C   3.38776  2.25185 -0.35257
H   4.37673  1.85036 -0.62517
C   2.48283  2.64360 -1.36209
C   1.22995  3.19314 -1.01359
H   0.53376  3.52401 -1.80048
C   0.88018  3.34883  0.34448
C   1.78503  2.95480  1.35371
H   1.52520  3.10431  2.41398
C   0.87141 -3.34823 -0.35774
C   1.20968 -3.19920  1.00396
H   0.50533 -3.53065  1.78330
C   2.46136 -2.65547  1.36567
C   3.37656 -2.26295  0.36573
H   4.36448 -1.86604  0.64865
C   3.03891 -2.41152 -0.99575
C   1.78648 -2.95336 -1.35740
H   1.53544 -3.09764 -2.42051
H   2.76590  2.54917 -2.42288
H  -0.08963  3.79456  0.61514
H   3.75536  2.12801  1.79426
H   3.76354 -2.13184 -1.77726
H  -0.09753 -3.78945 -0.63870
H   2.73537 -2.56622  2.42928
```

**Benzene**

SCF (BP86) Energy = -232.076219335
Enthalpy 0K = -231.978535
Enthalpy 298K = -231.973084
Free Energy 298K = -232.004379
Lowest Frequency = 401.2667 cm$^{-1}$
Second Frequency = 401.2669 cm$^{-1}$
SCF (Benzene) Energy = -232.077590844
SCF (BP86-D3$^{BJ}$) Energy = -232.093113770
SCF (BS2) Energy = -232.326587265
SCF (BS3) Energy = -232.504759678

```
C   1.21823  0.70334  0.00000
C   0.00000  1.40669  0.00000
C   1.21823 -0.70334  0.00000
H   0.00000  2.50866  0.00000
H   2.17256 -1.25433  0.00000
C  -1.21823  0.70334 -0.00000
```

```
C  -0.00000 -1.40669 -0.00000
H  -2.17256  1.25433  0.00000
H  -0.00000 -2.50866  0.00000
C  -1.21823 -0.70334  0.00000
H  -2.17256 -1.25433  0.00000
H   2.17256  1.25433  0.00000
```

**Mesitylene**

SCF (BP86) Energy = -349.936139262
Enthalpy 0K = -349.759573
Enthalpy 298K = -349.748447
Free Energy 298K = -349.796772
Lowest Frequency = 32.6169 $cm^{-1}$
Second Frequency = 34.0647 $cm^{-1}$
SCF (Mesitylene) Energy =
-349.937587048
SCF (BP86-$D3^{BJ}$) Energy = -349.967825292
SCF (BS2) Energy = -350.314816492
SCF (BS3) Energy = -350.586634062

```
C   0.45797  2.89940 -0.00015
H   0.00114  3.37925 -0.89247
H  -0.00121  3.37981  0.89065
H   1.53778  3.14916  0.00116
C   0.22921  1.40314 -0.00001
C  -1.08557  0.88195  0.00019
H  -1.94158  1.57890  0.00034
C  -1.32978 -0.50310  0.00040
C  -0.22104 -1.38114  0.00056
H  -0.39662 -2.47094  0.00103
C   1.10055 -0.90009  0.00030
C   1.30659  0.49912  0.00007
H   2.33817  0.89196  0.00014
C  -2.73998 -1.05306 -0.00039
H  -3.49613 -0.24281  0.00977
H  -2.92967 -1.68087 -0.89769
H  -2.92403 -1.69861  0.88531
C   2.28204 -1.84627 -0.00032
H   2.92192 -1.69497 -0.89638
H   2.93173 -1.68429  0.88666
H   1.95854 -2.90630  0.00757
```
